# A rapid systematic review and evidence synthesis of effective coverage measures and cascades for childbirth, newborn and child health in low- and middle-income countries

**DOI:** 10.7189/jogh.12.04001

**Published:** 2022-01-15

**Authors:** Josephine Exley, Prateek Anand Gupta, Joanna Schellenberg, Kathleen L Strong, Jennifer Harris Requejo, Ann-Beth Moller, Allisyn C Moran, Tanya Marchant, Child Health Accountability Tracking Technical Advisory Group (CHAT) and the Mother and Newborn Information for Tracking Outcomes and Results Technical Advisory Group (MoNITOR)

**Affiliations:** 1 Department of Disease Control, London School of Hygiene and Tropical Medicine, London, UK; 2 Department of Global Health and Development, London School of Hygiene and Tropical Medicine, London, UK; 3 Department of Maternal, Newborn, Child and Adolescent Health, World Health Organization, Geneva, Switzerland; 4 Division of Data, Analytics, Planning & Monitoring, United Nations Children’s Fund, New York, USA; 5 UNDP/UNFPA/UNICEF/WHO/World Bank Special Programme of Research, Development and Research Training in Human Reproduction (HRP), Department of Reproductive Health and Research, World Health Organization, Geneva, Switzerland

## Abstract

**Background:**

Effective coverage measures aim to estimate the proportion of a population in need of a service that received a positive health outcome. In 2020, the Effective Coverage Think Tank Group recommended using a ‘coverage cascade’ for maternal, newborn, child and adolescent health and nutrition (MNCAHN), which organises components of effective coverage in a stepwise fashion, with each step accounting for different aspects of quality of care (QoC), applied at the population level. The cascade outlines six steps that increase the likelihood that the population in need experience the intended health benefit: 1) the population in need (target population) who contact a health service; 2) that has the inputs available to deliver the service; 3) who receive the health service; 4) according to quality standards; 5) and adhere to prescribed medication(s) or health workers instructions; and 6) experience the expected health outcome. We examined how effective coverage of life-saving interventions from childbirth to children aged nine has been defined and assessed which steps of the cascade are captured by existing measures.

**Methods:**

We undertook a rapid systematic review. Seven scientific literature databases were searched covering the period from May 1, 2017 to July, 8 2021. Reference lists from reviews published in 2018 and 2019 were examined to identify studies published prior to May 2017. Eligible studies reported population-level contact coverage measures adjusted for at least one dimension of QoC.

**Results:**

Based on these two search approaches this review includes literature published from 2010 to 2021. From 16 662 records reviewed, 33 studies were included, reporting 64 effective coverage measures. The most frequently examined measures were for childbirth and immediate newborn care (n = 24). No studies examined measures among children aged five to nine years. Definitions of effective coverage varied across studies. Key sources of variability included (i) whether a single effective coverage measure was reported for a package of interventions or separate measures were calculated for each intervention; (ii) the number and type of coverage cascade steps applied to adjust for QoC; and (iii) the individual items included in the effective coverage definition and the methods used to generate a composite quality measure.

**Conclusion:**

In the MNCAHN literature there is substantial heterogeneity in both definitions and construction of effective coverage, limiting the comparability of measures over time and place. Current measurement approaches are not closely aligned with the proposed cascade. For widespread adoption, there is a need for greater standardisation of indicator definitions and transparency in reporting, so governments can use these measures to improve investments in MNACHN and implement life-saving health policies and programs.

Maternal, newborn, child and adolescent health and nutrition (MNCAHN) is a key priority for the global health and development agenda [1]. Maternal, newborn and child deaths are mostly preventable as the interventions that prevent or treat the major causes of ill health are known. However, deaths in these populations remain unacceptably high and disproportionately occur in low- and middle-income countries (LMICs) [2]. Improving both accessibility to and the quality of effective interventions is key to improving health outcomes for women and children [3].

Efforts to improve MNCAHN globally have been supported by the tracking of global and national health goals, including the Sustainable Development Goals, that typically measure contact coverage, defined as the proportion of a population in need of a service or intervention that received the service [1,4-6]. There is evidence that contact coverage indicators overestimate the health benefits of an intervention or service as they do not adequately capture the quality of care (QoC) delivered [7-10]. Effective coverage measures that move beyond contact coverage by also accounting for QoC, are now recommended as best practice [3,11,12]. Effective coverage indicators estimate the proportion of a population in need of a service that received the service with sufficient quality to achieve a positive health outcome. In this way they aim to better estimate the true benefit of an intervention or service [3,7,12-14].

Measurement of QoC is challenging as multiple dimensions need to be examined. QoC can be measured in terms of inputs (eg, adequacy of facilities, equipment and resources, trained and adequate number of health care professionals), processes (eg, appropriate use of effective clinical and non-clinical interventions) and outcomes (eg, avoidable mortality and morbidity, improved health and well-being) [15]. Increasingly, there is also a focus on a patient-centred approach, which considers experience of care and the right to be treated with respect [3,16,17]. Studies have used various indicators to measure QoC and several definitions for effective coverage have been proposed [7,11,12,14,18-21].

In 2019, the World Health Organization (WHO) and the United Nations Children’s Fund (UNICEF) convened a group of experts – the Effective Coverage Think Tank Group - to establish standardised definitions and measurement approaches of effective coverage for MNCAHN. This expert group considered findings from two previous reviews of effective coverage measures and applications [7,13] and recommended the adoption of a ‘health-service coverage cascade’, presented in **Figure 1** [22]. This cascade outlines six steps, presented sequentially for analytical purposes, with each step accounting for different dimensions of QoC: 1) the population in need (target population) who contact a health service; 2) that has the inputs available to deliver the service; 3) who receive the health service; 4) according to quality standards (referred to in this paper as ‘process quality’); 5) and adhere to prescribed medication(s) or health workers instructions; and 6) experience the expected health outcome.

**Figure 1:**
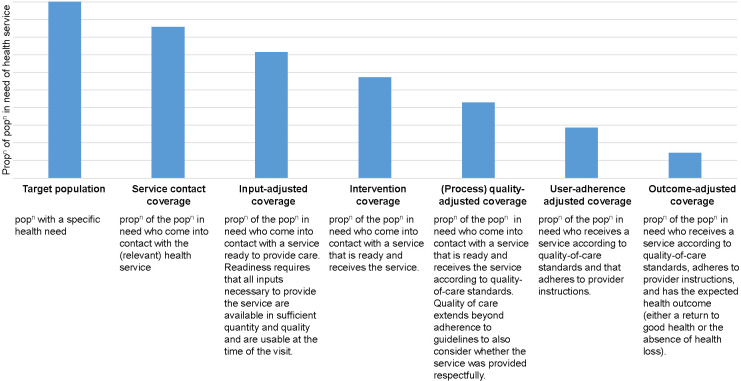
Health service coverage cascade for measuring effective coverage. Adapted from Marsh et al. 2020 [22].

Effective coverage is, ideally, estimated as the final step of the cascade and incorporates all previous steps into one summary measure. However, the feasibility of measuring outcome-adjusted coverage depends upon the type of intervention and is most suitable where the health impact can be directly linked to an intervention (eg, treatment of children with severe malnutrition with specially formulated foods). Conversely, some services, such as childbirth care, integrate multiple interventions into a single health contact making outcome-adjusted coverage challenging to estimate directly. Here process quality-adjusted coverage may be a more suitable proxy measurement of effective coverage.

Discussions regarding the use of this coverage cascade for tracking MNCAHN services have been largely conceptual. Challenges remain in operationalising the cascade (including defining the content and data source of each cascade step), providing guidance for linking data from multiple sources to calculate each step, and ensuring that the cascade is responsive to the needs of different types of decision makers, including programme managers and policy makers [22]. If effective coverage measures are to have wide-scale uptake, then there is a need for more guidance on how they can be constructed and used to identify health service strengths and weaknesses.

We report results from a rapid systematic review and evidence synthesis examining how effective coverage measures of life-saving interventions from childbirth to children up to nine years of age have been defined in the literature. This review specifically sought to map the individual items and data sources used to construct effective coverage measures against the steps of the coverage cascade, to identify which steps of the cascade contact coverage have been adjusted for, and to reflect on the implications for the widespread adoption of the proposed cascade.

## METHODS

We applied a rapid review approach, following standardised methods and reported in accordance with PRISMA guidelines [23-26].

### Information sources and search strategy

The two earlier reviews of effective coverage that informed the development of the coverage cascade provided the basis of our search strategy [7,13]. These two reviews examined the types of interventions assessed using effective coverage and the size of the gap between contact coverage and quality-adjusted coverage measures. Our review examines the content of those measures in detail. We searched the reference lists of the two earlier reviews to identify potentially relevant studies. To identify articles published since the two earlier reviews, seven databases were searched: Embase (using OVID); MEDLINE (using OVID); ProQuest, ScienceDirect, Scopus, SpringerLink, and Web of Science covering the period from May 1, 2017 to July 8, 2021. We devised the search syntax by updating the search strategy from the two earlier reviews related to two concepts: 1) effective coverage; AND 2) the target population and/or intervention. Searches were restricted to studies in countries categorised as LMICs by the World Bank at the time of the search and all search terms used were in English [27]. The complete list of search terms used for the EMBASE and MEDLINE searches are presented in Table S1 in in the **Online Supplementary Document**.

Additional studies were identified through citation searches, conducted using the ‘cited by’ function in Google Scholar to identify subsequent studies that had cited the reviews. To ensure no key publications were missing members of the Child Health Accountability Tracking Technical Advisory Group (CHAT) [28] and study authors, including representatives from the Mother and Newborn Information for Tracking Outcomes and Results Technical Advisory Group (MoNITOR) [29], were consulted.

### Eligibility criteria

Studies conducted in any LMIC that measure a population-level adjusted contact coverage estimate of life-saving interventions from childbirth to children up to nine years of age were eligible for inclusion. The age range was selected to capture interventions of interest to CHAT, which is tasked with standardising global monitoring indicators measuring the health of children aged 1 month to 9 years. Due to interest among the study authors’ and the links between maternal, newborn and child health, this age range was expanded to include interventions around childbirth and the immediate newborn period. Eligible studies needed to combine at least three components of effective coverage: population in need, service use and at least one other dimension from the coverage cascade. No restrictions were placed on the definition of QoC applied by the author or the data source(s) used as long as population-level measures were derived. The inclusion and exclusion criteria are summarised in **Table 1**.

**Table 1:** Inclusion and exclusion criteria

	Inclusion	Exclusion
Population/setting	• Studies conducted in any low- or middle-income setting.	• Studies conducted in high income settings.
	• Studies that defined the target population in need of a health service or intervention.	• Studies that did not define and quantify the target population in need.
	• Studies conducted among women during childbirth, newborns and children up to 9 y of age.	
	• Studies conducted in health facilities, communities or home.	
Interventions	Studies that examined essential life-saving interventions provided during childbirth through childhood up to 9 years of age [30]:	
	• Childbirth and postnatal care eg, social support, prevention of postpartum haemorrhage, induction of labour, management of postpartum haemorrhage, HIV therapy.	
	• Immediate essential newborn care eg, thermal protection, immediate drying and additional stimulation, neonatal resuscitation, clean cord care.	
	• Small and sick babies eg, kangaroo mother care, extra support for feeding small and preterm baby, prophylactic and therapeutic use of surfactant, management of jaundice.	
	• Infancy and childhood eg, exclusive breastfeeding for first 6 mo, complementary feeding, prevention and management of malaria, care for HIV, management of acute malnutrition, management of pneumonia, management of diarrhoea, management of meningitis, routine immunization, Vitamin A supplementation from 6 mo.	
Outcome measures	• Any study that presented the methods used to measure a population-level adjusted contact coverage measure.	• Studies that do not provide sufficient detail on the items used to construct the effective coverage measure in the paper, appendices, or other supporting information.
	• Studies needed to define the following three components:	• Studies that do not measure all three components of effective coverage (need, use, quality of care).
	Need: population in need of the intervention or service.	
	Use: population that comes into contact with a service or received a specific intervention; AND.	
	Quality of care: at least one dimension of QoC as defined by the study authors, can include inputs or process measures of quality as well as health outcomes.	•
Comparisons	n/a	
Study design	• Studies using any study design or data source to estimate effective coverage.	• Commentaries and editorials
	• Abstracts and conference presentations, if enough data presented to determine how effective coverage measure constructed.	• Reviews
		• Technical reports
Language	• Studies published in English	• Studies not published in English

### Selection process

Retrieved title and abstract records were loaded into the reference manager programme Endnote X7 and duplicate references were removed [31]. Two reviewers (JE and PG) double screened 15% of the records to ensure consistency in selection between the reviewers (Cronbach's alpha = 0.86). The two reviewers independently screened the remaining titles and abstracts (either JE or PG).

Full-texts of potentially relevant studies identified from the title and abstract screening were obtained and screened by both reviewers (JE and PG), with any uncertainties discussed between the two reviewers. Where we were unable to access the full-text, the study authors were contacted via email. The reason for excluding studies based on full-text review was recorded.

### Data collection process and risk of bias assessment

Study information was extracted into a standardised table to capture data on how effective coverage measures were constructed and defined, which individual items were included, the methods for construction of any composite scores and the data sources used.

Given the review’s focus, data on the study results was not extracted and a formal quality assessment or risk of bias assessment was not undertaken. Information from included studies was extracted by one reviewer and checked by a second reviewer (JE or PG).

### Synthesis

Studies were grouped by population group (women, newborns, children under five and children aged five to nine) and intervention or health service type. For each group of studies, we extracted the individual items used to construct the effective coverage measure and mapped these against the seven steps of the coverage cascade presented in **Figure 1**. To ensure consistency in our approach to this mapping, we classified items based on definitions outlined in **Box 1**. Evidence is summarised in a narrative synthesis with data presented in tables.

Box 1 **Definitions of the seven steps of the coverage cascade used to synthesis evidence across included studies.**

**Target population:** individuals in need of a health service or intervention based on belonging to either a specific group eg, pregnant women or the presence of a specific disease/condition eg, child with fever.

**Service contact:** individuals who sought or received needed care.

**Inputs:** health service readiness to provide care, includes facility infrastructure, availability and competence of staff, availability of supplies and commodities.

**Intervention:** receipt of clinical and non-clinical interventions administered to provide a direct health benefit.

**Process (quality):** receipt of interventions and behaviours that enhance interactions, including effective communication, respectful care and emotional support.

**User-adherence:** service user adheres to prescribed medications or provider instructions.

**Outcome:** health outcome.

## RESULTS

**Figure 2:**
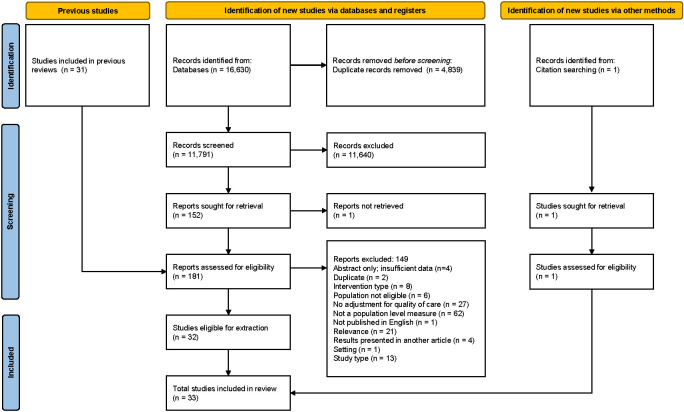
PRISMA diagram.

Database searches identified 16 630 records (**Figure 2**). After removal of duplicates, we screened 11 791 records of which 151 were considered for full-text review. In addition, 32 papers were identified through other methods for full-text review. Of those papers assessed in the full-text review 33 studies were identified as eligible for inclusion. Table S2 in the **Online Supplementary Document** lists reference details of excluded studies and reasons for exclusion based on the full-text review.

### Summary of included studies

A complete description of included studies is presented in Table S3 in the **Online Supplementary Document**. **Table 2** presents an overview of the number of studies reporting effective coverage measures by the type of service or intervention and population group. The most frequently examined interventions were facility-based childbirth and immediate newborn care, followed by sick child care. The majority of studies were conducted among women and newborns; we identified no studies that included children aged five to nine years.

**Table 2:** Number of studies that constructed an effective coverage measure for each health service or intervention by population group

Women and newborns	Children under 5	Children aged 5 to 9
Facility based childbirth and/or immediate newborn care, n = 17	Sick child care, n = 10	No studies identified
Postnatal care for women and/or newborn, n = 8	Complementary feeding, n = 5	
Care of sick newborns, n = 1	Growth monitoring, n = 1	
Exclusive breastfeeding, n = 1	Insecticide treated bednets (ITN), n = 1	
	Vaccines, n = 4	

**Figure 3:**
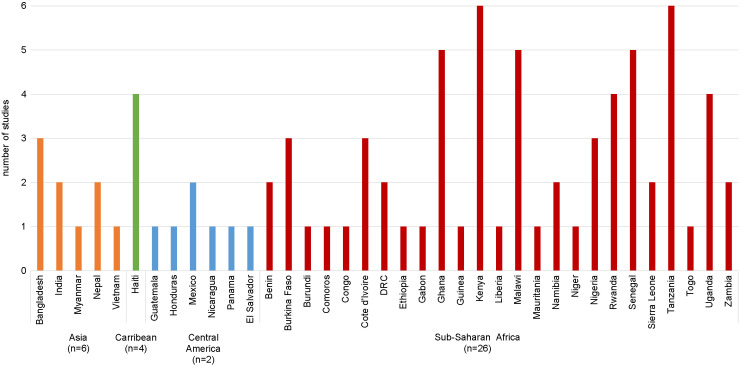
Number of studies conducted by country and region. n indicates the total number of studies that included countries from the region. Six studies included multiple countries in the analysis; four included countries across multiple regions [32-35] and two included countries from a single region [36,37].

**Figure 4:**
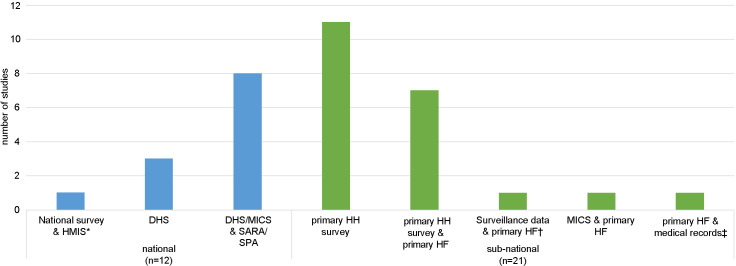
Number of studies by data source used to construct effective coverage measure and level of data collection. Primary HH survey and primary HF indicate primary data collection undertaken by the study authors. Two studies conducted observations of care as part of the HF survey [38,39] and four studies included observations collected as part of SPA [33,40-42]. *One study examining childbirth, immediate newborn care and sick child care, used the Mexican National Health and Nutrition Survey (ENSANUT) and routine data: the Mexican Institute of Social Security (IMSS) performance indicators from health management information systems [43]. †Surveillance data refers to demographic surveillance data collected as part of the Newhints trial [44]. ‡One study of sick newborns did not include a household survey, instead authors estimated the population in need by applying the rate of live births requiring inpatient services to the total number of live births extrapolated from the DHS [45]. DHS – Demographic Health Survey, HF – health facility survey, HH – household survey, IMSS – Mexican Institute of Social Security performance indicators from health management information systems, MICS – Multiple Indicator Cluster Surveys, SARA – Service Availability and Readiness Assessment, SPA – Service Provision Assessment.

The majority of studies were conducted in a single country (27 out of 33) (**Figure 3**), six studies were conducted across multiple countries, four of which included countries across different regions of the world [32-37]. Studies were most frequently conducted in countries in sub-Saharan Africa (27 out of 33); Kenya and Tanzania were the most frequently studied countries (6 out of 33), while 17 countries were included in only one study. The majority of studies used primary data collected at a sub-national level (**Figure 4**). For the 12 studies conducted at the national level, one conducted in Mexico used the nationally representative Mexican National Health and Nutrition Survey (ENSANUT) and routine health information from the Mexican Institute of Social Security (IMSS) [43]. The rest used nationally representative household surveys (Demographic and Health Surveys [DHS] and/or Multiple Indicator Cluster Survey [MICS]) and health facility assessments (Service Provision Assessment [SPA] and/or Service Availability and Readiness Assessment [SARA]). Fourteen studies used a single source of data (DHS or primary household surveys) and one study of sick newborns estimated the population in need by applying the rate of newborns needing inpatient care to an estimate of the number of live births in the study area [45]. The remaining 18 studies linked two or more sources of data, most frequently household and health facility data. Six studies also included direct observations of clinical care either from SPA [33,40-42] or as part of a primary health facility assessment [38,39].

### Definition and construction of effective coverage measures

Overall 64 measures that met the eligibility criteria were reported across the 33 studies; 36 measures of interventions among women and newborns (**Table 3**), 29 measures among children under five (**Table 4**), and 0 measures for children ages five to nine. Seventeen studies explicitly defined the measures as effective coverage, three studies referred to measures as effective coverage but reported them according to the adjustments made: input-adjusted coverage [50,55] and structure-adjusted coverage and process-adjusted coverage [39]. The remaining studies did not use the term effective coverage, instead measures were defined as: adequate contact with high quality care [52], content coverage [36], coverage of obstetric services [32], facility readiness [48], high quality contacts [34], missed opportunities [37], population access to quality care [42], quality coverage [64], quality-adjusted contact [51], quality-adjusted coverage [40] and treatment pathway [56,57].

**Table 3:** Mapping effective coverage measures for women and newborns against the steps of the coverage cascade: by health service or intervention type and the data source and the number of items to measure each step

Reference	Author reported measure	Health service or intervention	Target population	Service contact coverage	Input-adjusted coverage	Intervention coverage	Process quality-adjusted coverage	User-adherence adjusted coverage	Outcome-adjusted coverage
**Childbirth &/or immediate newborn care (Table S4 in the ** **Online Supplementary Document** **)**									
Nesbitt et al. 2013 [44]	Effective coverage	Intrapartum & immediate newborn care	Surveillance*	Surveillance*	HF, n = 20*	HF (HCW), n = 24*	HF (HCW), n = 3*	‡	‡
Larson et al. 2017 [46]	Effective coverage	Obstetric care	HH*	HH*	HF, n = 37*	HF records, n = 4*	HF records, n = 2*	‡	‡
Baker et al. 2015 [47]	Effective coverage	Active management of third stage of labour	HH*	HH*	HF, n = 2*	HF (HCW), n = 1*	†	‡	‡
	Effective coverage	Use of partograph to monitor labour	HH*	HH*	HF, n = 1*	†	HF (HCW), n = 1*	‡	‡
Munos et al. 2018 [39]	Process-adjusted coverage	Labour & delivery	MICS*	MICS*	HF, n = 9*	†	HF (HCW), n = 10*	‡	‡
Kanyangarara et al. 2018 [32]	Coverage of obstetric services: readiness	Obstetric service	DHS/MICS*	DHS/MICS, n = 1*	SPA/SARA, n = 23*	†	†	‡	‡
	Coverage of obstetric services: service availability	Obstetric service	DHS/MICS*	DHS/MICS*	SPA/SARA, n = 9*	†	†	‡	‡
Kemp et al. 2018 [48]	Facility readiness	Facility delivery	DHS*	DHS*	SARA, n = 70*	†	†	‡	‡
Wang et al. 2019 [35]	Effective coverage	Facility delivery	DHS*	DHS*	SPA, n = 53*	†	†	‡	‡
Munos et al. 2018 [39]	Structure-adjusted coverage	Labour & delivery	MICS*	MICS*	HF, n = 33*	†	†	‡	‡
Sharma et al. 2017 [42]	Population access to quality infrastructure	Delivery care	DHS*	DHS*	SPA, n = 20*	†	†	‡	‡
Willey et al. 2018 [49]	Effective coverage	Basic emergency obstetric care	HH*	HH*	HF, n = 18*	†	†	‡	‡
Nguhiu et al. 2017 [41]	Effective coverage	Skilled delivery & perinatal care	DHS*	DHS*	SPA, n = 9*	†	†	‡	‡
Munos et al. 2018 [39]	Structure-adjusted coverage	Immediate newborn care	MICS*	MICS*	HF, n = 9*	†	†	‡	‡
Nguyen et al. 2021 [50]	Input-adjusted coverage	Birth care	DHS*	DHS*	SPA, n = 5*	†	†	‡	‡
Sharma et al. 2017 [42]	Population access to quality care	Delivery care	DHS*	DHS*	†	SPA, n = 12*	SPA, n = 6*	‡	‡
Munos et al. 2018 [39]	Process-adjusted coverage	Immediate newborn care	MICS*	MICS*	†	HF (HCW), n = 17*	HF (HCW), n = 2*	‡	‡
Okawa et al. 2019a [51]	Quality-adjusted contact	Peripartum care	HH*	HH*	†	HH, n = 6*	HH, n = 1*	‡	‡
Joseph et al. 2020 [40]	Quality-adjusted coverage	Post-delivery care	MICS*	MICS*	†	SPA, n = 2*	SPA, n = 1*	‡	‡
Okawa et al. 2019b [52]	Adequate contacts with high quality care	Peripartum care	HH*	HH*	†	HH, n = 3*	**†**	‡	‡
Marchant et al. 2015 [34]	High quality contact	Prevention of haemorrhage	HH*	HH*	†	HF (HCW), n = 2*	**†**	‡	‡
Shibanuma et al. 2018 [53]	Continuum of Care achievement	Facility delivery	HH*	HH*	†	HH, n = 2*	**†**	‡	‡
Leslie et al. 2019 [43]	Effective coverage	Delivery care	ENSANUT*	HMIS*	†	†	**†**	‡	HMIS, n = 1*
		Immediate newborn care	ENSANUT*	HMIS*	†	†	†	‡	HMIS, n = 1*
**Care of sick newborns (Table S5 in the ** **Online Supplementary Document** **)**									
Murphy et al. 2018 [45]	Effective coverage	Inpatient neonatal care	Estimate*§	Medical records*	HF, n = 127*	Medical records, n = 3*	Medical records, n = 28*	†	†
**Exclusive breastfeeding (Table S6 in the ** **Online Supplementary Document** **)**									
Nguhiu et al. 2017 [41]	Effective coverage	Exclusive Breastfeeding	DHS*	DHS*	†	†	†	DHS, n = 1*	‡
**Postnatal care (Table S7 in the ** **Online Supplementary Document** **)**									
Baker et al. 2015 [47]	Effective coverage	PPC for women within 48hrs of delivery	HH*	HH*	HF, n = 1*	†	HH, n = 1*	‡	‡
Munos et al. 2018 [39]	Structure-adjusted coverage	Post-discharge PNC for women and baby within 2 d of birth	HH*	HH*	HF, n = 24*	†	†	‡	‡
Okawa et al. 2019a [51]	Quality-adjusted contact	PNC for women and newborn	HH*	HH*	†	HH, n = 6*	HH, n = 11*	‡	‡
Okawa et al. 2019b [52]	Adequate contacts with high quality care	PNC for women and newborn	HH*	HH*	†	HH, n = 4*	HH, n = 10*	‡	‡
Carvajal Aguirre et al 2017 [36]	Content coverage	PNC for women and baby	DHS*	DHS*	†	DHS, n = 3*	DHS, n = 2*	DHS, n = 2*	‡
Shibanuma et al. 2018 [53]	Continuum of Care achievement	PNC for women and child within 48hrs, 2 & 6 wks post-delivery	HH*	HH*	†	HH, n = 1*	HH, n = 2*	‡	‡
Munos et al. 2018 [39]	Process-adjusted coverage	PNC for women and baby within 2 d of birth	HH*	HH*	†	O, n = 3*	O, n = 8*	‡	‡
Marchant et al. 2015 [34]	High quality contact	PPC for women within 48hrs of birth	HH*	HH*	†	†	HH, n = 3*	‡	‡
		PNC for newborn within 48 h of birth	HH*	HH*	†	HH, n = 1*	HH, n = 2*	‡	‡
Hategeka et al. 2020 [54]	Effective coverage	PPC for women before discharge	DHS*	DHS*	†	†	DHS, n = 2*	‡	‡

DHS – Demographic Health Survey, ENSANUT – Mexican National Health and Nutrition Survey, HCW – health care worker interview (conducted as part of a health facility assessment), HF – health facility assessment, HH – household survey, HMIS – Health Management Information System, MICS – Multiple Indicator Cluster Surveys, n – number of items used to measure indicator, O – observations, PPC – post-partum care, PNC – post-natal care, SPA – Service Provision Assessment

*Indicates items measured that map to steps of Marsh et al.’s coverage cascade.

†Indicates no items measured.

‡Indicates steps of the cascade that Marsh et al. consider are not amenable to measurement for a particular service.

§Murphy et al. estimated the target population by extrapolating rate of newborns requiring inpatient services to the total number of live births in the study population [45].

**Table 4:** Mapping effective coverage measures for children under five against the steps of the coverage cascade: by health service or intervention type and the data source and the number of items to measure each step

Reference	Author reported measure	Health service or intervention	Target population	Service contact coverage	Input-adjusted coverage	Intervention coverage	Process quality-adjusted coverage	User-adherence adjusted coverage	Outcome-adjusted coverage
**Sick child care (Table S8 in the ** **Online Supplementary Document** **)**									
Koulidiati et al. 2018 [38]	Effective coverage	Treatment of illness	HH*	HH*	HF, n = 16	†	O, n = 8*	‡	‡
Nguhiu et al. 2017 [41]	Effective coverage	Quality primary care for children: treatment of ARI and/or fever	DHS*	DHS*	SPA, n = 2	†	SPA, n = 5*	‡	‡
Munos et al. 2018 [39]	Structure-adjusted coverage	Care seeking fever, cough, or diarrhoea	MICS*	MICS*	HF, n = 25	†	†	‡	‡
Carter et al. 2018 [55]	Input based effective coverage	Treatment of diarrhoea, fever, ARI or a combination	HH*	HH*	HF, n = 20	†	†	‡	‡
Nguyen et al. 2021 [50]	Input-adjusted coverage	Treatment of diarrhoea or ARI	DHS*	DHS*	SPA, n = 9	†	†	‡	‡
Leslie et al. 2017 [33]	Effective coverage	Treatment for diarrhoea, fever or ARI	DHS/MICS*	DHS/MICS*	†	SPA, n = 2*	SPA, n = 20*	‡	‡
Smith et al. 2010 [56]	Treatment pathway	Treatment for malaria	HH*	HH*	†	HH, n = 2*	HH, n = 1*	‡	‡
Millar et al. 2014 [57]	Treatment pathway	Treatment for malaria	HH*	HH*	†	HH, n = 2*	HH, n = 1*	‡	‡
Hategeka et al. 2020 [54]	Effective coverage	Treatment for diarrhoea	DHS*	DHS*	†	DHS, n = 1*	†	‡	‡
		Treatment for pneumonia	DHS*	DHS*	†	DHS, n = 1*	†	‡	‡
		Treatment for malaria	DHS*	DHS*	†	DHS, n = 1*	†	‡	‡
Munos et al. 2018 [39]	Process-adjusted coverage	Care seeking fever, cough, or diarrhoea	MICS*	MICS*	†	†	O, n = 6*	‡	‡
Leslie et al. 2019 [43]	Effective coverage	Treatment for ARI	ENSANUT*	HMIS*	†	†	†	‡	HMIS, n = 1*
		Treatment for diarrhoea	ENSANUT*	HMIS*	†	†	†	‡	HMIS, n = 1*
**Complementary feeding (Table S9 in the ** **Online Supplementary Document** **)**									
Aaron et al. 2016 [58]	Effective coverage	Complementary feeding supplement	HH*	HH*	†	HH, n = 1*	†	HH, n = 1*	‡
Leyvraz et al. 2016a [59]	Effective coverage	Fortified complementary food	HH*	HH*	†	HH, n = 1*	†	HH, n = 2*	‡
Leyvraz et al. 2016b [60]	Effective coverage	Fortified complementary food	HH*	HH*	†	HH, n = 1*	†	HH, n = 2*	‡
Leyvraz et al. 2018 [61]	Effective coverage	Micronutrient powder	HH*	HH*	†	HH, n = 1*	†	HH, n = 2*	‡
Nguyen et al. 2016 [62]	Effective coverage	Micronutrient powder	HH*	HH*	†	HH, n = 1*	†	HH, n = 2*	‡
**Growth monitoring (Table S10 in the ** **Online Supplementary Document** **)**									
Nguyen et al. 2021 [50]	Input-adjusted coverage	Growth Monitoring	DHS*	DHS*	SPA, n = 6*	†	†	†	†
**ITN (Table S11 in the ** **Online Supplementary Document** **)**									
Nguhiu et al. 2017 [41]	Effective coverage	Use of ITN	DHS*	DHS*	†	DHS, n = 1*	†	‡	‡
**Vaccines (Table S12 in the ** **Online Supplementary Document** **)**									
Nguhiu et al. 2017 [41]	Effective coverage	Quality primary care for children: complete set of basic vaccines	DHS*	DHS*	SPA, n = 2*	†	SPA, n = 5*	‡	‡
Mokdad et al. 2015 [37]	Missed opportunities	MMR vaccine: facilities with MMR in stock	HH*	VC or HH*	HF, n = 1*	†	VC or HH, n = 1*	‡	‡
		MMR vaccine: facilities with MMR stock-out in last 3 mo	HH*	VC or HH*	HF, n = 1*	†	VC or HH, n = 1*	‡	‡
		MMR vaccine: facilities with ORS in stock	HH*	VC or HH*	HF, n = 1*	†	VC or HH, n = 1*	‡	‡
Mmanga et al. 2021 [63]	Effective immunisation coverage	Complete set of basic vaccines	DHS*	DHS*	†	DHS, n = 2*	DHS, n = 1*	‡	‡
Sheff et al. 2020 [64]	Quality coverage	Complete set of basic vaccines	HH*	VC*	†	VC, n = 1*	VC, n = 1*	‡	‡
Mokdad et al. 2015 [37]	Missed opportunities	Timely MMR vaccine	HH*	VC or HH*	†	†	VC or HH, n = 1*	‡	‡

DTP – diphtheria, pertussis, and tetanus, DHS – Demographic Health Survey, ENSANUT – Mexican National Health and Nutrition Survey, HCW – health care worker interview (conducted as part of a health facility assessment), HF – health facility assessment, HH – household survey, HMIS – Health Management Information System, ITN – insecticide treated bednet, MICS – Multiple Indicator Cluster Surveys, MMR – measles, mumps and rubella, n – number of items used to measure indicator, O – observations, ORS – oral rehydration solution, SPA – Service Provision Assessment, VC – vaccination card

*Indicates items measured that map to steps of Marsh et al.’s coverage cascade.

†Indicates no items measured.

‡Indicates steps of the cascade that Marsh et al. consider are not amenable to measurement for a particular service.

**Table 3** and **Table 4** present a summary of the items and data sources used to construct each measure, mapped against the steps of the coverage cascade. Within each table, measures are grouped by intervention or health service. The comprehensive mapping, including full details of the items, is presented in Tables S4 to S12 in the **Online Supplementary Document**. The terminology used by study authors often did not align with the terminology used in the coverage cascade, examples of how we operationalised the cascade given these inconsistencies are presented in **Box 2**. Findings highlight that no standardised effective coverage measure has been used to date in the literature for MNCAHN interventions or services. In the rest of this section we highlight some of the key differences in how studies have defined effective coverage measures.

Box 2 **Operationalising the coverage cascade.**

The dimensions of QoC examined and the terminology used to describe the dimensions of QoC varied between studies (see Table S3 in the **Online Supplementary Document**) and did not typically align with the steps of the proposed coverage cascade.

The input step was the most straightforward to operationalise, although different overarching terms were used across studies including: input indicators [38], facility/service readiness [32,35,37,38,48,49] and structural quality/indicators [38,39,41,45,55].

No studies distinguished between intervention- and process-quality indicators as proposed in the coverage cascade. Instead items related to these two steps were typically captured under a single quality domain. Studies used a range of terms to describe this aspect of QoC including provision of care [46], competent care [51,54], systems competence [54], technical quality [33], process quality/indicators [34,38,39,45], receipt of interventions [36,40,47], signal functions [44] and clinical care processes [42]. In mapping studies against the cascade we classified the individual items measured under the intervention or process-quality step based on definitions presented in **Box 1****.**

### Variation in how services and interventions are defined

Where multiple interventions were being delivered within a single service such as childbirth, postnatal care and sick child care, studies either reported a combined measure or separate measures for each intervention delivered (**Table 3** and **Table 4**). For example, four studies of sick child care reported a single measure for a package of interventions for the management of childhood illness, including diagnosis and treatment of malaria, treatment of diarrhoea with oral rehydration solution and treatment of respiratory infections [33,38,39,50,55]. Conversely, two studies on sick child care presented a separate measure for each intervention examined [43,54].

### Variation in the number and type of steps of the coverage cascade adjusted for

The cascade specifies five steps that contact (or crude) coverage should be adjusted for to estimate effective coverage: inputs, interventions, process-quality, user-adherence and outcomes (**Figure 1**). **Table 3** and **Table 4** present a summary of the mapping of the individual items measured in each study against the steps of the cascade. Details of the specific items measured are presented in Tables S4 to S12 in the **Online Supplementary Document**.

In mapping the items from the studies against the coverage cascade, we identified only three studies (two examining childbirth and newborn care, and one neonatal care) that measured items related to all recommended steps of the cascade [44-46]. It can be seen in **Table 3** and **Table 4** that only one study, conducted in Mexico, that aimed to estimate effective coverage of delivery and newborn care and care for children under five with diarrhoea and respiratory illness using administrative data (IMSS), adjusted contact coverage for health outcomes [43].

Just under half of the measures adjusted contact coverage for items from only one of the five cascade steps (31 out of 64 measures); the maximum number of cascade steps captured in a single adjusted measure was three (4 out of 64 measures) [36,44-46]. The steps of the coverage cascade most commonly adjusted for varied by intervention or health service. For childbirth and immediate newborn care, the most common adjustment was for items related to the input step (15 out of 24 measures). For postnatal care, most measures were adjusted for items related to the process quality step (9 out of 10 measures). All complementary feeding measures adjusted for items related to intervention and user-adherence steps (5 out of 5 measures). For sick child care the most common adjustments were for items related to the intervention and process quality steps (6 out of 14 measures). All vaccine measures were adjusted for the process quality cascade step (7 out of 7 measures).

Inputs were measured using health facility data. Items classified under intervention and process quality steps were estimated using a range of data sources. Sick child care, postnatal care and complementary feeding primarily derived data from women/caregivers’ recall in household surveys, while childbirth and immediate newborn care most frequently used health care workers’ reports of their actions taken in health facility assessments. Direct observations of care were only used in eight measures across six studies: two childbirth and newborn care [40,42], one postnatal care [39], four sick child care [33,38,39,41] and one vaccine [41]. Nguhiu et al. 2017 adjusted their measures of care seeking for acute respiratory infection and/or fever and routine vaccination for the same “quality of primary care for children” measure (consisting of seven items across the input and process cascade steps) [41]. The receipt and timing of vaccination were based on vaccination cards, with caregivers’ recall used when vaccine cards were not available.

### Variation in the definitions of individual steps of the cascade and approach to generating a composite score

Studies varied in their approach to constructing measures. While some selected tracer items, others defined more comprehensive, composite, measures. For example, the total number of items used to measure inputs ranged from one to 127 [37,45,47]. Mapping items against the coverage cascade demonstrated that there was little consistency in the items used within different interventions or health services. For example, inputs can be broadly classified into four areas: 1) facility infrastructure, 2) staff, training and guidelines, 3) availability of supplies, commodities and equipment, and 4) service availability. Nine of the 15 childbirth and immediate newborn care measures that included inputs measured items related to facility infrastructure [32,35,39,41,42,44,46,48,49]. In total 13 different items were examined, ranging from two to eight items in a single measure [48,49]; none of the 13 items were common to all measures. Further, individual items were defined in different ways primarily based on the data source (see Table S4 in the **Online Supplementary Document**).

Items used to assess process quality of care were skewed towards provision of care. Only two measures, which both examined childbirth and immediate newborn care, included items related to patient experience or respectful care [42,44].

The justification for how items were selected was not always well described. Only 23 studies reported the approach taken, which varied across service or intervention type. International recommendations were most frequently cited as guiding item selection in studies of childbirth and sick child care [32-35,39,48,50,51,53,54,56,57,62], while national guidelines were reported to inform timing and completeness of vaccinations [37,63,64]. Differences in national priorities account for some of the variation in the items selected, for example two studies, one in Kenya and the other in Ghana, included different packages of vaccines based on the respective national guidelines [41,64]. Several studies reported that selection was based on previous literature [32,35,38,42,44,49,51,53,62]. Two studies reported that selection was in part informed in consultation with local clinicians and health administrators at the study site [44,53]. Item selection was also reported to be influenced by data availability; one study examining change over time noted that item selection was restricted based on item availability across different data sets [54].

Studies have taken different approaches to generating a summary measure for QoC, including generating an average score, a binary score (based on all items being present or based on a threshold) and a categorical score. For example, taking studies of childbirth, Wang et al. (2019) calculated facility readiness to provide delivery care as the average number of items available standardised out of 100 [35]; Willey et al. (2018) classified facilities as ‘ready’ if they had all commodities measured available [49]; Kanyangara et al. (2018) on the other hand classified facilities as ready to provide obstetric services if they had 20 or more of the 23 items measured available [32]. Kanyangara et al. also assessed availability of obstetric services in health facilities and classified facilities into four levels of functionality based on the number and type of signal functions performed: 1) comprehensive emergency obstetric care(CEmOC), 2) basic emergency obstetric care (BEmOC), 3) basic emergency obstetric care-2 (BEmOC-2), and 4) low/substandard.

Studies also took a different approach to generating an overall effective coverage measure. The majority presented a composite measure that adjusted contact coverage for all items measured (see Table S3 in the **Online Supplementary Document**) [33-36,38,40,41,44,45,49-55]. Three studies presented separate measures adjusted for different components of QoC [32,39,42]. For example, Munos et al. presented two adjusted measures for each intervention examined, one adjusting contact coverage for structural quality and the second adjusting for process quality. The remaining studies presented effective coverage as a cascade [37,43,46,47,56-64]. While there was some consistency in approach between interventions, notably studies examining complementary feeding and malaria, there was no standard approach across studies.

## DISCUSSION

Previous reviews have demonstrated that measuring contact with a health service is not sufficient to indicate the potential for lives saved from proven interventions [7,13]. As a result, adjusting contact coverage measures for QoC has become a priority goal in global health measurement. Global consensus has now coalesced around coverage cascades as a useful tool for assessing performance along the sequences of interactions between the population in need and the health system, and in identifying where bottlenecks in service provision have occurred [7,22]. By mapping existing research against the proposed cascade (**Figure 1**), this review demonstrates that there is poor alignment between the effective coverage measures applied in previous studies and the proposed cascade measurement approach. This finding suggests the need for increased dissemination of the proposed casecade approach to promote greater uptake.

We examined the dimensions of QoC that have been used to adjust population-level contact coverage measures and how the items used to construct the measures relate to the steps of the coverage cascade outlined by the Effective Coverage Think Tank Group. We found limited consistency in the definition and construction of effective coverage measures for preventative and curative services and interventions from childbirth through to children up to nine years old in LMICs. An exception was the five studies which examined provision of micronutrient powders or complementary foods; these studies conceptualised effective coverage based on the same four steps (message coverage, contact coverage, partial coverage and effective coverage) defined using similar items collected through household surveys [58-62]. The uniformity in approach is likely due to being undertaken by the same group of authors.

Mapping the measures against the coverage cascade we identified three key areas of divergence: i) different approaches to combining individual interventions when a study examined a service package; ii) adjustments to different steps of the coverage cascade for the same health services or interventions; and iii) different approaches to defining and constructing the QoC measure. These differences limit comparability of effective coverage measures over time and place, and thus the ability to use these measures to track progress at national and global levels.

Effective coverage measures have been generated for single interventions or several interventions combined, reducing comparability across measures of similar interventions or health services. These differences may be driven by the focus of the study, which, in turn, may have been guided by national priorities and data availability [13].

The type and number of adjustments made to contact coverage measures also varied. The majority of studies adjusted contact coverage for one step; only three measures adjusted for all three steps described in the Effective Think Tank Group coverage cascade to generate a quality-adjusted measure (inputs, interventions and process quality) [44-46]. The choice of adjustment is likely to be driven by data availability, the intervention type or country priorities. However, even where studies had relevant data available they did not always make adjustments for all cascade steps. For example, the SPA includes a facility inventory module and in some countries additional modules on health worker interview, direct observation of care and patient exit interviews. Two studies (one childbirth and one sick child care) used SPA data to adjust for interventions and process quality steps but did not adjust for inputs [33,40].

Approaches taken to construct the individual adjusted coverage measures were highly variable, both in terms of the number of items used and the methods for generating a summary measure. This in part reflects wider challenges associated with measurement of QoC. Quality is a complex construct that represents multiple dimensions with few standardised and validated measures [65]. Two studies that defined thresholds for minimum quality both commented that thresholds have not been empirically defined and consequently the cut offs selected were somewhat arbitrary [38,46].

Data availability has considerable implications for the feasibility of constructing coverage cascades. Of the five steps beyond contact coverage, adjustment for the process quality step was the most common, based on respondent’s self-reports in household surveys. Adjustment for inputs, on the other hand, was restricted to interventions delivered at a facility and only feasible where studies also included a health facility assessment. Reports from health facility assessments such as the SPA and SARA are not available in all countries, for example, Nigeria has no SPA or SARA data despite having one of the highest maternal and child mortality rates globally [66,67]. Further, nationally representative facility surveys are only conducted periodically and are often not coordinated with other household surveys. A study conducted in Rwanda used four rounds of DHS between 2000 and 2015, the authors noted they did not include SPA data as it was conducted in 2006 only [54]. The health facility assessments themselves have limitations as in the case of the standard SPA protocol direct observations of care are only collected for three services (antenatal care, family planning and sick child care) [68]. This review identified limited evidence of the use of routine data. Only one study conducted in Mexico used routine health information systems to estimate quality of services received [43]. That study adjusted for health outcomes (adverse outcomes or mortality) only and was the only study included to do so.

Each of these areas of heterogeneity in definition and construction of QoC influenced the effective coverage estimates. Heterogeneity is not limited to the issues identified in this review: a recent review of methodological considerations for linking household and health facility data also identified a lack of standardisation in approaches to linking [69].

### Limitations of the evidence

No studies were identified among children aged five to nine years, reflecting the lack of data available to measure coverage of interventions for this age-group [70]; and only one study examined curative interventions among sick neonates, again reflecting a lack of data but also indicative of the measurement challenges inherent for emergency care for this population group [9,71,72].

All studies identified were undertaken for research purposes and there was limited evidence of whether and how these measures were used by decision makers. Studies that link health facility assessments and population-based surveys to calculate effective coverage require complex linking methods and may not be feasible for routine analyses, outside of research purposes. One study reported that the Ministry of Health in Vietnam updated regulations based on the findings, but did not report whether or how the government engaged with the effective coverage measure [62].

### Limitations of approach

The term “effective coverage” is not widely used in the literature, and while we attempted to ensure search terms were as comprehensive as possible by expanding on two previous reviews [7,13], it is likely that relevant studies that have conceptualised quality-adjustment in a different way may not have been identified. For example, two studies examining treatment of malaria used the term “treatment pathway” [56,57]. In the field of HIV researchers have developed similar concepts, namely treatment cascades and prevention cascades [73]. The challenges in searching for relevant literature highlights the complexity of this field and the need for greater standardisation in terminology. Further, additional relevant studies may have been missed as search terms and literature were restricted to English and we did not systematically search for any grey literature, although we consulted with members of CHAT for any additional documents to include in the review. Several authors were members of the Effective Coverage Think Tank group so we did not think a systematic search of the grey literature would yield a significant number of studies that we had not already included in the review. The scope of the study was limited to interventions from childbirth to children up to nine years of age, and as such does not capture interventions across the whole continuum of MNCAHN care.

The mapping of items against the coverage cascade highlighted a lack of clarity in the definitions of the individual cascade steps. In the Effective Coverage Think Tank cascade, the inclusion of ‘intervention’ as a distinct step from service contact was not in line with much of the literature which most frequently use intervention coverage to refer to a crude coverage measure. Likewise, the use of the term ‘quality’ as a standalone step in the cascade is confusing given the wider conceptualisation of quality as a multi-dimensional concept [15]. We found the distinction between the intervention and quality steps of the cascade was not clear cut; we differentiated between these two steps during data extraction based on whether the intervention delivered resulted in a direct health benefit or whether it enhanced the interaction. In most cases, studies that collected data falling under these two steps referred to items as ‘process indicators’; items we have mapped under these two steps might therefore be misclassified.

### Next steps

The cascade should be refined to address the problems noted above on the use of the terms “intervention” and “quality”. If effective coverage measures are to have greater utility in tracking progress and driving change in countries, then further work is needed to implement the coverage cascade approach and harmonised methods for measuring each step of the cascade. In the short term, there is a need for greater transparency and more specificity in the reporting of effective coverage measures. Future studies should provide more information on how the effective coverage metrics were constructed, including identifying the items and methods used to construct measures and the rationale for those choices. In the longer term, there is a need for greater harmonisation and consensus on standard indicators, which requires global guidance on best practice. The full mapping of the items against the coverage cascade, presented in Tables S4 to S12 in the **Online Supplementary Document**, provides a useful starting point for future research and research guidance.

Second, while there have been shifts to generating coverage measures that have adjusted for quality, as seen in the latest DHS data – which, for example, added questions on the content of PNC for women - and efforts by the Mexican Ministry of Health – which has been measuring effective coverage for skilled birth attendance, services delivered to premature babies and treatment of acute respiratory infections in children to benchmark perfomance across States, they are not yet widely-used [74]. To maximise the utility of effective coverage measures there is a need to explore their relevance for country decision makers so that measures are actionable, responsive to country needs, and interpretable. Finally, further research is needed to understand and improve the feasibility of measuring all steps of the cascade, including assessing the availability of relevant data and the potential for using routine data sources.

## CONCLUSIONS

This is the first review to specifically examine the definitions and measurement of quality adjustments made to contact coverage measures of life-saving interventions from childbirth through to childhood to the age of nine and to map these against the coverage cascade proposed by the Effective Coverage Think Tank Group. The lack of any study on children aged five to nine years indicates the need for greater focus and visibility for this population group. The findings highlight substantial heterogeneity in both definitions of and measurement approaches for QoC, limiting the comparability of effective coverage measures. They further demonstrate that a major shift in measurement approach will be required if the coverage cascade is to be adopted. There is a need for greater standardisation of terminology and transparency to understand how effective coverage measures are defined and the rationale for the measurement approach taken. Such progress will improve comparability for global monitoring and facilitate uptake by governments for tracking progress and targeting investments in life-saving health policies and programmes.

## Additional material


Online Supplementary Document

## References

[1] World Health Organization. SDG 3: Ensure healthy lives and promote wellbeing for all at all ages. Available: https://wwwwhoint/sdg/targets/en/. Accessed: 12 July 2020.

[2] WHO, UNICEF, UNFPA, World Bank Group and the United Nations Population Division. Maternal mortality: Levels and trends. 2000 to 2017. Geneva: World Health Organization. 2019.

[3] Kruk ME Gage AD Arsenault C Jordan K Leslie HH Roder-DeWan S High-quality health systems in the Sustainable Development Goals era: time for a revolution. Lancet Glob Health. 2018;6:e1196. 10.1016/S2214-109X(18)30386-3 30196093 PMC7734391

[4] Moller AB Patten JH Hanson C Morgan A Say L Diaz T Monitoring maternal and newborn health outcomes globally: a brief history of key events and initiatives. Trop Med Int Health. 2019;24:1342-68. 10.1111/tmi.13313 31622524 PMC6916345

[5] Moran AC Moller AB Chou D Morgan A El Arifeen S Hanson C ‘What gets measured gets managed’: revisiting the indicators for maternal and newborn health programmes. Reprod Health. 2018;15:19. 10.1186/s12978-018-0465-z 29394947 PMC5797384

[6] Boerma T Requejo J Victora CG Amouzou A George A Agyepong I Countdown to 2030: tracking progress towards universal coverage for reproductive, maternal, newborn, and child health. Lancet. 2018;391:1538-48. 10.1016/S0140-6736(18)30104-1 29395268

[7] Amouzou A Leslie HH Ram M Fox M Jiwani SS Requejo J Advances in the measurement of coverage for RMNCH and nutrition: from contact to effective coverage. BMJ Glob Health. 2019;4:e001297. 10.1136/bmjgh-2018-001297 31297252 PMC6590972

[8] Grove J Claeson M Bryce J Amouzou A Boerma T Waiswa P Maternal, newborn, and child health and the Sustainable Development Goals–a call for sustained and improved measurement. Lancet. 2015;386:1511-4. 10.1016/S0140-6736(15)00517-6 26530604 PMC7613198

[9] Marchant T Bryce J Victora C Moran AC Claeson M Requejo J Improved measurement for mothers, newborns and children in the era of the Sustainable Development Goals. J Glob Health. 2016;6:010506. 10.7189/jogh.06.010506 27418960 PMC4938381

[10] Requejo JH Newby H Bryce J Measuring coverage in MNCH: challenges and opportunities in the selection of coverage indicators for global monitoring. PLoS Med. 2013;10:e1001416. 10.1371/journal.pmed.1001416 23667336 PMC3646210

[11] Murray CJL, Evans DB. Technical Consultation on Effective Coverage in Health Systems. Health systems performance assessment: debates, methods and empiricism. Geneva: World Health Organization; 2003. p. 125-34.

[12] Ng M Fullman N Dieleman JL Flaxman AD Murray CJL Lim SS Effective Coverage: A Metric for Monitoring Universal Health Coverage. PLoS Med. 2014;11:e1001730. 10.1371/journal.pmed.1001730 25243780 PMC4171091

[13] Jannati A Sadeghi V Imani A Saadati M Effective coverage as a new approach to health system performance assessment: a scoping review. BMC Health Serv Res. 2018;18:886. 10.1186/s12913-018-3692-7 30470214 PMC6251131

[14] Shengelia B Tandon A Adams OB Murray CJL Access, utilization, quality, and effective coverage: An integrated conceptual framework and measurement strategy. Soc Sci Med. 2005;61:97-109. 10.1016/j.socscimed.2004.11.055 15847965

[15] Donabedian A The quality of care. How can it be assessed? JAMA. 1988;260:1743-8. 10.1001/jama.1988.03410120089033 3045356

[16] Tunçalp Ӧ Were W MacLennan C Oladapo O Gülmezoglu A Bahl R Quality of care for pregnant women and newborns—the WHO vision. BJOG. 2015;122:1045-9. 10.1111/1471-0528.13451 25929823 PMC5029576

[17] WHO. Standards for improving quality of maternal and newborn care in health facilities. Geneva: World Health Organization. 2016.

[18] Tanahashi T Health service coverage and its evaluation. Bull World Health Organ. 1978;56:295-303. 96953 PMC2395571

[19] WHO. Background paper for the Technical Consultation on Effective Coverage of Health Systems, 27-29 August 2001, Rio de Janeiro, Brazil. Available: http://citeseerxistpsuedu/viewdoc/download?rep=rep1&type=pdf&doi=10111111239. Accessed: 31 May 2020.

[20] WHO, The World Bank. Tracking Universal Health Coverage. First Global Monitoring Report. Geneva: World Health Organization. 2015.

[21] GBD 2019 Universal Health Coverage Collaborators. Measuring universal health coverage based on an index of effective coverage of health services in 204 countries and territories, 1990-2019: a systematic analysis for the Global Burden of Disease Study 2019. Lancet. 2020;396:1250-84. 10.1016/S0140-6736(20)30750-9 32861314 PMC7562819

[22] Marsh AD Muzigaba M Diaz T Requejo J Jackson D Chou D Effective coverage measurement in maternal, newborn, child, and adolescent health and nutrition: progress, future prospects, and implications for quality health systems. Lancet Glob Health. 2020;8:e730-6. 10.1016/S2214-109X(20)30104-2 32353320 PMC7196884

[23] Cumpston M Li T Page MJ Chandler J Welch VA Higgins JP Updated guidance for trusted systematic reviews: a new edition of the Cochrane Handbook for Systematic Reviews of Interventions. Cochrane Database Syst Rev. 2019;10:ED000142. 10.1002/14651858.ED000142 31643080 PMC10284251

[24] Grant MJ Booth A A typology of reviews: an analysis of 14 review types and associated methodologies. Health Info Libr J. 2009;26:91-108. 10.1111/j.1471-1842.2009.00848.x 19490148

[25] Page MJ McKenzie JE Bossuyt PM Boutron I Hoffmann TC Mulrow CD The PRISMA 2020 statement: an updated guideline for reporting systematic reviews. BMJ. 2021;372. 33782057 10.1136/bmj.n71 PMC8005924

[26] Tricco AC, Langlois E, Straus SE. Rapid reviews to strengthen health policy and systems: a practical guide. Geneva: World Health Organization. 2017.

[27] The World Bank. World Bank Country and Lending Groups: Country Classifications. Available: https://datahelpdesk.worldbank.org/knowledgebase/articles/906519-world-bank-country-and-lending-groups Accessed: 1 November 2021.

[28] Child Health Accountability Tracking Technical Advisory Group (CHAT) website. Available: https://www.who.int/data/maternal-newborn-child-adolescent-ageing/advisory-groups/chat. Accessed: 22 August 2021.

[29] Mother and Newborn Information for Tracking Outcomes and Results (MoNITOR) website. Available: https://wwwwhoint/data/maternal-newborn-child-adolescent-ageing/advisory-groups/monitor Accessed: 3 September 2021.

[30] PMNCH, Aga Khan University. Essential Interventions, Commodities and Guidelines for Reproductive, Maternal, Newborn and Child Health. A global review of the key interventions related to reproductive, maternal, newborn and child health. Geneva: Partnership for Maternal Health, Newborn and Child Health. 2011.

[31] The Endnote team. EndNote. 9th ed. Philadelphia, PA: Clarivate Analytics. 2013.

[32] Kanyangarara M Chou VB Creanga AA Walker N Linking household and health facility surveys to assess obstetric service availability, readiness and coverage: evidence from 17 low- and middle-income countries. J Glob Health. 2018;8:010603. 10.7189/jogh.08.010603 29862026 PMC5963736

[33] Leslie HH Malata A Ndiaye Y Kruk ME Effective coverage of primary care services in eight high-mortality countries. BMJ Glob Health. 2017;2:e000424. 10.1136/bmjgh-2017-000424 29632704 PMC5887868

[34] Marchant T Tilley-Gyado RD Tessema T Singh K Gautham M Umar N Adding content to contacts: measurement of high quality contacts for maternal and newborn health in Ethiopia, north east Nigeria, and Uttar Pradesh, India. PLoS One. 2015;10:e0126840. 10.1371/journal.pone.0126840 26000829 PMC4441429

[35] Wang W Mallick L Allen C Pullum T Effective coverage of facility delivery in Bangladesh, Haiti, Malawi, Nepal, Senegal, and Tanzania. PLoS One. 2019;14:e0217853. 10.1371/journal.pone.0217853 31185020 PMC6559642

[36] Carvajal-Aguirre L Amouzou A Mehra V Ziqi M Zaka N Newby H Gap between contact and content in maternal and newborn care: An analysis of data from 20 countries in sub-Saharan Africa. J Glob Health. 2017;7:020501. 10.7189/jogh.07.020501 29423178 PMC5804037

[37] Mokdad AH Gagnier MC Colson KE Dansereau E Zúñiga-Brenes P Ríos-Zertuche D Missed Opportunities for Measles, Mumps, and Rubella (MMR) Immunization in Mesoamerica: Potential Impact on Coverage and Days at Risk. PLoS One. 2015;10:e0139680. 10.1371/journal.pone.0139680 26506563 PMC4624243

[38] Koulidiati JL Nesbitt RC Ouedraogo N Hien H Robyn PJ Compaoré P Measuring effective coverage of curative child health services in rural Burkina Faso: a cross-sectional study. BMJ Open. 2018;8:e020423. 10.1136/bmjopen-2017-020423 29858415 PMC5988102

[39] Munos MK Maiga A Do M Sika GL Carter ED Mosso R Linking household survey and health facility data for effective coverage measures: a comparison of ecological and individual linking methods using the Multiple Indicator Cluster Survey in Côte d’Ivoire. J Glob Health. 2018;8:020803. 10.7189/jogh.08.020803 30410743 PMC6211616

[40] Joseph NT Piwoz E Lee D Malata A Leslie HH Examining coverage, content, and impact of maternal nutrition interventions: the case for quality-adjusted coverage measurement. J Glob Health. 2020;10:010501. 10.7189/jogh.10.010501 32082545 PMC7020656

[41] Nguhiu PK Barasa EW Chuma J Determining the effective coverage of maternal and child health services in Kenya, using demographic and health survey data sets: tracking progress towards universal health coverage. Trop Med Int Health. 2017;22:442-53. 10.1111/tmi.12841 28094465 PMC5396138

[42] Sharma J Leslie HH Kundu F Kruk ME Poor Quality for Poor Women? Inequities in the Quality of Antenatal and Delivery Care in Kenya. PLoS One. 2017;12:e0171236. 10.1371/journal.pone.0171236 28141840 PMC5283741

[43] Leslie HH Doubova SV Pérez-Cuevas R Assessing health system performance: effective coverage at the Mexican Institute of Social Security. Health Policy Plan. 2019;34:ii67-76. 10.1093/heapol/czz105 31723962

[44] Nesbitt RC Lohela TJ Manu A Vesel L Okyere E Edmond K Quality along the continuum: a health facility assessment of intrapartum and postnatal care in Ghana. PLoS One. 2013;8:e81089. 10.1371/journal.pone.0081089 24312265 PMC3842335

[45] Murphy GAV Gathara D Mwachiro J Abuya N Aluvaala J English M Effective coverage of essential inpatient care for small and sick newborns in a high mortality urban setting: a cross-sectional study in Nairobi City County, Kenya. BMC Med. 2018;16:72. 10.1186/s12916-018-1056-0 29783977 PMC5963150

[46] Larson E Vail D Mbaruku GM Mbatia R Kruk ME Beyond utilization: measuring effective coverage of obstetric care along the quality cascade. Int J Qual Health Care. 2017;29:104-10. 27920246 10.1093/intqhc/mzw141 PMC5890864

[47] Baker U Peterson S Marchant T Mbaruku G Temu S Manzi F Identifying implementation bottlenecks for maternal and newborn health interventions in rural districts of the United Republic of Tanzania. Bull World Health Organ. 2015;93:380-9. 10.2471/BLT.14.141879 26240459 PMC4450702

[48] Kemp CG Sorensen R Puttkammer N Grand’Pierre R Honoré JG Lipira L Health facility readiness and facility-based birth in Haiti: a maximum likelihood approach to linking household and facility data. J Glob Health Rep. 2018;2:e2018023. 10.29392/joghr.2.e2018023 31406933 PMC6690361

[49] Willey B Waiswa P Kajjo D Munos M Akuze J Allen E Linking data sources for measurement of effective coverage in maternal and newborn health: what do we learn from individual- vs ecological-linking methods? J Glob Health. 2018;8:010601. 10.7189/jogh.06.0207028.010601 29497508 PMC5823029

[50] Nguyen PH Khương LQ Pramanik P Billah SM Menon P Piwoz E Effective coverage of nutrition interventions across the continuum of care in Bangladesh: insights from nationwide cross-sectional household and health facility surveys. BMJ Open. 2021;11:e040109. 10.1136/bmjopen-2020-040109 33472778 PMC7818835

[51] Okawa S Win HH Leslie HH Nanishi K Shibanuma A Aye PP Quality gap in maternal and newborn healthcare: a cross-sectional study in Myanmar. BMJ Glob Health. 2019;4:e001078. 10.1136/bmjgh-2018-001078 30997160 PMC6441248

[52] Okawa S Gyapong M Leslie H Shibanuma A Kikuchi K Yeji F Effect of continuum-of-care intervention package on improving contacts and quality of maternal and newborn healthcare in Ghana: a cluster randomised controlled trial. BMJ Open. 2019;9:e025347. 31511278 10.1136/bmjopen-2018-025347 PMC6738678

[53] Shibanuma A Yeji F Okawa S Mahama E Kikuchi K Narh C The coverage of continuum of care in maternal, newborn and child health: a cross-sectional study of woman-child pairs in Ghana. BMJ Glob Health. 2018;3:e000786. 10.1136/bmjgh-2018-000786 30233827 PMC6135430

[54] Hategeka C Arsenault C Kruk ME Temporal trends in coverage, quality and equity of maternal and child health services in Rwanda, 2000-2015. BMJ Glob Health. 2020;5:e002768. 10.1136/bmjgh-2020-002768 33187962 PMC7668303

[55] Carter ED Ndhlovu M Eisele TP Nkhama E Katz J Munos M Evaluation of methods for linking household and health care provider data to estimate effective coverage of management of child illness: results of a pilot study in Southern Province, Zambia. J Glob Health. 2018;8:010607. 10.7189/jogh.08.010607 29983929 PMC6013179

[56] Smith LA Bruce J Gueye L Helou A Diallo R Gueye B From fever to anti-malarial: the treatment-seeking process in rural Senegal. Malar J. 2010;9:333. 10.1186/1475-2875-9-333 21092176 PMC3000420

[57] Millar KR McCutcheon J Coakley EH Brieger W Ibrahim MA Mohammed Z Patterns and predictors of malaria care-seeking, diagnostic testing, and artemisinin-based combination therapy for children under five with fever in Northern Nigeria: a cross-sectional study. Malar J. 2014;13:447. 10.1186/1475-2875-13-447 25413231 PMC4253990

[58] Aaron GJ Strutt N Boateng NA Guevarra E Siling K Norris A Assessing Program Coverage of Two Approaches to Distributing a Complementary Feeding Supplement to Infants and Young Children in Ghana. PLoS One. 2016;11:e0162462. 10.1371/journal.pone.0162462 27755554 PMC5068796

[59] Leyvraz M Rohner F Konan AG Esso LJ Woodruff BA Norte A High Awareness but Low Coverage of a Locally Produced Fortified Complementary Food in Abidjan, Côte d’Ivoire: Findings from a Cross-Sectional Survey. PLoS One. 2016;11:e0166295. 10.1371/journal.pone.0166295 27824917 PMC5100976

[60] Leyvraz M Wirth JP Woodruff BA Sankar R Sodani PR Sharma ND High Coverage and Utilization of Fortified Take-Home Rations among Children 6-35 Months of Age Provided through the Integrated Child Development Services Program: Findings from a Cross-Sectional Survey in Telangana, India. PLoS One. 2016;11:e0160814. 10.1371/journal.pone.0160814 27695118 PMC5047467

[61] Leyvraz M David-Kigaru DM Macharia-Mutie C Aaron GJ Roefs M Tumilowicz A Coverage and Consumption of Micronutrient Powders, Fortified Staples, and Iodized Salt Among Children Aged 6 to 23 Months in Selected Neighborhoods of Nairobi County, Kenya. Food Nutr Bull. 2018;39:107-15. 10.1177/0379572117737678 29284306

[62] Nguyen M Poonawala A Leyvraz M Berger J Schofield D Nga TT A Delivery Model for Home Fortification of Complementary Foods with Micronutrient Powders: Innovation in the Context of Vietnamese Health System Strengthening. Nutrients. 2016;8:259. 10.3390/nu8050259 27136585 PMC4882672

[63] Mmanga K Mwenyenkulu TE Nkoka O Ntenda PAM Tracking immunization coverage, dropout and equity gaps among children ages 12-23 months in Malawi - bottleneck analysis of the Malawi Demographic and Health Survey. Int Health. 2021 Jun 21;ihab038. 10.1093/inthealth/ihab038 34153106 PMC9070459

[64] Sheff MC Bawah AA Asuming PO Kyei P Kushitor M Phillips JF Evaluating health service coverage in Ghana’s Volta Region using a modified Tanahashi model. Glob Health Action. 2020;13:1732664. 10.1080/16549716.2020.1732664 32174254 PMC7144185

[65] Hanefeld J Powell-Jackson T Balabanova D Understanding and measuring quality of care: dealing with complexity. Bull World Health Organ. 2017;95:368-74. 10.2471/BLT.16.179309 28479638 PMC5418826

[66] WHO. The Global Health Observatory: Under-five mortality rate (probability of dying by age 5 per 1000 live births). Available: https://wwwwhoint/data/gho/data/indicators/indicator-details/GHO/under-five-mortality-rate-(probability-of-dying-by-age-5-per-1000-live-births). Accessed: 11 June 2021.

[67] WHO. The Global Health Observatory: Maternal mortality ratio (per 100 000 live births). Available: https://wwwwhoint/data/gho/data/indicators/indicator-details/GHO/maternal-mortality-ratio-(per-100-000-live-births). Accessed: 11 June 2021.

[68] Sheffel A Karp C Creanga AA Use of Service Provision Assessments and Service Availability and Readiness Assessments for monitoring quality of maternal and newborn health services in low-income and middle-income countries. BMJ Glob Health. 2018;3:e001011. 10.1136/bmjgh-2018-001011 30555726 PMC6267320

[69] Carter ED Leslie HH Marchant T Amouzou A Munos MK Methodological considerations for linking household and healthcare provider data for estimating effective coverage: a systematic review. BMJ Open. 2021;11:e045704. 10.1136/bmjopen-2020-045704 34446481 PMC8395298

[70] Requejo J Diaz T Park L Chou D Choudhury A Guthold R Assessing coverage of interventions for reproductive, maternal, newborn, child, and adolescent health and nutrition. BMJ. 2020;368:l6915. 10.1136/bmj.l6915 31983681 PMC7461908

[71] Moller A-B Newby H Hanson C Morgan A El Arifeen S Chou D Measures matter: A scoping review of maternal and newborn indicators. PLoS One. 2018;13:e0204763. 10.1371/journal.pone.0204763 30300361 PMC6177145

[72] Moxon SG Ruysen H Kerber KJ Amouzou A Fournier S Grove J Count every newborn; a measurement improvement roadmap for coverage data. BMC Pregnancy Childbirth. 2015;15 Suppl 2:S8. 10.1186/1471-2393-15-S2-S8 26391444 PMC4577758

[73] Hargreaves J Auerback J Hensen B Gregson S Strengthening primary HIV prevention: better use of data to improve programmes, develop strategies and evaluate progress. J Int AIDS Soc. 2020;23(Suppl 3):e25538. 10.1002/jia2.25538 32602656 PMC7325501

[74] Lozano R Soliz P Gakidou E Abbott-Klafter J Feehan DM Vidal C Benchmarking of performance of Mexican states with effective coverage. Lancet. 2006;368:1729-41. 10.1016/S0140-6736(06)69566-4 17098091

