# Supplementary material for: A rapid systematic review and evidence synthesis of effective coverage measures and cascades for childbirth, newborn and child health in low- and middle-income countries
Source: J Glob Health. 2022 Jan 15;12:04001. doi: 10.7189/jogh.12.04001 (PMC8801924; doi:10.7189/jogh.12.04001)
Supplement: Online Supplementary Document [file jogh-12-04001-s001.pdf]

APPENDIX S1 – SEARCH TERMS

Table S1 Summary of search terms

Term	Concept	Search terms
1	Coverage	Clinical competence.mp.; OR Contact.mp.; Content coverage.mp.; OR Coverage.mp.; effective coverage.mp.; OR Health Facilities.mp.; OR Health Services Accessibility.mp.; OR Health Services Needs and Demand.mp.; OR high quality contact.mp.; OR input adjusted coverage.mp.; OR intervention coverage.mp.; OR Medical Audit.mp.; OR outcome adjusted coverage.mp.; OR Patient Acceptance of Health Care.mp.; OR Population level coverage.mp.; quality adjusted contact.mp.; OR quality adjusted coverage.mp.; OR quality adjusted measurement.mp.; OR quality along the continuum.mp.; OR Quality Assurance.mp.; OR quality cascade.mp.; OR quality contact.mp.; OR Quality improvement.mp.; OR Quality indicators.mp.; OR OR Quality of care.mp.; OR Quality of Health Care.mp.;
2	Bottleneck	adequate care.mp.; OR bottleneck.mp.; OR bottleneck analysis.mp.; OR bottleneck of implementation.mp.; OR gaps in coverage.mp.; OR implementation bottleneck.mp.; OR Tanahashi.mp.
3	Linking	linking household.mp.; OR linking household.mp. OR linking household survey and health facility.mp.; OR linking service.mp.
**4**	**Effective coverage**	**Term 1 OR Term 2 OR Term 3**
5	Child health intervention	care seeking.mp. ; OR childhood illness.mp. ; OR pneumonia.mp. ; OR diarrh*.mp. ; OR newborn illness.mp. ; OR health worker.mp. ; OR facility readiness.mp. ; OR intervention.mp. ; OR utilization.mp. ; OR access to care.mp. ; OR availability coverage.mp. ; OR health facilities.mp. ; OR accessibility coverage.mp. ; OR health facility.mp. ; OR HIV.mp. ; OR perinatal care.mp. ; OR postnatal care.mp. ; OR immunization.mp. ; OR immunisation.mp. ; OR treatment of sick children.mp. ; OR nutrition coverage.mp. ; OR newborn care.mp. ; OR breastfeeding.mp. ; OR infant feeding.mp. ; OR (maternal and child health intervention).mp. ; OR MCH intervention.mp. ; OR content intervention.mp. ; OR childhood immunization.mp. ; OR childhood vaccination.mp. ; OR missed opportunities.mp. ; OR malaria prevention.mp. ; OR malaria treatment.mp. ; OR health service delivery.mp. ; OR health service provision.mp. ; OR community health worker.mp. ; OR CHW.mp.
6	Newborn/child	Adolescent.mp.; OR baby.mp.; OR boy.mp.; OR child mortality.mp.; OR child*.mp.; OR child, preschool.mp.; OR exp adolescent/ or exp child/ or exp infant/; OR girl.mp.; OR infant.mp.; OR infant.mp., low birth weight.mp.; OR infant, newborn.mp.; OR infant, small for gestational age.mp.; OR neonatal.mp.; OR neonate.mp.; OR newborn.mp.; OR young infant.mp.
7	Childbirth	obstetric care.mp. ; OR obstetric services.mp. ; OR (maternal and newborn).mp.; OR (maternal and child).mp. ; OR RMNCH.mp. ; OR mnch.mp. ; OR mnh.mp. ; OR intrapartum.mp. ; OR peripartum.mp. ; OR labour.mp. ; OR labor.mp. ; OR facility delivery.mp. ; OR facility birth.mp. ; OR facility based birth.mp. ; OR institutional birth.mp. ; OR childbirth.mp. ; OR birth.mp. ; OR immediate newborn.mp.
8	Postnatal care	postpartum care.mp. ; OR Postpartum Period/; OR PPC.mp.
9	Child health	Breastfeeding.mp.; OR childhood illness.mp.; OR childhood immunizations.mp.; OR childhood vaccinations.mp.; OR diarrhea.mp.; OR HIV.mp.; OR Immunization/ immunisation.mp.; OR infant feeding.mp.; OR malaria prevention.mp.; OR malaria treatment.mp.; OR maternal and child health interventions.mp.; OR MCH Interventions.mp.; OR newborn

		care.mp.; OR newborn illness.mp.; OR nutrition.mp.; OR pneumonia.mp.; OR treatment of sick children.mp.;
**10**	**Target population/ intervention**	**Term 5 OR Term 6 OR Term 7 OR Term 8 OR Term 9**
11	Setting; LMIC	Developing Countries/; OR ((developing or less* developed or under developed or underdeveloped or middle income or low* income) adj (economy or economies)) .ti,ab. ; OR ((developing or less* developed or under developed or underdeveloped or middle income or low* income or underserved or under served or deprived or poor*) adj (countr* or nation? or population? or world.ti,ab.; OR (low* adj (gdp or gnp or gross domestic or gross national)) .ti,ab. ; OR (low adj3 middle adj3 countr*).ti,ab.; OR (lmic or lmics or third world or lami countr.ti,ab.; OR transitional countr.ti,ab.; OR global south.ti,ab.; OR Democratic People's Republic of Korea/; OR (North Korea or (Democratic People* Republic adj2 Korea)) .ti,ab.; OR Cambodia/; OR Cambodia.ti,ab. ; OR Indonesia/; OR (Indonesia or Dutch East Indies) .ti,ab. ; OR (Kiribati or Gilbert Islands or Phoenix Islands or Line Islands) .ti,ab.; OR Laos/; OR (Laos or (Lao adj1 Democratic Republic)) .ti,ab. ; OR Micronesia/; OR Micronesia.ti,ab.; OR Mongolia/; OR Mongolia.ti,ab. ; OR Myanmar/; OR (Myanmar or Burma).ti,ab.; OR Papua New Guinea/; OR (Papua New Guinea or German New Guinea or British New Guinea or Territory of Papua) .ti,ab. ; OR Philippines/; OR (Philippines or Philippine Islands) .ti,ab. ; OR Solomon Islands.ti,ab.; OR Timor-Leste/; OR (Timor-Leste or East Timor or Portuguese Timor) .ti,ab. ; OR Vanuatu/; OR (Vanuatu or New Hebrides) .ti,ab. ; OR Vietnam/; OR (Viet Nam or Vietnam or French Indochina) .ti,ab.; OR American Samoa/; OR American Samoa.ti,ab.; OR exp China/; OR China.ti,ab.; OR Fiji/; OR Fiji.ti,ab. ; OR Malaysia/; OR (Malaysia or Malayan Union or Malaya) .ti,ab. ; OR Marshall Islands.ti,ab.; OR Nauru.ti,ab.; OR Independent State of Samoa"/; OR ((Samoa not American Samoa) or Western Samoa or Navigator Islands or Samoan Islands) .ti,ab. ; OR Thailand/; OR (Thailand or Siam) .ti,ab. ; OR Tonga/; OR Tonga.ti,ab.; OR (Tuvalu or Ellice Islands) .ti,ab. ; OR Melanesia/; OR Melanesia.ti,ab.; OR Polynesia/; OR Polynesia.ti,ab.; OR Kyrgyzstan/; OR (Kyrgyzstan or Kyrgyz Republic or Kirghizia or Kirghiz) .ti,ab. ; OR Moldova/; OR Moldova.ti,ab.; OR Ukraine/; OR Ukraine.ti,ab; OR Uzbekistan/; OR Uzbekistan.ti,ab.; OR Albania/; OR Albania.ti,ab.; OR Armenia/ ; OR Armenia.ti,ab.; OR Azerbaijan/; OR Azerbaijan.ti,ab.; OR "Republic of Belarus"/; OR (Belarus or Byelarus or Byelorussia or Belorussia) .ti,ab.; OR Bosnia-Herzegovina/; OR (Bosnia or Herzegovina) .ti,ab. ; OR Bulgaria/ ; OR Bulgaria.ti,ab.; OR Georgia (Republic)" .ti,ab. ; OR Georgia.ti,ab. not Georgia/ ; OR Kazakhstan/; OR (Kazakhstan or Kazakh) .ti,ab. ; OR Kosovo/; OR Kosovo.ti,ab.; OR Montenegro/; OR Montenegro.ti,ab.; OR Republic of North Macedonia"/; OR North Macedonia.ti,ab.; OR Romania/; OR Romania.ti,ab.; OR exp Russia/; OR Russia (Pre-1917)"/ ; OR USSR/; OR (Russia or Russian Federation or USSR or Union of Soviet Socialist Republics or Soviet Union) .ti,ab. ; OR Serbia/; OR Serbia/; OR Turkey/; OR (Turkey.ti,ab. not animal/) or (Anatolia or Asia Minor) .ti,ab. ; OR Turkmenistan/; OR Turkmenistan.ti,ab.; OR Tajikistan/; OR Tajikistan.ti,ab.; OR Asia, Central/; OR Asia, Northern/; OR Central Asia.ti,ab.; OR Haiti/; OR (Haiti or Hayti) .ti,ab. ; OR Bolivia/; OR Bolivia.ti,ab.; OR El Salvador/; OR El Salvador.ti,ab.; OR Honduras/; OR Honduras.ti,ab.; OR Nicaragua/; OR Nicaragua.ti,ab.; OR Argentina/; OR (Argentina or Argentine Republic) .ti,ab. ; OR Belize/; OR (Belize or British Honduras) ; OR Brazil/; OR Brazil; OR Colombia/; OR Colombia.ti,ab.; OR Costa Rica/; OR Costa Rica.ti,ab.; OR Cuba/; OR Cuba.ti,ab.; OR Dominica/; OR Dominica.ti,ab.; OR Dominican Republic/ ; OR Dominican Republic.ti,ab.; OR Ecuador/; OR

		Ecuador.ti,ab.; OR Grenada/; OR Grenada.ti,ab.; OR Guatemala/; OR Guatemala.ti,ab.; OR Guyana/; OR (Guyana or British Guiana) .ti,ab. ; OR Jamaica/; OR Jamaica.ti,ab.; OR Mexico/; OR (Mexico or United Mexican States) .ti,ab. ; OR Paraguay/; OR Paraguay.ti,ab.; OR Peru/; OR Peru.ti,ab.; OR Saint Lucia/; OR (St Lucia or Saint Lucia or Iyonala or Hewanorra) .ti,ab. ; OR "Saint Vincent and the Grenadines"/; OR (Saint Vincent or St Vincent or Grenadines) .ti,ab. ; OR Suriname/; OR (Suriname or Dutch Guiana) .ti,ab. ; OR Venezuela/; OR Venezuela.ti,ab.; OR Djibouti/; OR (Djibouti or French Somaliland) .ti,ab. ; OR Egypt/; OR Egypt.ti,ab.; OR Morocco/; OR Morocco.ti,ab.; OR Tunisia/; OR Tunisia.ti,ab.; OR (Gaza or West Bank or Palestine) .ti,ab. ; OR Algeria/; OR Algeria.ti,ab.; OR Iran/; OR (Iran or Persia) .ti,ab. ; OR Iraq/; OR (Iraq or Mesopotamia) .ti,ab. ; OR Jordan/; OR Jordan.ti,ab.; OR Lebanon/; OR (Lebanon or Lebanese Republic) .ti,ab. ; OR Libya/; OR Libya.ti,ab.; OR Syria/; OR (Syria or Syrian Arab Republic) .ti,ab. ; OR Yemen/; OR Yemen.ti,ab.; OR Afghanistan/; OR Afghanistan.ti,ab.; OR Nepal/; OR Nepal.ti,ab.; OR Bangladesh/; OR Bangladesh.ti,ab.; OR Bhutan/; OR Bhutan.ti,ab.; OR exp India/; OR India.ti,ab.; OR Pakistan/; OR Pakistan.ti,ab.; OR Maldives.ti,ab.; OR Sri Lanka/; OR (Sri Lanka or Ceylon) ; OR Angola/; OR Angola.ti,ab.; OR Cameroon/; OR (Cameroon or Kamerun or Cameroun) .ti,ab. ; OR Cape Verde/; OR (Cape Verde or Cabo Verde) .ti,ab. ; OR Comoros/; OR (Comoros or Glorioso Islands or Mayotte) .ti,ab. ; OR Congo/; OR (Congo not ((Democratic Republic adj3 Congo) or congo red or crimean-congo)) .ti,ab. ; OR Cote d'Ivoire/; OR (Cote d'Ivoire or Cote dIvoire or Ivory Coast) .ti,ab. ; OR Eswatini/; OR (eSwatini or Swaziland) .ti,ab. ; OR Ghana/; OR (Ghana or Gold Coast) ; OR Kenya/; OR (Kenya or East Africa Protectorate) .ti,ab. ; OR Lesotho/; OR (Lesotho or Basutoland) .ti,ab. ; OR Mauritania/; OR Mauritania.ti,ab.; OR Nigeria/; OR Nigeria.ti,ab.; OR (Sao Tome adj2 Principe) ; OR Senegal/; OR Senegal.ti,ab.; OR Sudan/; OR (Sudan not South Sudan) .ti,ab. ; OR Zambia/; OR (Zambia or Northern Rhodesia) .ti,ab.; OR Zimbabwe/; OR (Zimbabwe or Southern Rhodesia) .ti,ab. ; OR Botswana/; OR (Botswana or Bechuanaland or Kalahari) .ti,ab. ; OR Equatorial Guinea/; OR (Equatorial Guinea or Spanish Guinea) .ti,ab. ; OR Gabon/; OR (Gabon or Gabonese Republic) .ti,ab. ; OR Mauritius/; OR (Mauritius or Agalega Islands) .ti,ab. ; OR Namibia/; OR (Namibia or German South West Africa) .ti,ab. ; OR South Africa/; OR (South Africa or Cape Colony or British Bechuanaland or Boer Republics or Zululand or Transvaal or Natalia Republic or Orange Free State) .ti,ab. ; OR Benin/; OR (Benin or Dahomey) Burkina Faso/ (Burkina Faso or Burkina Fasso or Upper Volta) .ti,ab. ; OR Burundi/; OR (Burundi or Ruanda-Urundi) .ti,ab. ; OR Central African Republic/; OR (Central African Republic or Ubangi-Shari) .ti,ab. ; OR Chad/; OR Chad.ti,ab.; OR Democratic Republic of the Congo.ti,ab.; OR (((Democratic Republic or DR) adj2 Congo) or Congo-Kinshasa or Belgian Congo or Zaire or Congo Free State) .ti,ab. ; OR Eritrea/; OR Eritrea.ti,ab.; OR Ethiopia/; OR (Ethiopia or Abyssinia) .ti,ab. ; OR Gambia/; OR Gambia; OR Guinea/; OR (Guinea not (New Guinea or Guinea Pig* or Guinea Fowl or Guinea-Bissau or Portuguese Guinea or Equatorial Guinea)) .ti,ab. ; OR Guinea-Bissau/; OR (Guinea-Bissau or Portuguese Guinea) .ti,ab. ; OR Liberia/; OR Liberia.ti,ab.; OR Madagascar/; OR (Madagascar or Malagasy Republic) .ti,ab.; OR Malawi/; OR (Malawi or Nyasaland) .ti,ab. ; OR Mali/; OR Mali.ti,ab.; OR Mozambique/; OR (Mozambique or Mocambique or Portuguese East Africa) .ti,ab. ; OR Niger/; OR (Niger not (Aspergillus or Peptococcus or Schizothorax or Cruciferae or Gobius or Lasius or Agelastes or Melanosuchus or radish or Parastromateus or Orius or Apergillus or Parastromateus or Stomoxys)) .ti,ab. ; OR Rwanda/; OR (Rwanda or Ruanda) .ti,ab. ; OR Sierra Leone/; OR (Sierra Leone or Salone) .ti,ab.;

		OR Somalia/; OR (Somalia or Somaliland) .ti,ab.; OR South Sudan/; OR South Sudan.ti,ab.; OR Tanzania/; OR (Tanzania or Tanganyika or Zanzibar) .ti,ab. ; OR Togo/; OR (Togo or Togolese Republic or Togoland) .ti,ab.; OR Uganda/; OR Uganda.ti,ab.; OR "africa south of the sahara"/; OR africa, central/; OR africa, eastern/; OR africa, southern/; OR africa, western/; OR ("Africa South of the Sahara" or sub-Saharan Africa or subSaharan Africa) .ti,ab. ; OR Central Africa.ti,ab.; OR Eastern Africa.ti,ab.; OR Southern Africa.ti,ab.; OR Western Africa.ti,ab.
**11**	**Final search**	**Term 4 AND Term 10 AND Term 11**

APPENDIX S2 – STUDIES EXCLUDED AT FULL-TEXT REVIEW STAGE

Table S2 Studies excluded at full-text review stage and reasons for exclusion

Reference	Reason for exclusion
Aaron G. et al. (2016) "Household coverage of fortified staple food commodities in Rajasthan, India." Plos One 2016;11:e0163176.	Population in need not relevant
Aaron, G et al. (2017). "Coverage of large-scale food fortification of edible oil, wheat flour, and maize flour varies greatly by vehicle and country but is consistently lower among the most vulnerable: results from coverage surveys in 8 countries J Nutr 2017;147:984S–94.	Population in need not relevant
Abd El Razik, M. S. and Salem, M.R. (2019). "From public health and demographic research to decision making: An intervention study in Giza Governorate-Egypt." Evaluation and Program Planning 77: 101704.	Outcome not a population level measure: denominator not population in need
Afolabi, R.F., et al. (2021) "Ethnicity as a cultural factor influencing complete vaccination among children aged 12-23 months in Nigeria." Human Vaccines & Immunotherapeutics 17(7): 2008.	No adjustment for quality of care
Aina, M., et al. (2017). "Preliminary results from direct-to-facility vaccine deliveries in Kano, Nigeria." Vaccine 35(17): 2175-2182.	Outcome not a population level measure: denominator not population in need
Akech, S., et al. (2019). "Magnitude and pattern of improvement in processes of care for hospitalised children with diarrhoea and dehydration in Kenyan hospitals participating in a clinical network." Tropical Medicine & International Health 24(1): 73-80.	Outcome not a population level measure: denominator not population in need
Alfiah, E., et al. (2019). "Coverage and adherence of weekly iron folic acid supplementation among school going adolescent girls in Indonesia." Annals of Nutrition and Metabolism 75 (3): 324.	Population in need not relevant
Allan, S., et al. (2021) "Inequities in childhood immunisation coverage associated with socioeconomic, geographic, maternal, child, and place of birth characteristics in Kenya." BMC Infectious Diseases 21:553.	No adjustment for quality of care
Allen, S. M., et al. (2017). "Measuring facility capability to provide routine and emergency childbirth care to mothers and newborns: An appeal to adjust for delivery caseload of facilities." PLoS ONE [Electronic Resource] 12(10): e0186515.	Outcome not a population level measure: denominator not population in need
Ampadu, H. H., et al. (2019). "Prescribing patterns and compliance with World Health Organization recommendations for the management of severe malaria: a modified cohort event monitoring study in public health facilities in Ghana and Uganda." Malaria Journal 18(1): 36.	Outcome not a population level measure: denominator not population in need
Ansari, N., et al. (2020). "Quality of care in prevention, detection and management of postpartum hemorrhage in hospitals in Afghanistan: an observational assessment." BMC Health Services Research 20(1): 484.	Relevance
Arsenault, C., et al. (2021) "Patient volume and quality of primary care in Ethiopia: findings from the routine health information system and the 2014 Service Provision Assessment survey." BMC Health Services Research 21:485.	Outcome not a population level measure: denominator not population in need
Ayieko, P., et al. (2019). "Effect of enhancing audit and feedback on uptake of childhood pneumonia treatment policy in hospitals that are part of a clinical network: a cluster randomized trial." Implementation Science 14(1): 20.	Outcome not a population level measure: denominator not population in need
Benzaken, C. L., et al. (2020). "Development of a cumulative metric of vaccination adherence behavior and its application among a cohort of 12-month-olds in western Kenya." Vaccine 38(18): 3429-3435.	No adjustment for quality of care
Bhattacharya, A. A., et al. (2019). "Monitoring childbirth care in primary health facilities: a validity study in Gombe State, northeastern Nigeria." Journal of Global Health 9(2): 020411.	Study type
Bhura, M., et al. (2020). "Evaluating implementation of "management of Possible Serious Bacterial Infection (PSBI) when referral is not feasible" in primary health care facilities in Sindh province, Pakistan." PLoS ONE [Electronic Resource] 15(10): e0240688.	Outcome not a population level measure: denominator not population in need

Reference	Reason for exclusion
Biset, G., et al. (2021) "Full immunization coverage and associated factors among children age 12-23 months in Ethiopia: systematic review and meta-analysis of observational studies." Human Vaccines & Immunotherapeutics 17 (7): 2326.	Study type
Brenner, S., et al. (2017). "Implementation research to improve quality of maternal and newborn health care, Malawi." Bulletin of the World Health Organization 95(7): 491-502.	Outcome not a population level measure: denominator not population in need
Buchmann, E. J. (2020). "Quality and readiness for facility-based childbirth in sub-Saharan Africa." BJOG: An International Journal of Obstetrics & Gynaecology 127(12): 1547.	Study type
Budu, E. et al. (2021). "Maternal healthcare utilization and full immunization coverage among 12–23 months children in Benin: a cross sectional study using population-based data." Archives of Public Health 79:34.	No adjustment for quality of care
Burke, D. (2018). "Advanced distribution of misoprostol for prevention of postpartum hemorrhage at home births in Haiti." International Journal of Gynecology and Obstetrics 143 (Supplement 3): 461.	Relevance
Carter, E. D., et al. (2018). "An agent-based model of effective coverage of appropriate management of child illness." American Journal of Tropical Medicine and Hygiene 99 (4 Supplement): 254.	Outcome not a population level measure: denominator not population in need
Carvajal-Aguirre, L., et al. (2017). "Does health facility service environment matter for the receipt of essential newborn care? Linking health facility and household survey data in Malawi." Journal of Global Health 7(2): 020508.	Outcome not a population level measure: denominator not population in need
Choi, S., et al. (2020). "Improved care and survival in severe malnutrition through eLearning." Archives of Disease in Childhood 105(1): 32-39.	Relevance
Cohen, J. L., et al. (2020). "Quality of clinical management of children diagnosed with malaria: A cross-sectional assessment in 9 sub-Saharan African countries between 2007-2018." PLoS Medicine / Public Library of Science 17(9): e1003254.	Outcome not a population level measure: denominator not population in need
Colson, K., et al. (2013). "Comparative estimates of immunisation coverage from three different sources: results from the SM2015 evaluation. Lancet. 2013;381:S32.	Results presented in another article
Colson, K., et al. (2015). "Comparative Estimates of Crude and Effective Coverage of Measles Immunization in Low-Resource Settings-Findings from Salud Mesoamérica 2015." PLoS ONE 10(7): e0130697	Outcome not a population level measure: denominator not population in need
Daka, D. W., et al. (2020). "Quality of clinical assessment and management of sick children by Health Extension Workers in four regions of Ethiopia: A cross-sectional survey." PLoS ONE [Electronic Resource] 15(9): e0239361.	Outcome not a population level measure: denominator not population in need
Das, M. K., et al. (2019). "Impact of Neonatal Resuscitation Capacity Building of Birth Attendants on Stillbirth Rate at Public Health Facilities in Uttar Pradesh, India." Indian pediatrics 56(5): 369-373.	Outcome not a population level measure: denominator not population in need
Day, L. et al. (2021). "Assessment of the validity of the measurement of newborn and maternal health-care coverage in hospitals (EN-BIRTH): an observational study." Lancet Global Health 9: e267.	Outcome not a population level measure: denominator not population in need
Deming, M.S., et al. (2002) "Tetanus toxoid coverage as an indicator of serological protection against neonatal tetanus"". Bull World Health Organ 2002;80:696–703.	Population in need not relevant
Eboreime, E. A., et al. (2019). "Effectiveness of the Diagnose-Intervene- Verify-Adjust (DIVA) model for integrated primary healthcare planning and performance improvement: an embedded mixed methods evaluation in Kaduna state, Nigeria." BMJ Open 9(3): e026016.	Outcome not a population level measure: denominator not population in need
Eboreime, E. A., et al. (2019). "Primary healthcare planning, bottleneck analysis and performance improvement: An evaluation	Outcome not a population level measure: denominator not population in need

Reference	Reason for exclusion
of processes and outcomes in a Nigerian context." Evaluation & Program Planning 77: 101712.	
Engle-Stone, R. et al. (2015). "Estimating the effective coverage of programs to control vitamin a deficiency and its consequences among women and young children in Cameroon. Food Nutr Bull. 2015;36(3 Suppl):S149–71.	Study type
Ezran, C., et al. (2019). "Assessing trends in the content of maternal and child care following a health system strengthening initiative in rural Madagascar: A longitudinal cohort study." PLoS Medicine 16 (8): (no pagination)(e1002869).	Relevance
Fink, G., et al. (2020). "Antibiotic exposure among children younger than 5 years in low-income and middle-income countries: a cross-sectional study of nationally representative facility-based and household-based surveys." The Lancet Infectious Diseases 20(2): 179-187.	Intervention type
Fisseha, G., et al. (2019). "Quality of intrapartum and newborn care in Tigray, Northern Ethiopia." BMC Pregnancy & Childbirth 19(1): 37.	Outcome not a population level measure: denominator not population in need
Francetic, I., et al. (2019). "Going operational with health systems governance: supervision and incentives to health workers for increased quality of care in Tanzania." Health Policy & Planning 34(Supplement_2): ii77-ii92.	Outcome not a population level measure: denominator not population in need
Fullman, N., et al. (2017). "Measuring progress and projecting attainment on the basis of past trends of the health-related Sustainable Development Goals in 188 countries: an analysis from the Global Burden of Disease Study 2016." The Lancet 390(10100): 1423-1459.	No adjustment for quality of care
Gage, A. D., et al. (2018). "Does quality influence utilization of primary health care? Evidence from Haiti." Global Health 14(1): 59.	Relevance
Gakidou, E., et al. (2006). "Assessing the effect of the 2001–06 Mexican health reform: an interim report card. Lancet (London, England). 2006; 368(9550):1920–35.	No adjustment for quality of care
Galstyan, S. H., et al. (2019). "Cross-sectional study of the quality of neonatal care services in Armenia." International journal of health care quality assurance 32(8): 1145-1161.	Outcome not a population level measure: denominator not population in need
Getachew, T., et al. (2020). "Assessing the quality of care in sick child services at health facilities in Ethiopia." BMC Health Services Research 20(1): 574.	Outcome not a population level measure: denominator not population in need
Goleman, M. J., et al. (2018). "Quality Improvement Initiative to Improve Human Papillomavirus Vaccine Initiation at 9 Years of Age." Academic Pediatrics 18(7): 769-775.	Setting: Columbus, Ohio (USA)
Guzha, B. T., et al. (2018). "Assessment of quality of obstetric care in Zimbabwe using the standard primipara." BMC Pregnancy & Childbirth 18(1): 205.	Outcome not a population level measure: denominator not population in need
Habte, A. et al. (2021). "Determinants of practice of preconception care among women of reproductive age group in southern Ethiopia, 2020: content analysis." Reproductive Health 18:100.	Intervention type
Hayford (2013) Measles vaccination coverage estimates from surveys, clinic records, and immune markers in oral fluid and blood: a population-based cross-sectional study	Outcome not a population level measure: denominator not population in need
Horwood, C., et al. (2017). "A continuous quality improvement intervention to improve the effectiveness of community health workers providing care to mothers and children: a cluster randomised controlled trial in South Africa." Human Resources for Health [Electronic Resource] 15(1): 39.	No adjustment for quality of care
Huybregts, L., et al. (2019). "Impact on child acute malnutrition of integrating small-quantity lipid-based nutrient supplements into community-level screening for acute malnutrition: A cluster-randomized controlled trial in Mali." PLoS Medicine / Public Library of Science 16(8): e1002892.	No adjustment for quality of care
Idzerda, L. (2011). Access to primary healthcare services for the Roma population in Serbia: a secondary data analysis. BMC Int Health Human Rights. 2011;11:10.	No adjustment for quality of care
Kamath, A. M., et al. (2020). "Assessing multidimensional care coverage for pre-eclampsia in the era of universal health coverage:	Intervention type

Reference	Reason for exclusion
A pre-post evaluation of the Salud Mesoamerica Initiative." International Journal of Gynaecology & Obstetrics 149(3): 318-325.	
Kanyangarara, M. and V. B. Chou (2017). "Linking household surveys and health facility assessments to estimate intervention coverage for the Lives Saved Tool (LiST)." BMC Public Health 17(Suppl 4): 780.	Intervention type
Karim, A., et al. (2020). "A systems approach to assessing complexity in health interventions: an effectiveness decay model for integrated community case management." Global health action 13(1).	Study type
Kc, A., et al. (2020). "Quality of Care for Maternal and Newborn Health in Health Facilities in Nepal." Maternal & Child Health Journal 24(Suppl 1): 31-38.	Outcome not a population level measure: denominator not population in need
Khan, Z., et al. (2000) Coverage and Efficacy of Measles Immunization in Rural Areas of Aligarh	Outcome not a population level measure: denominator not population in need
Khumalo, P. N., et al. (2020). "The Cascade of Care From Routine Point-of-Care HIV Testing at Birth: Results From an 18-Months Pilot Program in Eswatini." Journal of Acquired Immune Deficiency Syndromes (1999) 84(Supplement 1): S22-S27.	Outcome not a population level measure: denominator not population in need
Kim, S. S., et al. (2019). "Behavior Change Interventions Delivered through Interpersonal Communication, Agricultural Activities, Community Mobilization, and Mass Media Increase Complementary Feeding Practices and Reduce Child Stunting in Ethiopia." Journal of Nutrition 149(8): 1470-1481.	No adjustment for quality of care
Klootwijk, L., et al. (2019). "Challenges affecting prompt access to adequate uncomplicated malaria case management in children in rural primary health facilities in Chikhwawa Malawi." BMC Health Services Research 19(1): 735.	No adjustment for quality of care
Koulidiati, J. L., et al. (2018). "Factors associated with effective coverage of child health services in Burkina Faso." Tropical Medicine & International Health 23(11): 1188-1199.	Duplicate
Koulidiati, J. L., et al. (2018). "Measuring effective coverage of curative child health services in rural Burkina Faso: a cross-sectional study." BMJ Open 8(5): e020423.	Duplicate
Koulidiati, J-L. et al. (2021). "Impact of Performance-Based Financing on effective coverage for curative child health services in Burkina Faso: Evidence from a quasi-experimental design" Tropical Medicine and International Health 8:1002.	Results presented in another article
Kruk, M. E., et al. (2017). "Variation in quality of primary-care services in Kenya, Malawi, Namibia, Rwanda, Senegal, Uganda and the United Republic of Tanzania." Bulletin of the World Health Organization 95(6): 408-418.	Outcome not a population level measure: denominator not population in need
Kruk, M. E., et al. (2018). "Content of Care in 15,000 Sick Child Consultations in Nine Lower-Income Countries." Health Services Research 53(4): 2084-2098.	Outcome not a population level measure: denominator not population in need
Lama, T. P., et al. (2020). "Assessment of facility and health worker readiness to provide quality antenatal, intrapartum and postpartum care in rural Southern Nepal." BMC Health Services Research 20(1): 16.	Outcome not a population level measure: denominator not population in need
Langston, A., et al. (2019). "Testing a simplified tool and training package to improve integrated Community Case Management in Tanganyika Province, Democratic Republic of Congo: a quasi-experimental study." Journal of Global Health 9(1): 010810.	Outcome not a population level measure: denominator not population in need
Lansky, S., et al. (2018). "Monitoring care during childbirth to reduce maternal and infant mortality in Belo Horizonte, Brazil." International Journal of Gynecology and Obstetrics 143 (Supplement 3): 494-495.	Not retrieved
Larson, E., et al. (2019). "Effect of a maternal and newborn health system quality improvement project on the use of facilities for childbirth: a cluster-randomised study in rural Tanzania." Tropical Medicine & International Health 24(5): 636-646.	No adjustment for quality of care
Lauria, M. E., et al. (2019). "Assessing the Integrated Community-Based Health Systems Strengthening initiative in northern Togo: a	Study type

Reference	Reason for exclusion
pragmatic effectiveness-implementation study protocol." Implementation Science 14(1): 92.	
Lazzerini, M., et al. (2017). "Improving the quality of hospital care for children by supportive supervision: a cluster randomized trial, Kyrgyzstan." Bulletin of the World Health Organization 95(6): 397-407.	Relevance
Lazzerini, M., et al. (2019). "Nutritional services for children in Beira, Mozambique: a study reporting on participatory use of data to generate quality improvement recommendations." BMJ open quality 8(4): e000758.	Outcome not a population level measure: denominator not population in need
Lee, J., et al. (2017). "Strategy for integrated linkages between service delivery and household utilization across programs." Annals of Nutrition and Metabolism 71 (Supplement 2): 115.	Study type
Leslie, H. H., et al. (2017). "Association between infrastructure and observed quality of care in 4 healthcare services: A cross-sectional study of 4,300 facilities in 8 countries." PLoS Medicine / Public Library of Science 14(12): e1002464.	Outcome not a population level measure: denominator not population in need
Leyvraz, M., et al. (2017). "Coverage of nutrition interventions intended for infants and young children varies greatly across programs: Results from coverage surveys in 5 countries." Journal of Nutrition 147(5): 995S-1003S.	Results presented in another article
Lozano, R., et al. (2006). "Benchmarking of performance of Mexican states with effective coverage. Lancet 2006;368:1729–41.	Population in need not relevant
Lozano, R., et al. (2020). "Measuring universal health coverage based on an index of effective coverage of health services in 204 countries and territories, 1990–2019: a systematic analysis for the Global Burden of Disease Study 2019." The Lancet 396(10258): 1250-1284.	Study type
Luo, H., et al. (2016). Predicted effects of current and potential micronutrient intervention programs on adequacy of folate and vitamin B-12 intake in a national sample of women and young children in Cameroon. FASEB J. 2016;30((Luo H.; Stewart C.P.; Brown K.H.; Engle-Stone R.) Program in International and Community Nutrition, University of California, Davis, United States).	Population in need not relevant
Manyazewal, T., et al. (2018). "Improving immunization capacity in Ethiopia through continuous quality improvement interventions: a prospective quasi-experimental study." Infectious Diseases of Poverty 7(1): 119.	Relevance
Martinez, S. et al., (2011). "Effective coverage of health interventions in Latin America and the Caribbean: metrics for the assessment of health systems performance. Salud Publica Mex. 2011;53(SUPPL. 2):S78–84	Not in English
Maves, K., et al. (2020). "Rapid baseline assessment of peripartum care delivery by skilled birth attendants in rural India." Journal of Investigative Medicine 68 (1): A39.	Outcome not a population level measure: denominator not population in need
McGuire, F., et al. (2021). " The effect of distance on maternal institutional delivery choice: Evidence from Malawi." Health Economics 1:24.	Outcome not a population level measure: denominator not population in need
Minta, A. A., et al. (2020). "Seroprevalence of Measles, Rubella, Tetanus, and Diphtheria Antibodies among Children in Haiti, 2017." American Journal of Tropical Medicine & Hygiene 103(4): 1717-1725.	Outcome not a population level measure: denominator not population in need
Minta, A. et al. (2021). "Hepatitis B surface antigen seroprevalence among children in the Philippines, 2018." Vaccine 39: 1982.	No adjustment for quality of care
Morof, D., et al. (2019). "Addressing the Third Delay in Saving Mothers, Giving Life Districts in Uganda and Zambia: Ensuring Adequate and Appropriate Facility-Based Maternal and Perinatal Health Care." Global Health Science & Practice 7(Suppl 1): S85-S103.	Outcome not a population level measure: denominator not population in need
Mothupi, M. C., et al. (2018). "Measurement approaches in continuum of care for maternal health: a critical interpretive synthesis of evidence from LMICs and its implications for the South African context." BMC Health Services Research 18(1): 539.	Study type

Reference	Reason for exclusion
Mukamurigo, J., et al. (2019). "Quality of intrapartum care for healthy women with spontaneous onset of labour in Rwanda: A health facility-based, cross-sectional study." Sexual & reproductive healthcare : official journal of the Swedish Association of Midwives 19: 78-83.	Outcome not a population level measure: denominator not population in need
Munabi-Babigumira, S., et al. (2019). "Implementing the skilled birth attendance strategy in Uganda: a policy analysis." BMC Health Services Research 19(1): 655.	Study type
Munos, M. K., et al. (2017). "Improving coverage measurement for reproductive, maternal, neonatal and child health: gaps and opportunities." Journal of Global Health 7(1): 010801.	Study type
Mutua, M., et al. (2021). "Inequities in On-Time Childhood Vaccination: Evidence From Sub-Saharan Africa." Americal Journal of Preventive Medicine 60(1S1):S11.	No adjustment for quality of care
Mwapasa, V., et al. (2017). "Impact of Mother-Infant Pair Clinics and Short-Text Messaging Service (SMS) Reminders on Retention of HIV-Infected Women and HIV-Exposed Infants in eMTCT Care in Malawi: A Cluster Randomized Trial." Journal of Acquired Immune Deficiency Syndromes: JAIDS 75 Suppl 2: S123-S131.	Outcome not a population level measure: denominator not population in need
Mwita, S. K., et al. (2019). "Engagement of National Stakeholders and Communities on Health-Care Quality Improvement: Experience from the Implementation of the Partnership for HIV-Free Survival in Tanzania." Journal of the International Association of Providers of AIDS Care 18: 2325958219847454.	Outcome not a population level measure: denominator not population in need
Nagar, R., et al. (2018). "A cluster randomized trial to determine the effectiveness of a novel, digital pendant and voice reminder platform on increasing infant immunization adherence in rural Udaipur, India." Vaccine 36(44): 6567-6577.	Outcome not a population level measure: denominator not population in need
Nanthavong (2015) Diphtheria in Lao PDR: Insufficient Coverage or Ineffective Vaccine?	Outcome not a population level measure: denominator not population in need
Ngoma, T., et al. (2019). "Addressing the Second Delay in Saving Mothers, Giving Life Districts in Uganda and Zambia: Reaching Appropriate Maternal Care in a Timely Manner." Global Health Science & Practice 7(Suppl 1): S68-S84.	Outcome not a population level measure: denominator not population in need
Nguyen, P. H., et al. (2018). "Importance of coverage and quality for impact of nutrition interventions delivered through an existing health programme in Bangladesh." Maternal & Child Nutrition 14(4): e12613.	Relevance
Nguyen, P., et al. (2020). "Quality-Adjusted Coverage of Nutrition Interventions Across the Continuum of Care: Insights from Household and Health Facility Data in Bangladesh." Current Developments in Nutrition 4(Suppl 2): 254-254.	Abstract, insufficient data
Nikiema (2017) Effectiveness of facility-based personalized maternal nutrition counseling in improving child growth and morbidity up to 18 months: A cluster-randomized controlled trial in	Relevance
Odjidja, E. N., et al. (2019). "Delivery of integrated infectious disease control services under the new antenatal care guidelines: a service availability and readiness assessment of health facilities in Tanzania." BMC Health Services Research 19(1): 153.	Outcome not a population level measure: denominator not population in need
Ojha, C. R., et al. (2017). "Impact of mass drug administration for elimination of lymphatic filariasis in Nepal." PLoS Neglected Tropical Diseases [electronic resource] 11(7): e0005788.	Relevance
Okawa, S., et al. (2019). "Effect of continuum-of-care intervention package on improving contacts and quality of maternal and newborn healthcare in Ghana: A cluster randomised controlled trial." BMJ Open 9 (9) (no pagination)(e025347).	Outcome not a population level measure: denominator not population in need
Oresanya, O., et al. (2019). "Effect of community-based intervention on improving access to treatment for sick under-five children in hard-to-reach communities in Niger State, Nigeria." Journal of Global Health 9(1): 010803.	No adjustment for quality of care
Owili, P. O., et al. (2017). "Quality of maternity care and its determinants along the continuum in Kenya: A structural equation modeling analysis." PLoS ONE [Electronic Resource] 12(5): e0177756	Outcome not a population level measure: denominator not population in need

Reference	Reason for exclusion
Page-Shipp, L., et al. (2018). "Household point of care CD4 testing and isoniazid preventive therapy initiation in a household TB contact tracing programme in two districts of South Africa." PLoS ONE [Electronic Resource] 13(3): e0192089.	Intervention type
Pallangyo, E., et al. (2017). "Improved postpartum care after a participatory facilitation intervention in Dar es Salaam, Tanzania: a mixed method evaluation." Glob Health Action 10(1): 1295697.	Outcome not a population level measure: denominator not population in need
Pugliese-Garcia, M., et al. (2020). "Childbirth care in Egypt: a repeat cross-sectional analysis using Demographic and Health Surveys between 1995 and 2014 examining use of care, provider mix and immediate postpartum care content." BMC Pregnancy & Childbirth 20(1): 46.	Outcome not a population level measure: denominator not population in need
Qazi, U., et al. (2019). "Compliance to timely vaccination in an Expanded Program on Immunization center of Pakistan." Vaccine 37(32): 4618-4622.	Outcome not a population level measure: denominator not population in need
Rajbhandari, S. P., et al. (2017). "Postpartum hemorrhage prevention in Nepal: a program assessment." BMC Pregnancy & Childbirth 17(1): 169.	Relevance
Ram, P. K., et al. (2017). "Coverage gaps in early initiation of breastfeeding among newborns, sub-saharan Africa, 2010-2015." American Journal of Tropical Medicine and Hygiene 97 (5 Supplement 1): 285.	Abstract, insufficient data
Randive, B.B., (2013). Effective coverage of institutional deliveries under the Janani Suraksha Yojana programme in high maternal mortality provinces of India: analysis of data from an annual health survey. Lancet. 2013;381:S32.	Abstract, insufficient data
Razavi-Shearer, D., et al. (2018). "Global prevalence, treatment, and prevention of hepatitis B virus infection in 2016: a modelling study." The Lancet Gastroenterology & Hepatology 3(6): 383-403	Relevance
Rivera, D., et al. (2017). "Integrated community case management (iCCM) of childhood infection saves lives in hard-to-reach communities in Nicaragua." Pan American Journal of Public Health 41: e66	Outcome not a population level measure: denominator not population in need
Rogers, E., et al. (2018). "Quality of care of treatment for uncomplicated severe acute malnutrition provided by lady health workers in Pakistan." Public Health Nutrition 21(2): 385-390.	Outcome not a population level measure: denominator not population in need
Saaka, M., et al. (2018). "Prevalence and determinants of essential newborn care practices in the Lawra District of Ghana." BMC Pediatrics 18(1): 173.	No adjustment for quality of care
Sally, E. T. and E. Kenu (2017). "Evaluation of access and utilization of EPI services amongst children 12-23 months in Kwahu Afram Plains, Eastern region, Ghana." The Pan African medical journal 28: 238.	No adjustment for quality of care
Sami, S., et al. (2018). "Understanding health systems to improve community and facility level newborn care among displaced populations in South Sudan: a mixed methods case study." BMC Pregnancy & Childbirth 18(1): 325	Outcome not a population level measure: denominator not population in need
Sanjel, K., et al. (2019). "Patterns and determinants of essential neonatal care utilization among underprivileged ethnic groups in Midwest Nepal: a mixed method study." BMC Pregnancy & Childbirth 19(1): 310.	Relevance
Semrau, K. E. A., et al. (2017). "Outcomes of a Coaching-Based WHO Safe Childbirth Checklist Program in India." New England Journal of Medicine 377(24): 2313-2324.	Outcome not a population level measure: denominator not population in need
Serván-Mori, E., et al. (2019). "Improving the effective maternal-child health care coverage through synergies between supply and demand-side interventions: Evidence from Mexico." Journal of Global Health 9(2).	Intervention type
Sharma, J., et al. (2018). "Can India's primary care facilities deliver? A cross-sectional assessment of the Indian public health system's capacity for basic delivery and newborn services." BMJ Open 8(6): e020532.	Outcome not a population level measure: denominator not population in need
Sheffel, A., et al. (2019). "Methods for analysis of complex survey data: an application using the Tanzanian 2015 Demographic and	Intervention type

Reference	Reason for exclusion
Health Survey and Service Provision Assessment." Journal of Global Health 9(2): 020902.	
Sindelar, K., et al. (2020). "Beyond the facility: An evaluation of seven community-based pediatric HIV testing strategies and linkage to care outcomes in a high prevalence, resource-limited setting." PLoS ONE [Electronic Resource] 15(9): e0236985.	Outcome not a population level measure: denominator not population in need
Sitrin, D., et al. (2017). "Evidence from household surveys for measuring coverage of newborn care practices." Journal of Global Health 7(2): 020503.	Outcome not a population level measure: denominator not population in need
Soremekun, S., et al. (2018). "Variation in the quality and out-of-pocket cost of treatment for childhood malaria, diarrhoea, and pneumonia: Community and facility based care in rural Uganda." PLoS ONE [Electronic Resource] 13(11): e0200543.	Relevance
Tang, X., et al. (2017). "Timeliness and completeness of measles vaccination among children in rural areas of Guangxi, China: A stratified three-stage cluster survey." Journal of Epidemiology 27: 317e324.	No adjustment for quality of care
Tariku, A., et al. (2020). "Prevention and treatment of suspected pneumonia in Ethiopian children less than five years from household to primary care." Acta Paediatrica, International Journal of Paediatrics.	Relevance
Taylor, C., et al. (2019). "Examination of malaria service utilization and service provision: an analysis of DHS and SPA data from Malawi, Senegal, and Tanzania." Malaria Journal 18(1): 258.	Relevance
Teasdale, C. A., et al. (2017). "High risk of loss to follow-up among South African children on ART during transfer, a retrospective cohort analysis with community tracing." Journal of the International AIDS Society 20(1): 21748.	Outcome not a population level measure: denominator not population in need
Thapa Pachya, A., et al. (2020). "Newborn Service Readiness of Primary Level Health Facilities of Eastern Mountain Region of Nepal." Journal of Nepal Health Research Council 17(4): 431-436.	Outcome not a population level measure: denominator not population in need
Thiam, S., et al. (2019). "Knowledge and practices of mothers and caregivers on diarrhoeal management among under 5-year-old children in a medium-size town of Senegal." Acta Tropica 194: 155-164.	No adjustment for quality of care
Tippins, A., et al. (2017). "Timeliness of childhood vaccination in the Federated States of Micronesia." Vaccine 35: 6404.	No adjustment for quality of care
Tomlin, K., et al. (2020). "Assessing capacity of health facilities to provide routine maternal and newborn care in low-income settings: what proportions are ready to provide good-quality care, and what proportions of women receive it?" BMC Pregnancy & Childbirth 20(1): 289.	Outcome not a population level measure: denominator not population in need
Travassos (2016) Immunization Coverage Surveys and Linked Biomarker Serosurveys in Three Regions in Ethiopia	Outcome not a population level measure: denominator not population in need
Tripura, R., et al. (2018). "A Controlled Trial of Mass Drug Administration to Interrupt Transmission of Multidrug-Resistant Falciparum Malaria in Cambodian Villages." Clinical Infectious Diseases 67(6): 817-826.	Relevance
Tumilowicz, A., et al. (2019). "Bottlenecks and predictors of coverage and adherence outcomes for a micronutrient powder program in Ethiopia." Maternal & Child Nutrition 15(S5): e12807.	No adjustment for quality of care
Tumilowicz, A., et al. (2019). "Mixed methods evaluation explains bypassing of vouchers in micronutrient powder trial in Mozambique." Maternal & Child Nutrition 15(S5): e12718.	No adjustment for quality of care
Ugwa, E., et al. (2018). "Use of maternal and newborn data for decision making by health workers in Ebonyi and Kogi, Nigeria." International Journal of Gynecology and Obstetrics 143 (Supplement 3): 508-509.	Intervention type
van den Ent, M. M. V. X., et al. (2017). "Equity and immunization supply chain in Madagascar." Vaccine 35(17): 2148-2154.	Study type
Wang, W., et al. (2017). "Limited Service Availability, Readiness, and Use of Facility-Based Delivery Care in Haiti: A Study Linking Health Facility Data and Population Data." Global health, science and practice 5(2): 244-260.	Results presented in later article

Reference	Reason for exclusion
Wangdi, K., et ai. (2014). "Prevalence of asymptomatic malaria and bed net ownership and use in Bhutan, 2013: a country earmarked for malaria elimination." Malar J 2014;13.	No adjustment for quality of care
Wanzira, H., et al. (2018). "Quality of care for children with acute malnutrition at health center level in Uganda: a cross sectional study in West Nile region during the refugee crisis." BMC Health Services Research 18(1): 561.	Relevance
Wehrmeister, F. C., et al. (2020). "Wealth-related inequalities in the coverage of reproductive, maternal, newborn and child health interventions in 36 countries in the African Region." Bulletin of the World Health Organization 98(6): 394-405.	No adjustment for quality of care
Wiens, K. E., et al. (2019). "Geographic variation in oral rehydration therapy coverage in low-and middle-income countries, 2000-2017." American Journal of Tropical Medicine and Hygiene 101 (5 Supplement): 399.	No adjustment for quality of care
Wilson, N. (2019). "At-scale evidence from 26 national household surveys on the prevention of mother-to-child transmission of HIV cascade." Health Policy & Planning 34(7): 514-519.	No adjustment for quality of care
Wiradnyani, L. A. A., et al. (2019). "Behind the low and high coverage of vitamin a supplementation program among children 6-59 months in six provinces of Indonesia." Annals of Nutrition and Metabolism 75 (3): 392.	Abstract, insufficient data
Woldeamanuel, B. T. (2020). "Trends and factors associated to early initiation of breastfeeding, exclusive breastfeeding and duration of breastfeeding in Ethiopia: evidence from the Ethiopia Demographic and Health Survey 2016." International Breastfeeding Journal 15(1): 3.	No adjustment for quality of care
Xu, W.-b., et al. (2020). "Investigation and analysis of antibody levels of hepatitis A among children before and after implementing the Expanded National Immunization Program in China." Vaccine 38(4): 878-881.	Outcome not a population level measure: denominator not population in need
Yan, L. D., et al. (2018). "Equity dimensions of the availability and quality of reproductive, maternal and neonatal health services in Zambia." Tropical Medicine & International Health 23(4): 433-445.	Outcome not a population level measure: denominator not population in need
Yawson, A. E., et al. (2017). "Regional disparities in immunization services in Ghana through a bottleneck analysis approach: Implications for sustaining national gains in immunization." Archives of Public Health 75(1).	Relevance
Yawson, A. E., et al. (2017). "The lancet series nutritional interventions in Ghana: a determinants analysis approach to inform nutrition strategic planning." BMC Nutr. 3:27.(doi): 10.1186/s40795-40017-40147-40791. eCollection 42017.	Relevance
Yugbare Belemsaga, D., et al. (2018). "Integration of postpartum care into child health and immunization services in Burkina Faso: findings from a cross-sectional study." Reproductive Health 15(1): 171.	Relevance
Zhang, L., et al. (2018). "Analysis of sero-epidemiological characteristics of varicella in healthy children in Jiangsu Province, China." BMC Infectious Diseases 18(1): 563.	Study type

APPENDIX S3 – OVERVIEW OF INCLUDED STUDIES

Table S3 Overview of included studies

Reference	Intervention/service	Setting	Study aim(s)	Indicator	Numerator	Denominator	Data source(s)		Quality measure(s) (see Appendix D for individual items)	How quality measures derived	Results presented	Limitations of EC measure reported by author
							Household	Facility/provider				
Aaron et al. 2016 (63)	Nutrition: Complementary Feeding Supplement	1 district in the Northern Region & 3 districts in the Eastern Region of Ghana	To assess the effectiveness of the two delivery two sales-based approaches to distributing a complementary food supplement to infants and young children.	Effective Coverage: the proportion of children aged 6 to 24 months whose caregiver fed the child the product at least once in the previous 7 days.	Number of children who consumed the product in the last 7 days.	Children aged between 6 and 24 months.<br><br>(1) all children used as a measure of overall programme performance<br>(2) all children defined as at risk used as a measure of how well delivery model addressed needs.	• Household survey<br>• date n/r	n/a	Receipt and timing of supplement based on caregiver's self-report<br><br>*Binary: taken as prescribed vs. not*	None given, adapted Tanahashi's model.	• Coverage measures, 3 steps: message coverage, contact coverage, & effective coverage	None given
Baker et al. 2015 (55)<br>* EQUIP study	(1) Childbirth: use of partograph to monitor labour<br><br>(2) Childbirth: active management of third stage of labour (AMTSL)<br><br>(3) Postpartum care in a health facility within 48hrs of delivery	2 rural districts in Tanzania	To estimate effective coverage of maternal and newborn health interventions & identify bottlenecks in their implementation in rural districts of the United Republic of Tanzania.	Effective coverage: the proportion of mothers who used a health facility that was ready to deliver the intervention and who actually received the intervention.<br><br>(1) Proportion who satisfy the definition for availability coverage and who used a facility in which a health worker reported using a partograph during the last delivery attended.<br><br>(2) Proportion who satisfy the definition for availability coverage and who used a facility in which a health worker reported giving an oxytocic agent during the last delivery attended	Number of women who gave birth in HF that was able to deliver the intervention and received the intervention	All women aged 13-49 yrs with live birth in previous 12mnths	• Household survey<br>• Nov 2011 – Dec 2012	• Facility survey<br>• incl. interview with HCW on actions taken during last delivery<br>• April-July 2012	Availability of human resources, drugs & equipment needed to deliver intervention & receipt of interventions based on HCW reports.<br><br>(1) partograph available & HCW reported using a partograph during the last delivery attended<br><br>(2) sterile syringes & needles and oxytocin or ergometrine available & HCW reported giving an oxytocic agent during the last delivery attended<br><br>(3) offering postpartum care with iron supplements available & women reported being checked within 48 hours of delivery<br><br>*Binary: all components present vs. not*	None given, adapted Tanahashi's model to develop an implementation pathway.	• Implementation pathway, 4 steps: target population, accessibility coverage (timely utilisation of a health facility), availability coverage (health facility readiness) and effective coverage (receives the interventions as intended).	Indicators chosen affect the coverage estimates. Used indicators that reflected only the minimum conditions required for judging completeness of implementation. HCW reports of actions taken subject to social desirability bias, could result in over reporting.

Reference	Intervention/service	Setting	Study aim(s)	Indicator	Numerator	Denominator	Data source(s)		Quality measure(s) (see Appendix D for individual items)	How quality measures derived	Results presented	Limitations of EC measure reported by author
							Household	Facility/provider				
				(3) Proportion who satisfy the definition for availability coverage and who report being checked within 48 hours of delivery								
Carter et al. 2018 (45)	Sick child care: treatment for diarrhoea, fever, ARI or a combination	1 province in Zambia; two urban and three rural health facility catchment	To assess the feasibility of collecting geographically and temporally concurrent household and health care provider data at a small scale in both an urban and rural setting to perform exact-match linking. To quantify the degree of bias introduced by using less rigorous linking methods, including multiple ecological linking methods and utilization of facility-only health care provider assessments.	Input based effective coverage: average structural quality experienced by all sick children (based on their reported care-seeking behaviour and linked source of care).	Structural quality score of either specific reported source of care or nearest provider.	All children under 5 reported to have at least one DHS illness (diarrhoea, fever, ARI or a combination)	• Household survey based on DHS<br>• March 2016	• Provider assessment based on SARA.<br>• HCW knowledge assessed using clinical case scenarios.<br>• Jan – March 2016.	Structural quality, 6 domains: (i) diagnostics, (ii) basic medicines, (iii) severe/complicated illness medicines, (iv) human resources, (v) availability of services, commodities, and (vi) HCW knowledge.<br><br>*Average score: equal weight given to each domain*	None given, authors state selected the minimum inputs required.	• Composite measure<br>• EC estimates presented by different linking approaches	Measure based on facility capacity to provide care; no measure of process quality or quantitative health gain.<br>Indicators were considered the minimum inputs for appropriate care: the basic commodities required to diagnosis and treat common child illness, along with the human resources and clinical knowledge to apply them correctly
Carvajal-Aguirre et al. 2017 (31)	Postnatal care within 48 hours	17 countries in sub-Saharan Africa	To analyse the co–coverage of content interventions used as proxy for quality of care received by women during antenatal care and by the newborn during postnatal period using data from	Content coverage: Percentage of women with a live birth in last 2 years who were attended by a skilled birth attendant and received all 7 content interventions	Attended by a skilled birth attendant and received all 7 content interventions	Women with a surviving infant under 2 yrs	• DHS<br>• 2010 - 2015	n/a	Receipt of 7 interventions based on women's self-reports<br><br>*Binary: all components present vs. not*	None given	• Composite score<br>• Compares gap between contact and content coverage for each intervention separately and all 7 interventions combined.	Only able to include interventions available in HH survey across countries included. Measures based on mother's recall of care, may be subject to recall bias.

Reference	Intervention/service	Setting	Study aim(s)	Indicator	Numerator	Denominator	Data source(s)		Quality measure(s) (see Appendix D for individual items)	How quality measures derived	Results presented	Limitations of EC measure reported by author
							Household	Facility/provider				
			nationally representative surveys. To compare this co–coverage estimate with the global coverage indicators assessing contacts with health system to highlight the gap between contact and content.									
Hategeka et al. 2020 (52)	(1) Postpartum check-up before discharge<br><br>(2) Sick child care: treatment of suspected pneumonia<br><br>(3) Sick child care: treatment of diarrhoea<br><br>(4) Sick child care: treatment of fever	Rwanda	To assess effective coverage of MCH services in Rwanda, equity in effective coverage and its subnational distribution over the MDG era.	Effective coverage: Propn of individuals in need of MCH services who used the service and received quality MCH services	(1) Number women who delivered in a facility & were examined or asked about their health within 1 hour of delivery<br><br>(2) Number children received antibiotics when seeking care at a facility for symptoms of pneumonia<br><br>(3) Number children received ORT when seeking care at a facility for diarrhoea<br><br>(4) Number children tested for malaria	(1) Women aged 15-49 yrs with 1+ births in preceding 5yrs, whose most recent pregnancy lead to a live birth<br><br>(2) All children <5 who, in the past 2 weeks, suffered from symptoms consistent with pneumonia<br><br>(3) All children <5 who had diarrhoea in past 2 wks<br><br>(4) All children <5 yrs who had fever in the past 2 wks	• DHS<br>• 2010 & 2015	n/a	Two domains of processes of care: competent care (treatment & assessment), and system competence (timely care) based on women's self-reports<br><br>(1) Assessment & timely care: examined or asked about health before discharge & checked within 1 hour after giving birth.<br><br>(2-3) Treatment: received antibiotics/ORT<br><br>(4) Assessment: had blood taken from heel or finger for testing<br><br>*Binary: received or not*	• Lancet Global Health Commission on High-Quality Health Systems in the SDG era<br>International guidelines:<br>• WHO Safe Childbirth Check list<br>• Integrated Management of Childhood Illness (IMCI) guidelines	• Composite measure<br><br>• Compared national average change in crude & effective coverage between 2010 & 2015	Likely overestimate EC because: Quality measures include only a limited no. of recommended items. Dichotomous items (yes/no response) do not measure quality comprehensively. Other relevant indicators, such as appropriate assessment and diagnostic tests, timeliness of care and other preventive and curative treatments for each condition, are not available in the RDHS. Self-reported data; evidence that women's ability to accurately recall care received suggests poor for some indicators.
Joseph et al. 2020 (39)	Childbirth: post-delivery care	Malawi	To use health system and population information to define nutrition quality-adjusted coverage metrics and quantify their impact on breastfeeding and birthweight.	Quality-adjusted coverage: The proportion of deliveries in HF that received nutrition intervention.	Likelihood of receipt of interventions based on geographic area and delivery facility type: woman assigned the district average score by facility type based on reported place of care seeking.	Women with a live birth in the last 2 yrs	• MICS<br>• 2013-2014	• SPA<br>• 2013	Direct observations of 3 interventions received: breastfeeding initiation within 1 hour of delivery, skin-to-skin and rooming in.<br><br>*Average score across facility: equal weight given to each domain*	• None given	• Composite measure	Women linked based on reported source of care, so care might not be reflected of true quality experienced. Data sources not perfectly contemporaneous. Births captured up to 2 years before the survey period so predate SPA and quality of care might vary across this time period.

Reference	Intervention/service	Setting	Study aim(s)	Indicator	Numerator	Denominator	Data source(s)		Quality measure(s) (see Appendix D for individual items)	How quality measures derived	Results presented	Limitations of EC measure reported by author
							Household	Facility/provider				
Kanyangarara et al. 2018 (32)	Childbirth: obstetric service	17 LMIC: Bangladesh, Benin, Burkina Faso, DRC, Haiti, Kenya, Malawi, Mauritania, Namibia, Nepal, Rwanda, Senegal, Sierra Leone, Tanzania, Togo, Uganda, Zimbabwe	To assess obstetric service availability, readiness and coverage within and between 17 low- and middle-income countries.	Population-level coverage of obstetric services:<br>(1) The proportion of deliveries in HF with EmOC (basic or comprehensive) functionality (service availability)<br><br>(2) The proportion of deliveries in HF ready to provide obstetric services (facility readiness)	Number of deliveries occurring in each stratum (based on health facility type and managing authority), assigned average stratum score for:<br><br>(1) service availability<br><br>(2) facility readiness	Propn of recent live births	• DHS or MICS<br>• 2007 - 2015	• SARA or SPA<br>• 2007 - 2015	(1) Service availability: reported performance of 7 basic & 2 comprehensive signal functions<br><br>*Categorical: four levels of functionality based on number and type of signal functions performed*<br><br>(2) Facility readiness reported/observed availability of 23 items across 4-domains: (i) general requirements, (ii) staff & guidelines, (iii) equipment, (iv) medicines & commodities.<br><br>*Binary score: threshold (> 20 items present)*<br><br>Estimated indicators of service availability and readiness for each strata of health facility (based on health facility type and managing authority)	Systematic review:<br>• Gabrysch et al. 2012, New Signal Functions to Measure the Ability of Health Facilities to Provide Routine and Emergency Newborn Care<br>• Signal functions were excluded where not collected across all health facility surveys included in the analysis.<br>Classification of facility functionality based on international guidelines:<br>• WHO. Monitoring emergency obstetric care: a handbook. WHO: Geneva; 2009<br>• WHO's SARA theoretical framework	• Composite measure<br>• Comparison across countries<br>• Service availability & facility readiness adjusted coverage presented separately	Approach assumed that all the women who delivered in a health facility assigned to a specific stratum experienced the "average" service availability and readiness for that stratum.
Kemp et al. 2018 (47)	Childbirth: facility based delivery	Haiti	To explore facility readiness as a predictor of facility-based delivery in Haiti, controlling for other supply- and demand-side factors. Our challenge was to characterize readiness of delivery-related services, link that readiness to nearby births, and avoid the misclassification errors and strong assumptions made by previous studies.	Facility readiness: the level of delivery-related service readiness available and accessible to women living in each sampling cluster.	HF readiness for each health facility providing delivery services	All births occurring in 2 yrs preceding survey	• DHS<br>• 2012	• SPA<br>• 2013	Facility readiness, 52 binary indicators of general service readiness & 18 binary indicators of delivery-specific readiness based on SARA. No further details given<br><br>*Average score calculated at cluster level*	Based on the WHO's SARA theoretical framework.	• Composite measure	DHS birth data were collected before the SPA facility data; believe SPA offered plausible estimates of the service readiness environment surrounding births given that health facility readiness tends to be stable over a two-year timeframe.<br>Results rely on the completeness, consistency, and validity of the DHS and SPA datasets.<br>SPA used observation of equipment and

Reference	Intervention/service	Setting	Study aim(s)	Indicator	Numerator	Denominator	Data source(s)		Quality measure(s) (see Appendix D for individual items)	How quality measures derived	Results presented	Limitations of EC measure reported by author
							Household	Facility/provider				
												services rather than self-reported data, improving the validity of the readiness data. Composite score cannot discriminate between a facility adding latex gloves to its inventory from one acquiring electricity. Further work should test whether the service readiness factors measured by SPA actually align with the areas of quality that most drive demand for facility-based maternity services.
Koulidiati et al. 2018 (42)	Sick child care: treatment of illness	6 low performing regions in Burkina Faso	To estimate crude coverage and effective coverage of U5YO children in Burkina Faso focused on curative care provided by primary-level health facilities	Effective coverage: the propn of all children under 5 in need who actually sought care at a facility categorised as least high or intermediate performance quality.	Children sought care at a facility categorised as high or intermediate performance quality	All children under five that had an illness episode in the previous four weeks	• Household survey<br>• Oct 2013 - Feb 2014	• Facility survey for inventory<br>• Patient provider observation<br>• Vignette-based knowledge assessment<br>• Oct 2013 - Feb 2014	Three dimensions: (1) 9 process indicators based on observations related to performance of management of common childhood diseases and 2 related input indicators; (2) 11 process indicators based on vignettes related to theoretical management of severe childhood diseases and 7 related inputs; (3) general service readiness based on 5 structural indicators.<br><br>*Categorical: high/intermediate/low*<br>Based on facility or service-specific score. Facilities that met different criteria levels for each dimension, were assigned to the lower level	Based on the Donabedian framework & indices developed by Gouws et al. 2005, Measuring the quality of child health care at first-level facilities	• Composite measure; disaggregated by high performing facilities and both high & intermediate quality	Quality of care based on content of care and does not capture aspects such as patient adherence to treatment or health outcomes. Assumes every reported illness episode actually requires a medical care visit; might overestimate true need and therefore underestimate coverage.

Reference	Intervention/service	Setting	Study aim(s)	Indicator	Numerator	Denominator	Data source(s)		Quality measure(s) (see Appendix D for individual items)	How quality measures derived	Results presented	Limitations of EC measure reported by author
							Household	Facility/provider				
Larson et al. 2017 (53)	Childbirth: obstetric care	PHCs in 1 rural region in Tanzania	Linked population and facility data to assess the effective coverage of obstetric care for women in rural Tanzania, explore the bottlenecks in effective coverage and estimate wealth-based differences in receipt of effective care.	Effective coverage: the propn of women who delivered in facilities providing good care on successive dimensions of quality, beginning with basic infrastructure, followed by equipment and supplies, health worker knowledge and competence, provision of routine obstetric services and ending with provision of basic emergency obstetric care.	Women delivering at a facility receiving at least the minimum threshold of quality	All women ≥15 years who delivered a child in yr preceding interview.	• Household interviews<br>• Jan 2016 – April 2016	• Facility audits<br>• Jan 2016 – Feb 2016 (extracted data from facility register Jan-Dec 2015)<br>• Health worker interviews (incl. 2 clinical vignettes)<br>• Jan 2016 – April 2016	5 dimensions: (1) facility infrastructure; (2) availability of equipment, supplies & medicines; (3) HCW knowledge & competence (tested); (4) provision of routine obstetric services recorded in facility register; (5) provision of emergency obstetric and newborn services (BEmONC).<br><br>*Categorical: High/minimum/less than minimum*<br>Calculated the mean HCW knowledge score & mean input score for other 4 dimensions<br>High threshold = ≥90% of tracer indicators were complete or for the knowledge and skill dimension if the average health worker score was 80%<br>Minimum threshold = 50% completion of indicators.	Tracer indicators for equipment, supplies and medications were determined from the Tanzanian Ministry of Health required list, previously reported indices, and an expert review panel.	• • Quality cascade, 5 steps: infrastructure, equipment, HCW knowledge, provision of routine care, provision BEmONC	Thresholds for minimum quality have not been empirically defined, requiring somewhat subjective judgment of what constitutes adequate care. Threshold selected for minimum quality (50% completion) was permissive and thus represents the best-case scenario. Indicators for routine services were limited to those recorded in the facility registers. Conducted from facility-level data and may not reflect the actual experiences of each individual woman on the day of her visit.
Leslie et al. 2017 (33)	Sick child care: treatment of diarrhoea, fever or ARI	8 countries: Haiti, Kenya, Malawi, Namibia, Senegal, Rwanda, Tanzania & Uganda	To combine nationally representative facility and population survey data from eight countries to evaluate effective coverage of three primary care services at the subnational level.	Effective coverage: multiplied use of healthcare by average quality	Number of children under-5 who sought care from a formal provider. Each sick child was assigned the structural quality score for the reported category of source of care sought.	Children under 5 who had experienced diarrhoea, fever or acute respiratory illness in the prior 2 wks.	• DHS or MICS<br>• 2014 or 2015-2016	• SPA<br>• Uganda, Rwanda 2007;<br>• Namibia 2009;<br>• Kenya 2010;<br>• Haiti,<br>• Malawi 2013;<br>• Senegal 2013-14;<br>• Tanzania 2015	Technical quality based on observations of essential clinical actions. 4 domains: history taking, routine examination, drug administration & immunization, and client education & counselling<br><br>*Average score*<br>Calculated as the percent of actions completed out of items assessed per country.	International guidelines:<br>• WHO. Integrated Management of Childhood Illness: Chart Booklet. Geneva, Switzerland: WHO, 2014	• Composite measure<br><br>• EC calculated at sub-national and national level; compare between and within countries.	Data sources spanned 2007-2016, limiting contemporaneous cross-country comparisons. Facility-based estimates of healthcare quality may not fully reflect use patterns.

Reference	Intervention/service	Setting	Study aim(s)	Indicator	Numerator	Denominator	Data source(s)		Quality measure(s) (see Appendix D for individual items)	How quality measures derived	Results presented	Limitations of EC measure reported by author
							Household	Facility/provider				
Leslie et al. 2019 (37)	(1) Childbirth: delivery care<br><br>(2) Childbirth: newborn care<br><br>(3) sick child care: treatment of diarrhoea<br><br>(4) sick child care: treatment of respiratory illness	Mexico	To estimate effective coverage and its regional inequalities within IMSS based on routine health information and to identify the challenges in generating comprehensive estimates of health system performance.	Effective coverage: the number of individuals experiencing high-quality outcomes divided by the number in need of the service.	Number of individuals receiving care from an IMSS facility and experienced a positive health outcome	Population in of individuals with the symptom or condition requiring health service in strata by state and age group:<br><br>(1) delivery: Women with live birth in last year<br><br>(2) newborn: born alive in last year<br><br>(3) diarrhoea: parental report of child under 5 experiencing at least 3 days of diarrhoea or diarrhoea plus fever<br><br>(4) respiratory illness: child under 5 experiencing flu, cough, bronchitis, sore throat or pain in his/her ears in the past 2 weeks	• Mexican National Health and Nutrition Survey (ENSANUT)<br>• 2012	• IMSS Performance Indicators; based on health information systems.<br>• 2016	Health outcome:<br><br>(1) delivery: without complication or death (subtracting maternal complications and mortality from total cases).<br><br>(2) newborn: live births reaching 28 days without death due to respiratory infection, noso comial infection or sepsis<br><br>(3) diarrhoea: visits that did not result in hospitalisation due to diarrhoea<br><br>(4) respiratory illness: visits that did not result in hospitalization due to non chronic respiratory condition<br><br>As outcome not entirely amenable to health services and hence will occur even in the presence of a high-quality health system, rescaled effective coverage against a global benchmark.	None given	• Cascade, 3 steps: in need of services, using service, and experiencing high-quality outcomes	Assessment of quality using neonatal mortality without consideration of avoidable morbidity likely overestimated quality.
Leyvraz et al. 2016a (61)	Nutrition: Fortified Complementary Foods	Abidjan, Cote d'Ivoire	To determine the coverage of the Project de Promotion de l'Alimentation de Complément Enrichie du Jeune Enfant en Côte d'Ivoire (PACE) program among children 6–23 months of age living in Abidjan, identify the major barriers to coverage of the program, and formulate recommendations for future	Effective coverage: the proportion of children aged 6-23 months whose caregiver has fed the participating child the project-specific fortified complementary food (Farinor or Nutribon) at least once in the past 7 days	The number of children whose caregiver had heard of the project-specific fortified complementary food (Farinor or Nutribon) and had fed the child the product at least once in the last 7 days.	Number of children aged 0 to 23 mnths<br><br>(1) All children<br>(2) Children from poor households and with poor feeding practices	• Household survey<br>• September-October 2014	n/a	Receipt and timing of supplement based on caregiver's report<br><br>*Binary: taken as prescribed vs. not*	None given, adapted Tanahashi's model	• Coverage levels, 4 steps: message coverage, contact coverage, partial coverage & effective coverage	None given

Reference	Intervention/service	Setting	Study aim(s)	Indicator	Numerator	Denominator	Data source(s)		Quality measure(s) (see Appendix D for individual items)	How quality measures derived	Results presented	Limitations of EC measure reported by author
							Household	Facility/provider				
			program activities.									
Leyvraz et al. 2016b (62)	Nutrition: Fortified Complementary Foods	Telangana State, India	To determine the coverage of the fortified complementary food program managed by Andhra Pradesh Foods (AP Foods), a state government-owned food manufacturing company among children 6–35 months of age living in Telangana state, identify the major barriers to coverage of the program, and formulate recommendations for future program activities.	Effective coverage: the proportion of children that always consume the project-specific fortified complementary food (Bal Amrutham)	The number of children whose caregiver received the project-specific fortified complementary food (Bal Amrutham) and who always consumed the ration.	Number of children aged 0 to 35 mnths<br><br>(1) All children<br>(2) Children at risk of poverty and with poor feeding practices.	• Household survey<br>• November-December 2014	n/a	Receipt and timing of supplement based on caregiver's report<br><br>*Binary: taken as prescribed vs. not*	None given, adapted Tanahashi's model	• Coverage and utilisation levels, 4 steps: message coverage, contact coverage, partial coverage & effective coverage	None given
Leyvraz et al. 2018 (60)	Nutrition: Micronutrient powders	7 neighbourhoods of Nairobi County, Kenya	To determine the baseline MNP coverage and utilization mainly from existing free distribution through the government (i.e., to determine the coverage of the existing MNP program in the area), especially among subgroups that may be more vulnerable to inadequate nutrient intake as a result of poverty or poor IYCF practices.	Effective coverage: The proportion of children aged 6-23 months that consumed at least 3 sachets of the micronutrient powder in the last week	The number of children whose caregivers had ever heard of MNP and consumed at least 3 sachets of the MNP in the last week	Number of children aged 6 to 23 mnths<br><br>(1) All children<br>(2) Children at risk of poverty or poor feeding practices	• Household survey<br>• date n/r	n/a	Receipt and timing of supplement based on caregiver's report<br><br>*Binary: taken as prescribed vs. not*	None given, adapted Tanahashi's model	• Coverage processes, 4 steps: message coverage, contact coverage, partial coverage & effective coverage	The main limitation of this study was that the results were not representative of the country, or of all Nairobi. The neighbourhoods included in the survey were selected based on their inclusion in the project area of a new MNP program.

Reference	Intervention/service	Setting	Study aim(s)	Indicator	Numerator	Denominator	Data source(s)		Quality measure(s) (see Appendix D for individual items)	How quality measures derived	Results presented	Limitations of EC measure reported by author
							Household	Facility/provider				
Marchant et al. 2015 (34)	(1) Childbirth: Prevention of haemorrhage<br><br>(2) Postpartum check within 48 hours<br><br>(3) Postnatal check within 48 hours	4 regions in Ethiopia, 1 state in India, 1 state in Nigeria	To propose a measurement method for evaluating the quality of health care for mothers and newborns that links the coverage of each type of contact to the content of care that should take place during those contacts to estimate the coverage of high quality contacts at the population level.	High quality contacts:<br>(1) The percent of women who were attended at birth by a skilled birth attendant and received AMTSL<br><br>(2) The percent of women who had a post-partum check within 48 hours of birth and for whom all five post-partum processes were met - exclude this?<br><br>(3) The percent of newborns who had a post-natal check within 48 hours of birth and for whom all five post-natal processes were met	Contacts during which recommended set of processes for routine health care were met	(1-2) women aged 13-49 with a live birth in previous 12 mnths<br><br>(3) newborns born alive in previous 12 mnths	• Household survey<br>• 2012	• Facility survey<br>• Frontline worker survey: HCW who carried out last delivery recorded in maternity register<br>• 2012	Routine process of care based on HCW reports<br><br>(1) Attended at birth by a skilled birth attendant and HCW reported received AMTSL.<br><br>(2) Five post-partum processes<br><br>(3) Five post-natal processes<br><br>*Binary: all present vs. not*	International guidelines:<br>• Partnership for Maternal Newborn & Child Health and the Aga Khan University. Essential Interventions, Commodities and Guidelines for Reproductive, Maternal, Newborn and Child Health. A Global Review of the Key Interventions Related to Reproductive, Maternal Newborn and Child Health (RMNCH). 2011.	• Composite score<br>• EC measure presented at country or state level	SBA responses about their own behaviour at the last birth they attended. Birth attendants may be biased towards providing positive responses about their own behaviours, meaning that the method provides a 'most optimistic' estimate of coverage that can be applied in a standardised way.
Millar et al. 2014 (50)	Sick child care: Malaria	Bauchi and Sokoto States, Nigeria	To describe the current care-seeking and treatment pattern for children under five with fever in Northern Nigeria. Determine how many children with fever received treatment consistent with NNCP/WHO standards and which factors predict if a child under five with fever is taking to treatment.	Treatment pathway: Children under the age of five who sought prompt treatment at a provider and received it according to Nigeria National Malaria Control Program (NMCP)/WHO standards	Number of children who received ACT	Number of children aged 0-59 months that had malaria symptoms	• Household survey<br>• November - December 2012	n/a	Receipt and timeliness of treatment based on caregiver's report<br><br>*Binary: all components or not*	Prompt treatment based on recommendations from the RBM Partnership:<br>• RBM: Progress & Impact Series: Country Reports: Focus on Nigeria. Geneva: Roll Back Malaria Partnership; 2012.<br>Standard case management pathway based on international guidelines:<br>• WHO: Guidelines for the Treatment of Malaria. 2nd edition. Geneva: WHO; 2010.	• Treatment pathway, 4 steps: malaria symptoms, sought prompt treatment, received a blood test, received ACT	Relies on women's self-reports; including for blood test results.

Reference	Intervention/service	Setting	Study aim(s)	Indicator	Numerator	Denominator	Data source(s)		Quality measure(s) (see Appendix D for individual items)	How quality measures derived	Results presented	Limitations of EC measure reported by author
							Household	Facility/provider				
Mmanga et al. 2021 (59)	Vaccination: Full vaccination coverage	Malawi	To describe the trend in immunization coverage, dropout rates and effective immunization coverages (FVC) among children aged 12–23 months in Malawi.	Effective Immunization Coverage: proportion of children aged 12–23 month who received the recommended EPI vaccine antigens	Number of children fully vaccinated by 12–23 months according to the vaccination calendar timeline	Number of children under 12 years eligible for full vaccination	• DHS<br>• 2004, 2010, 2015-16	n/a	Children aged 12–23 months who received BCG, OPV3, Penta3, PCV3, Rota2 and MCV1 vaccines<br><br>*Binary: received or not*	Malawi Expanded Programme on Immunization (EPI) schedule	• Bottleneck analysis framework, 4 steps: initial utilization (received either BCG or Penta1), continuous utilization (received Penta 3), adequate coverage (received MR1), full vaccination coverage (BCG, OPV3, Penta3, PCV3, RV2 and MCV1)	DHS does not have data on supply determinants of services (commodities, human resources and geographic access) so only able to focus on demand and quality determinants of services.
Mokdad et al. 2015 (35)<br>*Salud Mesoamérica	Vaccination: MMR	Poorest quintile of the population in 6 countries: El Salvador, Guatemala, Honduras, Mexico, Nicaragua, and Panama.	To assess the presence of missed opportunities to vaccinate using a large household survey in Mesoamerica. To estimate the potential increase in immunization coverage and reduction in days at risk if every opportunity to vaccinate a child was used, they analysed vaccination histories of children 11–59 months of age from large household surveys in Mesoamerica.	Missed opportunity:<br>(1) Timely MMR coverage according to card only considering timeliness<br><br>(2) MMR coverage among children attending facilities with MMR in stock on day of survey<br><br>(3) MMR coverage among children attending facilities with MMR stock-out in 3 mnths prior to the survey<br><br>(4) MMR coverage among children attending facilities with ORS in stock on day of survey	(1) Children aged 13 months or older with a MMR vaccine given between 11.5 and 13.5 months<br><br>(2) Children with required number of doses for age with proper time interval and not before eligibility window for MMR coverage attending facilities with MMR in stock on day of health facility survey<br><br>3) Children with required number of doses for age with proper time interval and not before eligibility window for MMR out of stock in three months prior to health facility survey;<br><br>(4) Children with required number of doses for age with proper time interval and not before eligibility	Number of children aged 11-59 months with a vaccine card	• Household survey, incl. review of vaccination card<br>• March 2011 - August 2013.	• SM2015 baseline health facility survey	(1) Timely vaccination as recorded on card: vaccine administered between 11 and 13 months<br><br>*Binary: timely or not*<br><br>(2-4) Facility readiness: Availability of MMR vaccine and oral rehydration salts<br><br>*Binary: present or not*	National guidelines: MMR vaccination required at 12 months in all countries in Mesoamerica<br>No details given on other components	• (1) Coverage cascade, 3 steps: owning a health card, vaccine coverage and timeliness<br>• (2, 3 & 4) Composite score	Calculated missed opportunities using vaccination visits only as data on other health care visits not available. Using all visits as possibilities for vaccination would results in increased coverage.

Reference	Intervention/service	Setting	Study aim(s)	Indicator	Numerator	Denominator	Data source(s)		Quality measure(s) (see Appendix D for individual items)	How quality measures derived	Results presented	Limitations of EC measure reported by author
							Household	Facility/provider				
					window for MMR coverage attending facilities with ORS in stock on day of survey							
Munos et al. 2018 (43)	(1) Childbirth: Labour & delivery<br><br>(2) Childbirth: immediate newborn care<br><br>(3) post-discharge postnatal care for mother and baby within 2 days of birth<br><br>(4) sick child care: treatment fever, cough or diarrhoea	Savanes region of Côte d'Ivoire	To better understand the feasibility and comparability of exact-match and different ecological methods for linking household and health provider surveys to obtain effective coverage measures.	(1) Structure-adjusted coverage: proportion of women or children visiting a facility that is ready to provide care<br><br>(2) Process-adjusted coverage: proportion of women or children visiting a facility that provides actual processes of care	Average structural or process quality scores for the provider category reported as the source of care.	(1-3) Women reported a live birth in the 2 yrs preceding the survey<br><br>(4) Mothers of children under-5 years who reported child had fever, cough or diarrhoea in 2 weeks before survey	• MICS; 6 Qs added to identify exact sources of care.<br>• May – July 2016	• Facility inventory adapted from SPA & SARA.<br>• Caseload from facility registers.<br>• HCW survey<br>• Observations of postnatal and sick child consultation<br>• May-June 2016	Three domains of structural quality: (1) service availability; (2) availability of drugs, diagnostics & commodities; (3) training, supervision & availability of guidelines.<br><br>Process quality based on observed processes of care, including the patient's activities, the provider's activities, and the interactions between the two.<br><br>*Average score*<br>Number of items present divided by total number of items. Score ranged from 0 to 1.<br>Training variable: proportion of HCW at facility who had received training.	Used Donabedian definitions of structural quality and process quality.<br>SARA analysis guide for structural quality.<br>Process quality used international guidelines:<br>• WHO Safe Childbirth Checklist.<br>• WHO Recommendations on Postnatal Care of the Mother and Newborn. WHO Guidelines Approved by the Guidelines Review Committee. Geneva: WHO; 2013<br>• WHO. Integrated Management of Childhood Illness. Chart Booklet. Geneva: WHO; 2014	• Composite measure<br>• Structure & process adjusted coverage presented separately<br>• EC estimates presented by different linking approach.	Relies on women reporting the source of are visited; although errors likely to be rare. Assumes no error in our measurement of facility quality because collected data from all facilities.

Reference	Intervention/service	Setting	Study aim(s)	Indicator	Numerator	Denominator	Data source(s)		Quality measure(s) (see Appendix D for individual items)	How quality measures derived	Results presented	Limitations of EC measure reported by author
							Household	Facility/provider				
Murphy et al. 2018 (38)<br>* Nairobi Newborn Study	Inpatient neonatal care	Nairobi City County, Kenya	To report on the quality of the process of care delivered to small and sick inpatient newborns across diverse facility settings in Nairobi with a view to understanding effective coverage.	Effective coverage: Proportion of newborns needing care attending a facility providing high-quality care	Number of neonates attending a facility providing high quality care	Number of newborns requiring care; estimated by applying the rate of live births requiring inpatient services (183 per 1000 live births) to the total number of live births in the study region between mid-2014 to mid-2015	None:<br>• Estimated number of live births in Nairobi City County in 2017 by applying the Nairobi City County crude birth rate obtained from the Kenyan 2014 demographic and health survey to population estimates for the County, derived from the 2009 national census and adjusted for population growth from 2009 to 2017.	• Facility assessment<br>• Neonatal medical records review<br>• July 2014 - June 2015	Process quality based on medical records across 6 domains: (i) documentation of newborn characteristics, (ii) documentation of signs and symptoms, (iii) evidence of monitoring, (iv) correct antibiotic dose, (v) correct oxygen treatment and (vi) correct fluids and feeds prescribed.<br><br>Structural quality across 8 domains: (i) infrastructure, (ii) laboratory services, (iii) hygiene equipment, (iv) safe delivery equipment and drugs for mothers, (v) resuscitation equipment for newborns on the delivery ward, (vi) essential equipment in the newborn unit (NBU), (vii) intravenous fluids and feeds in the NBU and (viii) NBU drugs.<br><br>*Categorical: high/medium/low*<br>Average process quality >60% & structure score >=80% considered high quality	Dosage of antibiotics or fluids and feeds based on national guidelines.<br>No details given on other components	• Composite measure; disaggregated by low, medium & high quality	Use of medical records limits assessment to indicators that are routinely documented.<br>Medical records are not standardised across facilities, may have contributed to lower performance in non-public-sector facilities.<br>Quality of care defined based on national standards of care; may not apply to private sector.<br>Not able to assess mortality.
Nesbitt et al. 2013 (54)<br>*Newhints trial	Childbirth: Intrapartum and immediate newborn care	7 districts in Brong Ahafo, Ghana	To evaluate quality of routine and emergency intrapartum and postnatal care using a health facility assessment, and to estimate "effective coverage" of skilled attendance.	Effective coverage: proportion of births in facilities of high quality	Delivery in a facility with "high" or "highest" quality in all four dimensions	Live births with known birthplace	• Surveillance data of all women of child bearing age in the Newhints trial area<br>• Nov 2008 – Dec 2009	• Health facility assessment<br>• Oct-Nov 2010	Signal functions and corresponding drugs & equipment across, 4 dimensions: (1) routine delivery care, including labour and immediate postnatal care, (2) emergency obstetric care (EmOC), (3) emergency newborn care (EmNC), and	Based on functions included in other large-scale facility assessments in consultation with local clinicians:<br>• Gabrysch et al. 2012, New Signal Functions to Measure the Ability of	• Composite measure<br>• Estimates coverage & quality gap between coverage of facility delivery and provision of high quality care. Presented for each quality dimension separately and all 4 dimensions combined.	Relied on reported performance of signal functions as did not observe provision of care.<br>May have overestimated quality of functions that did not validate with tracer items.

Reference	Intervention/service	Setting	Study aim(s)	Indicator	Numerator	Denominator	Data source(s)		Quality measure(s) (see Appendix D for individual items)	How quality measures derived	Results presented	Limitations of EC measure reported by author
							Household	Facility/provider				
									(4) non-medical quality.<br><br>*Categorical: highest/ basic/ intermediate/ low/ lowest*<br>Calculated based on reported availability of drugs & equipment, reported frequency of performance of signal functions, number of trained health professionals & capacity for referral.	Health Facilities to Provide Routine and Emergency Newborn Care		
Nguhiu et al. 2017 (40)	(1) Childbirth: skilled delivery & perinatal care<br><br>(2) Vaccination: complete set of basic vaccines<br><br>(3) Sick child care: care seeking for acute respiratory illness/fever<br><br>(4) Exclusive breastfeeding during first 6 months of life<br><br>(5) Sick child care: management of diarrhoea<br><br>(6) Insecticide treated nets	Kenya	To estimate the levels of and inequities in EC of maternal and child health (MCH) services in Kenya, as a means of tracking the country's progress towards UHC.	Effective coverage:<br>(1, 2 & 3) Propn of individuals in need of intervention who attended a health facility that was ready to provide care.<br><br>(4, 5 & 6) Propn of individuals in need of intervention who reported receipt of recommended components of care.<br><br>Overall EC estimate calculated as the average of 8 intervention-specific EC<br>* Note data on 2 interventions (family planning and ANC) not extracted	(1) Women attended by a SBA at most recent birth adjusted for facility quality<br><br>(2) All children who received complete set of vaccines adjusted for facility quality<br><br>(3) All children who sought advice from a medical provider adjusted for facility quality<br><br>(4) All children exclusively breastfed within the last 24hrs.<br><br>(5) All children given ORT or increased fluids<br><br>(6) All pregnant women & children who lived in a house with an ITN and slept under the net the previous night	(1) All women 15–49 yrs old with at least one child under 5 yrs<br><br>(2) All children alive aged 12-23 mnths<br><br>(3) All children under 5 yrs reported to have had acute respiratory illness and/or fever in preceding 2 wks<br><br>(4) All children aged 0-5 mths<br><br>(5) All children under 5 yrs reported to have had diarrhoea in preceding 4 wks<br><br>(6) All children (age NR assume under 5 yrs)	• DHS<br>• 2003, 2008-2009, 2014	• Interventions 1-3 only:<br>• SPA<br>• 2004, 2010	(1) HCW reports of 9 items incl. structural features, essential newborn care practices and administration of key medicines<br><br>(2 & 3) quality of primary care for children based on observed or HCW reports for 7 items incl. structural features and processes of care<br><br>*Average score*<br>Calculated at the provincial level<br><br>(4, 5 & 6) Receipt of intervention based on respondent's report.<br><br>*Average score*	None given	• Composite measure<br>• Presents change in EC from 2003 to 2014	Few other reliable national data sources in Kenya other than DHS (MICS only conducted in selected regions and not representative at country level)<br>Good estimates of quality of care requires information from different datasets; not available for all interventions. For those interventions where available in KSPA quality measure was applied equally to all individuals.

Reference	Intervention/service	Setting	Study aim(s)	Indicator	Numerator	Denominator	Data source(s)		Quality measure(s) (see Appendix D for individual items)	How quality measures derived	Results presented	Limitations of EC measure reported by author
							Household	Facility/provider				
Nguyen et al. 2021 (44)	(1) Childbirth: birth care<br><br>(2) Child Growth Monitoring<br><br>(3) Sick child care: nutrition education, vit A supplementation, deworming, anaemia management ORS and zinc for diarrhoea	Bangladesh	To adjust contact-based health coverage estimates in Bangladesh, taking into consideration the inputs required to deliver quality nutrition interventions across the continuum of care, specifically ANC and delivery for women and growth monitoring and curative care for young children.	Input-adjusted coverage: the proportion of women/children who sought care at a facility accounting for the type of facility where care was sought<br><br>* defines effective coverage using Marsh seven-step coverage framework	(1) Women 15–49 years old with at least one child under 5, whom for their most recent birth, reported delivery in a health facility<br><br>(2 -3) All children who had diarrhoea or ARI symptoms for whom care was sought from a medical provider	(1) Women 15–49 years old with at least one child under 5<br><br>(2) All children alive between 0 and 59 months;<br><br>(3) All children alive between 0 and 59 months who had diarrhoea or ARI in the last 2 weeks	• DHS<br>• 2014	• SPA<br>• 2014	Facility readiness, based on 5 attributes guided by SARA: (1) trained personnel, (2) guidelines, (3) equipment, (4) diagnostic capacity and (5) medicines.<br><br>*Average score from 0 to 100*<br>Calculated at facility-level and disaggregating by region and urban/rural location Five domains weighted equally within each measure.	Based on the WHO's SARA theoretical framework.	• Composite measure	Contact coverage based on woman's recall. Underestimate contact coverage of growth monitoring services if well children are frequently brought to health facilities for this purpose. SPA does not capture every aspect to measure facility readiness to provide nutrition interventions (such as the infrastructure to implement kangaroo mother care, calcium or food supplements for pregnant women, or IFA and food supplements for children). Data do not allow calculation of care cascade.
Nguyen et al. 2016 (57)	Nutrition: Complementary foods with micronutrient powders	4 provinces in Vietnam (Hai Phong, Thai Nguyen, Quang Nam, and Ca Mau in the South)	To present the pilot's design, implementation, coverage results, and MNP use and compliance by caregivers. To provide recommendations on how the results from this pilot could help inform the strategy on home fortification of complementary foods with MNPs for micronutrient deficiency prevention, and how this model could be scaled up in Vietnam.	Effective coverage: the proportion of children that consumed at least 3 sachets of the National Institute of Nutrition-produced micronutrient powder in the last week	Number of children aged 6-59 mnths that consumed at least 3 sachets of the National Institute of Nutrition-produced micronutrient powder in the last one week	Number of children aged 6-59 mnths	• Household survey<br>• November - December 2014	n/a	Receipt and timing of supplement based on caregiver's report<br><br>*Binary: taken as prescribed vs. not*	Consumption per week based on WHO recommendations and the Home Fortification Technical Advisory Group Programmatic Brief:<br>• WHO. Guidelines: Use of Multiple Micronutrients Powders for Home Fortification of Foods Consumed by Infants and Children 6–23 Months of Age. 2011.<br>• HF-TAG. Programmatic Guidance Brief on Use of Micronutrient Powders (MNP) for Home Fortification.	• Coverage level, 4 steps: message coverage, contact coverage, partial coverage & effective coverage	Data was collected from caregivers at health centres which could have biased responses. Used one-week recall period to avoid recall bias. Questionnaire was developed to have harmonized questions for countries implementing MNP programs globally, some relevant indicators that would have helped better understand caregivers' behaviours/purchase trends/use of Bibomix in Vietnam were missing.

Reference	Intervention/service	Setting	Study aim(s)	Indicator	Numerator	Denominator	Data source(s)		Quality measure(s) (see Appendix D for individual items)	How quality measures derived	Results presented	Limitations of EC measure reported by author
							Household	Facility/provider				
Okawa et al. 2019a (49)	(1) Childbirth: peripartum care<br><br>(2) postnatal care for mother & newborn	1 urban and 1 rural region, Myanmar	To assess the contact of women and their newborns with healthcare providers, quality of care and quality-adjusted contacts during ANC, delivery and PNC, and to identify factors associated with having adequate contact and receiving high-quality care in Myanmar.	Quality-adjusted contact: having adequate contact and receiving high-quality care.	(1) Attended by skilled care providers at a health care facility and receiving high-quality care.<br><br>(2) At least 3 contacts for PNC, with first contact within 24 hrs postpartum and receiving high-quality care	All women 6 wks to 12 mnths postpartum	• Household survey<br>• March 2016	n/a	Content care based on women's self-reports.<br><br>(1) 7 interventions for delivery<br><br>*Binary: all items present vs. not*<br><br>(2) 17 interventions for PNC (12 maternal and 5 newborn)<br><br>*Binary: threshold (highest quality vs. rest)*<br>High-quality: top 20 percentile	Domain of competent care defined in the high-quality health system framework:<br>• Kruk et al. 2018 High-quality health systems in the Sustainable Development Goals era: time for a revolution.<br>National guidelines:<br>• Ministry of Health and Sports. Maternal and Child Health Handbook. Nay Pyi Taw, Myanmar: Ministry of Health and Sports, Unknown<br>International guidelines:<br>• WHO. Pregnancy, childbirth, postpartum and newborn care: a guide for essential practice. 3rd edn. Geneva, Switzerland: WHO, 2015.	• Composite measure<br>.	Relies on women's recall on having received interventions.<br>Only assessed one aspect of quality of care.
Okawa et al. 2019b (46)<br>*EMBRACE trial	(1) Childbirth: peripartum care<br><br>(2) postnatal care for mother & newborn	3 rural sites, Ghana	To examine the effects of the continuum of care intervention package on adequate contacts with healthcare providers and high-quality care by the mothers and their newborns compared with the standard maternal and newborn care under the national guidelines and to determine the	Adequate contacts with high quality care:<br>Proportion of women who received adequate contacts with a healthcare provider and high-quality care.	(1) Skilled facility based delivery and received all components of care<br><br>(2) 3 timely contacts and received all components of care	Women aged 15-49 yrs and delivered in 2 yrs prior to survey	• Household survey<br>• July-Sept 2014 & Oct-Dec 2015	n/a	Content of care based on women's self-reports<br><br>(1) Three care items<br><br>(2) Based on the number and timing of contacts and receipt of 14 care items<br><br>*Categorical: Inadequate contact regardless of quality, adequate contact with low or high quality care*<br>Low quality:<br>≤13 care items	Process-of-care dimension in Donabedian's framework	• Composite measure.<br>• Presents 3 outcomes: inadequate contact (≤2 contacts or non-timely contacts), adequate contact with low quality care and adequate contact with high quality care	No standardised measurement of quality available. Although value of each item not equal gave them equal weight.

Reference	Intervention/service	Setting	Study aim(s)	Indicator	Numerator	Denominator	Data source(s)		Quality measure(s) (see Appendix D for individual items)	How quality measures derived	Results presented	Limitations of EC measure reported by author
							Household	Facility/provider				
			factors associated with having adequate contacts with high-quality care.						High-quality: All care items received			
Sharma et al. 2017 (41)	Childbirth: delivery care	Kenya	To assess whether high quality maternal care is equitably distributed by (1) mapping the quality of maternal care in facilities located in poorer versus wealthier areas of Kenya; and (2) comparing the quality of maternal care	Population access to quality care: percentage of the population with access to minimally adequate standard of maternal care.<br><br>(1) quality of maternal health care infrastructure<br><br>(2) quality of delivery care	(1) Population living within 5-km radius of a facility with adequate maternal care infrastructure<br><br>(2) Population living within 5-km radius of a facility with adequate delivery quality	Total population	• DHS<br>• Oxford Poverty & Human Development Initiative<br>• 2010	• SPA<br>• 2014	Structural inputs (infrastructure, staffing & equipment) and clinical care processes.<br><br>*Binary: threshold (adequate maternal care quality <0.75)*<br>Averaged to provide a facility-level score from 0 to 1.	Using Donabedian's framework. Applied the quality of the process of intrapartum and immediate postpartum care (QoPIIPC) metric validated by Tripathi et al. 2015 [Development and Validation of an Index to Measure the Quality of Facility-Based Labor and Delivery Care Processes in Sub-Saharan Africa]	• Composite measure<br>• Infrastructure & quality adjusted coverage presented separately	Small number of observations; difficult to obtain multiple observations for low-volume facilities.<br>Lack of universally defined minimum quality standards, selected 0.75 threshold on premise that women should receive most basic items.
Sheff et al 2020 (48)<br>* CHPS+ project	Vaccination: Complete set of basic vaccines	7 districts in Volta Region, Ghana	Using a modified version of the 1978 Tanahashi model as an analytical framework, aims to examine the system of care at the community level in Ghana's Volta Region to highlight the continued reforms needed to achieve UHC.	Quality coverage: the proportion of children who have received all vaccines mandated by Ghana's Expanded Programme on Immunisation by 24 months<br><br>* Two additional coverage measures calculated separately and are not included in extraction<br><br>(1) Availability coverage: the proportion of facilities with all health commodities and human	Number of children that received all vaccines on time	Number of children aged 12-23 months	• Household survey incl. review of vaccination card<br>• April to October 2017	• Health facility assessment<br>• July 2018<br><br>* used to calculate availability coverage	Receipt of complete package of vaccine on time: one dose of BCG at birth, three doses of the oral polio vaccine (excluding the dose given at birth), three doses of a DPT containing vaccine and hepatitis B vaccine at 6, 10, and 14 weeks, and one dose of the measles vaccine, all done by 24 months.<br><br>*Binary: received or not*	Mandated by Ghana's Expanded Programme on Immunization (EPI)	• Modified Tanahashi model, 5 steps: (1) availability of health services & human resources, (2) geographic & financial accessibility, (3) initial contact, (4) continued utilisation, (5) quality coverage.<br><br>*Calculates two measures: (1) potential coverage, which includes availability and accessibility, calculated at the facility level, (2) actual coverage combining initial contact, continued utilisation and quality coverage, calculated at the population level.	Survey instruments not designed specifically for the Tanahashi model; missing more specific information such as vaccine availability by antigen. Range of data sources needed to develop these models may not be widely available.

Reference	Intervention/servic e	Setting	Study aim(s)	Indicator	Numerator	Denominator	Data source(s)		Quality measure(s) (see Appendix D for individual items)	How quality measures derived	Results presented	Limitations of EC measure reported by author
							Household	Facility/provi der				
				resources available<br><br>(2) Accessibility coverage: the proportion of women who have a valid NHIS card and the proportion of women living within 5km of a facility								
Shibanuma et al. 2018 (58)<br>* EMBRACE trial	(1) Childbirth: facility delivery<br><br>(2) PNC within 48 hrs and around 2 and 6 weeks post-delivery	3 rural sites, Ghana	To compare continuum of care achievement in MNCH based on two measurements: (1) visits and (2) key components of services that were received. To compare the factors affecting continuum of care based on the two different measurements. Finally, to examine whether achievements differed across areas.	Continuum of Care achievement: Proportion of women and children who received MNCH services at delivery and post-delivery stages and who received the key components of MNCH services	(1) delivered at a health facility and received all components of care<br><br>(2) both mother and newborn received timely PNC and all components of care	Women aged 15-49 yrs who had a live birth or stillbirth in 2 yr prior to survey.	• Household survey<br>• July 2013	n/a	Receipt of key components of care based on women's self-reports<br><br>*Binary: received all components or not*	International guidelines:<br>• The Partnership for Maternal, Newborn & Child Health. A global review of the key interventions related to reproductive, maternal, newborn and child health (RMNCH). Geneva, Switzerland: PMNCH, 2011<br>• WHO. Guidelines on maternal, newborn, child and adolescent health approved by the WHO guidelines review committee: Recommendations on maternal and perinatal health. Geneva, Switzerland: WHO, 2013<br>• United Nations Children's Fund. Committing to child survival: A promise renewed. New	• Composite measure	Visits and receipt of care measured based on women's self-reports; included only key components that could be ascertained in interviews with women.

Reference	Intervention/service	Setting	Study aim(s)	Indicator	Numerator	Denominator	Data source(s)		Quality measure(s) (see Appendix D for individual items)	How quality measures derived	Results presented	Limitations of EC measure reported by author
							Household	Facility/provider				
										York, NY, USA: United Nations Children's Fund, 2014.<br>• WHO. Pregnancy, childbirth, postpartum, and newborn care: A guide for essential practice. Geneva, Switzerland: WHO, 2006<br><br>Published literature:<br>• Singh et al. 2014 Postnatal care by provider type and neonatal death in sub-Saharan Africa: a multilevel analysis<br>• Adegoke et al. 2009 Skilled birth attendance- lessons learnt<br><br>Comments from health administrators at the study site.		
Smith et al. 2010 (51)	Sick child care: Malaria	3 districts (Tambacounda, Koumpentoum and Maka Coulibantang), Senegal	To analyse the application of a diagnostic approach to the coverage of prompt and effective treatment for febrile children in rural Senegal, assessing the critical steps at which children exit from the treatment pathway, stratified according to source of first advice or treatment.	Treatment pathway: Proportion of children under the age of five that received artesunate-amodiaquine (AS-AQ)	The number of children under the age of five that sought care within 48 hours and that received AS-AQ	Number of children aged under five years.	• Household survey<br>• August-September 2008	n/a	Prompt and effective treatment based on self-reports<br><br>*Binary: received or not*	Based on the National Malaria Control Programme, which specifies first line anti-malarial, and the Roll Back Malaria (RBM) access indicator.	• Treatment pathway, 5 steps: fever in the previous two weeks, sought any advice/treatment, care sought within 48 hours, received any anti-malarial, received an ACT.	• Limited sample size that led to wide confidence intervals;<br>• Expectations of inaccuracies in the drugs provided given it is based on recall but this was minimized with the presentation of a photo during the interview;

Reference	Intervention/service	Setting	Study aim(s)	Indicator	Numerator	Denominator	Data source(s)		Quality measure(s) (see Appendix D for individual items)	How quality measures derived	Results presented	Limitations of EC measure reported by author
							Household	Facility/provider				
Wang et al. 2019 (36)	Childbirth: facility delivery	6 countries: Bangladesh, Haiti, Malawi, Nepal, Senegal, and Tanzania	To estimate the effective coverage of obstetric and newborn care with a refined approach. This method takes into account different types of facilities where women delivered their births. We also estimated the uncertainty of the effective coverage estimates, which has not been commonly done.	Effective coverage: Calculated among individuals in need of care as the mathematical product of the use of the service and the quality of care provided.	Facility readiness for the type of facility where delivery care was sought.	Number of births in the 2 yrs preceding the survey	• DHS<br>• Bangladesh: 2014,<br>• Haiti: 2012,<br>• Malawi: 2015-2016,<br>• Nepal: 2016,<br>• Senegal: 2016,<br>• Tanzania: 2015-16	• SPA<br>• Bangladesh: 2014,<br>• Haiti: 2013,<br>• Malawi: 2013-14,<br>• Nepal: 2015,<br>• Senegal: 2015,<br>• Tanzania: 2014-15	Facility readiness, 6 domains: (1) comprehensive EOC, (2) newborn signal functions, (3) infrastructure, (4) equipment, (5) supplies & commodities, (6) the availability of guidelines trained personnel.<br><br>*Average score: equal weight approach*<br>Equal weight given to 6 domains and to all indicators within the same domain; sum of all domains standardised to have a maximum of 100.	International guidelines:<br>• WHO. Service Availability and Readiness Assessment (SARA): An annual monitoring system for service delivery Reference Manual. Geneva, Switzerland: WHO; 2015.<br>• Save the Children Federation I. Newborn indicators 2017<br>Systematic review:<br>• Gabrysch et al. 2012, New Signal Functions to Measure the Ability of Health Facilities to Provide Routine and Emergency Newborn Care	• Composite measure	The readiness score itself cannot identify specific deficits. Facilities with a similar score could possess quite different specific tracer items.
Willey et al. 2018 (56) *EQUIP study	Childbirth: Basic emergency obstetric care	1 district in Uganda	To explore methods for linking access to skilled birth attendance (SBA) from household surveys to data on provision of care from facility surveys with the aim of estimating population level effective coverage reflecting access to quality care.	Effective coverage of skilled birth attendance in facilities ready to provide basic emergency obstetric and newborn care.	Product of prevalence of attendance by an SBA in a health facility and the prevalence of facility readiness	(1-2) women aged 13-49 with a live birth in previous 12 mnths<br><br>(3) newborns born alive in previous 12 mnths (12	• HH survey<br>• Jan 2012 – Dec 2013	• Facility survey<br>• Nov 2012 – Feb 2013	Facility readiness, 6 components: (1) infrastructure, (2) infection prevention, (3) commodities to monitor and manage labour, (4) essential medicines, (5) commodities to provide neonatal resuscitation, (6) commodities to provide clean cord care.<br><br>*Binary: all commodities for all 6 components available vs. not*	Systematic review:<br>• Gabrysch et al. 2012, New Signal Functions to Measure the Ability of Health Facilities to Provide Routine and Emergency Newborn Care	• Composite measure<br>• EC estimates presented by different linking approach.	Relied on women's self-report of skilled birth attendance, which is susceptible to measurement error.<br>Facility readiness surveys represent availability on the day of survey; analysis revealed that some but not all commodities were stable over time.<br>Quality measure focused on commodities; did not incorporate availability, training or capability of health facility staff attending births and caring for newborns, nor estimates of coverage of actual life-saving behaviours. EC

Reference	Intervention/servic e	Setting	Study aim(s)	Indicator	Numerator	Denominator	Data source(s)		Quality measure(s) (see Appendix D for individual items)	How quality measures derived	Results presented	Limitations of EC measure reported by author
							Household	Facility/provi der				
												measure represents capacity to deliver quality care, rather than the quality of care delivered in practice.

APPENDIX S4 – FULL MAPPING OF INCLUDED EFFECTIVE COVERAGE MEASURES AGAINST STEPS OF THE COVERAGE CASCADE

Table S4 Childbirth and immediate newborn care

**Study (data sources)**	**Baker et al. 2015 (55) (HH survey; HF assessment; HCW interview)**		**Joseph et al. 2020 (39) (MICS; SPA)**	**Kanyangarara et al. 2018 (32) (DHS; MICS; SARA; SPA)**		**Kemp et al. 2018 (47)* (DHS; SPA).**	**Larson et al. 2017 (53) (HH survey; HF assessment; HCW interview)**	**Leslie et al. 2019 (37) (ENSANUT [national survey]; HMIS)**		**Marchant et al. 2015 (34) (HH survey; HF assessment; HCW interview)**	**Munos et al. 2018 (43) (MICS; HF assessment; HCW interview)**			
**Health service/intervention**	**(1) Partograph to monitor labour**	**(2) Active management of third stage of labour**	**Post-delivery care**	**(1) Obstetric services: readiness**	**(2) Obstetric services: service availability**	**Facility based delivery**	**Obstetric care**	**(1) Delivery care**	**(2) Immediate newborn care**	**Prevention of PPH**	**(1) Labour & delivery: structural quality**	**(2) Labour & delivery: process quality**	**(3) Immediate newborn: structural quality**	**(4) Immediate newborn: process quality**
**Target population**														
Women who have given birth	Women with live birth in 12mnths prior to survey	Women with live birth in 12mnths prior to survey	Women with a live birth in last 2 yrs	Proportion of recent live births	Proportion of recent live births	Women living in the catchment area of one of the study dispensaries, who were at least 15 years of age and had delivered within 1 yr prior to interview	Women living in the catchment area of one of the study dispensaries, who were at least 15 years of age and had delivered within 1 yr prior to interview	Proportion of women per state with a past-year live birth for each 5-year age group from 20 to 50	Proportion of women per state with a past-year live birth for each 5-year age group from 20 to 50	Women aged 13-49 who had a live birth in 12 mnths prior to survey	Women report a live birth in the 2 yrs preceding the survey	Women report a live birth in the 2 yrs preceding the survey	Women report a live birth in the 2 yrs preceding the survey	Women report a live birth in the 2 yrs preceding the survey
**Service contact coverage**														
Facility based childbirth	Women reported giving birth in a facility	Women reported giving birth in a facility	Women reported delivered in a health facility	Recent live facility births	Recent live facility births	Women reported delivered in a health facility	Facility based births recorded in facility record	Deliveries in IMSS facilities	Live newborns in IMSS facilities	Women reported institutional delivery & attended by a skilled birth attendant	Women report delivery in a health facility	Women report delivery in a health facility	Women report delivery in a health facility	Women report delivery in a health facility
**Input-adjusted coverage**														
**Inputs: service infrastructure**														
Ambulance/Emergency transport					Reported availability and reported functionality of a vehicle with fuel that is routinely available that can be used for emergency transportation or access to a vehicle in near proximity that can be used for emergency transportation	Reported availability: Facility has a functioning vehicle with fuel that is routinely available that can be used for emergency transportation or access to a vehicle in near proximity that can be used for emergency transportation					Facility reported to have a functioning vehicle with fuel that is routinely available that can be used for emergency transportation or access to a vehicle in near proximity that can be used for emergency transportation			
Communication equipment					Observed availability and reported functionality of a shortwave	Reported availability: Functioning communicatio n equipment. This will not								

					radio or phone (landline or cellular)	include private cell phones unless the facility reimburses for cost of phone calls. This will not include payphones outside of the facility.								
Computer with email/internet access						Reported availability: Facility has a functioning computer and has access to email/internet with internet working on the day of the survey.								
Delivery beds					Observed availability of a delivery bed		Delivery table				Delivery bed			
Examination light					Observed availability and reported functionality of a spotlight source (or flashlight)	Items observed and functioning in the main service area: Spotlight source that can be used for patient examinations. A functional flashlight is accepted.	Examination lamp				Observed a functioning spotlight source that can be used for patient examinations in service area or adjacent area. A functional flashlight is accepted.			
Facility register														
Patient toilet clean & water & soap for handwashing														
Power/Electricity					Reported availability of electricity for lights and communication (at a minimum) from any power source, with no break in power for more than 2 h per day during the past 7 d	Reported availability: Facility routinely has electricity for lights and communication (at a minimum) from any power source during normal working hours; there has not been a break in power for more than 2 hours per day during the past 7 days	Electricity							

Private delivery room						Observed availability: Private room or screened off area available in main service area (usually the general outpatient service area), a sufficient distance from sites where providers/clients routinely may be, so that a normal conversation could be held without being overheard, and without the client being observed								
Rooming in														
Sanitation					Reported availability of improved sanitation: flush/pour flush to piped sewer system or septic tank or pit latrine, pit latrine with slab, composting toilet	Observed availability: The toilet/latrine is classified using uniform criteria for improved sanitation promoted by UNICEF. These include the following: Flush/pour flush to piped sewer system or septic tank or pit latrine, pit latrine (ventilated improved pit (VIP) or other) with slab, composting toilet. There is adequate sanitation facilities accessible (unlocked or key available) for clients on premises	Toilet facilities							

Water supply					Observed availability of an improved water source within 500 meters of facility: piped, public tap, standpipe, tubewell/bore hole, protected dug well, protected spring, rain water	Observed availability: Improved water source uses uniform definitions for safe water sources promoted by UNICEF. These include the following: Piped, public tap, standpipe, tubewell/bore hole, protected dug well, protected spring, rain water. NOTE: The type of base for the standpipe or tubewell is not considered for this question. The water source is located inside the facility or within the ground of the facility	Clean water							
**Inputs: Staffing, training & guidelines**														
Checklist/job aid						Check-lists and/or job-aids for essential childbirth care. Guidelines observed in service area.					checklist/job aid			
Guidelines: BEmONC											guidelines for BEmONC		guidelines for BEmONC	
Guidelines: CEmONC						Country adapt to which guidelines are required/accepted. Guidelines observed in service area.					guidelines for CEmONC			
Guidelines: essential childbirth care						Guidelines for essential childbirth care. Country adapt to which guidelines are required/accepted. Guidelines					Guidelines for essential childbirth observed in service area. Country adapt to which guidelines are required/accepted			

						observed in service area.								
Guidelines: essential newborn care						Guidelines for essential newborn care. Country adapt to which guidelines are required/accepted. Guidelines observed in service area.								
Guidelines: Integrated Management of pregnancy and childbirth (IMPAC)					Observed availability of guidelines for Integrated Management of pregnancy and childbirth (IMPAC)									
Guidelines: management of preterm labour														
Guidelines: standard precaution						Guidelines for standard precautions. Observed availability anywhere in their facility								
Provider knowledge/skills							Clinical health worker 60-item knowledge test and two clinical vignettes							
Supervision											1. staff supervisions 2. staff with observed supervision			
Training: AMTSL														
Training: CEmOC														
Training: surgery														
Training: clean cord care														
Training: early and exclusive breastfeeding														
Training: Integrated Management of pregnancy and childbirth (IMPAC)					At least one staff member providing the service trained in IMPAC in the last 2-3 years									

Training: KMC														
Training: neonatal resuscitation						At least one staff member providing the service trained in newborn resuscitation using bag and mask in the last two years. Interview response from in-charge of service area day of survey.								
Training: newborn infection and management (inlc. Injectable antibiotics)														
Training: routine labour and delivery care						At least one staff member providing the service trained in essential childbirth care in the last two years (other than training on newborn resuscitation using bag and mask). Interview response from in-charge of service area day of survey.					1.qualified staff<br>2.trained			
Training: thermal care														
Skilled birth attendant											skilled person 24 hrs			
**Inputs: Supplies & commodities**														
Amlodipine tablet or alternative calcium channel blocker						Observed in pharmacy or in area where they are routinely stored, at least one with valid expiration date.								
Amoxicillin syrup/suspension or dispersible tablet						Observed in pharmacy or in area where they are routinely stored, at least one with								

						valid expiration date.								
Amoxicillin tablet						Respiratory antibiotic. Observed in pharmacy or in area where they are routinely stored, at least one with valid expiration date.								
Ampicillin powder for injection						Observed in pharmacy or in area where they are routinely stored, at least one with valid expiration date.								
Antibiotic eye ointment					Observed availability of at least one valid unit of antibiotic eye ointment (tetracycline or other) for newborns in service area or where routinely stocked	Antibiotic eye ointment for newborn. Observed in service area OR where routinely stored; in stock with at least one valid.					Antibiotic eye ointment for newborn. Observed in service area OR where routinely stored; in stock with at least one valid.	Eye ointment		
Antibiotics for preterm													Antibiotics for preterm	
Anticonvulsants					Observed availability of at least one valid unit of injectable magnesium sulphate or diazepam in service area or where routinely stocked	Magnesium sulphate 50% injection or alternative strength. Observed in service area OR where routinely stored; in stock with at least one valid.	Magnesium sulfate				Magnesium sulphate 50% injection or alternative strength. Observed in service area OR where routinely stored; in stock with at least one valid			
Storage of infectious waste						Waste receptacle (pedal bin) with lid and plastic bin liner. Observed availability in all three main service areas: general OPD, HIV testing area, and surgery area								

Aspirin cap/tab						Observed in pharmacy or in area where they are routinely stored, at least one with valid expiration date.								
ATC for mother							Apricitabine for mother							
Beclometasone inhaler						Observed in pharmacy or in area where they are routinely stored, at least one with valid expiration date.								
Beta blocker (e.g.bisoprolol, metoprolol, carvedilol, atenolol)						Observed in pharmacy or in area where they are routinely stored, at least one with valid expiration date.								
Blood glucose						Glucometer and glucometer test strips. Able to conduct the test on-site (in the facility) and functioning equipment and reagents needed to conduct the test are observed on-site on the day of the survey. These may be in a laboratory or in the service area where the test is conducted.								
Blood pressure apparatus						Items observed and functioning in the main service area: Digital BP machine or manual sphygmoman	Blood pressure cuff				Digital BP machine or manual sphygmomanometer with stethoscope observed in service area			

						ometer with stethoscope								
Carbamazepine tablet						Observed in pharmacy or in area where they are routinely stored, at least one with valid expiration date.								
Ceftriaxone injection						2nd line injectable antibiotic. Observed in pharmacy or in area where they are routinely stored, at least one with valid expiration date.								
Chlorhexidine												Chlorhexidine for the newborn cord		
Clock/timer/watch							Clock							
Cloth to dry/wrap baby												Clean cloths/towels to dry the baby<br>Cloth to wrap the baby		
Corticosteroids													Betamethasone or dexamethasone. Observed available in pharmacy or where they are routinely stored, at least one with valid expiration date.	
Delivery pack<br>OR all the following individual equipment: cord clamp, episiotomy scissors, scissors or blade to cut cord, suture material with needle, and needle holder					Observed availability of at least one delivery pack OR all the following individual equipment: cord clamp, episiotomy scissors, scissors or blade to cut cord, suture	Delivery pack OR cord clamp, episiotomy scissors, scissors/blade to cut cord, suture material with needle, AND needle holder. Observed availability, reported	Delivery kit				Observed delivery pack OR cord clamp, episiotomy scissors, scissors/blade to cut cord, suture material with needle, AND needle holder in service			

					material with needle, and needle holder	functionality, and in service area or adjacent area.					area or adjacent area			
Delivery pack component: Cord clamps							Cord clamps					Cord ligatures		
Delivery pack component: Sterile scissors or new razor to cut cord												Sterile scissors or new razor blade to cut the cord		
Delivery pack component: Sterile syringes and needles		Health facility with sterile syringes and needles available				Single use — standard disposable or auto-disable syringes. Observed availability anywhere in the facility								
Delivery pack component: Sutures							Sutures							
Diazepam injection						Observed in pharmacy or in area where they are routinely stored, at least one with valid expiration date.								
Enalapril tablet or alternative ACE inhibitor e.g. lisinopril, ramipril, perindopril						Observed in pharmacy or in area where they are routinely stored, at least one with valid expiration date.								
Foetal Stethoscope														
Fluoxetine tablet						Observed in pharmacy or in area where they are routinely stored, at least one with valid expiration date.								
Gauze												Gauze		
Gentamicin injection						Observed in pharmacy or in area where they are								

						routinely stored, at least one with valid expiration date.								
Glibenclamide tablet						Oral treatment type 2 diabetes. Observed in pharmacy or in area where they are routinely stored, at least one with valid expiration date.								
Gloves					Observed availability of latex gloves or equivalent	Latex gloves. If equivalent non latex gloves are available this is acceptable. Observed available in all four main service areas: general OPD, HIV testing area, basic obstetric and newborn care area and surgery area.	Gloves				Sterile latex or equivalent observed in service area	Sterile gloves		
Haemoglobin test kit						This may include colorimeter OR haemoglobin meter OR hemocue. Able to conduct the test on-site (in the facility) and functioning equipment and reagents needed to conduct the test are observed on-site on the day of the survey. These may be in a laboratory or in the service area where the test is conducted.	Haemoglobin test kit							

Haloperidol tablet						Observed in pharmacy or in area where they are routinely stored, at least one with valid expiration date.								
Heat source move to infrastructure?														
HIV diagnostic capacity						RDT kit or ELISA test with ELISA washer, ELISA reader, incubator, specific assay kit. Able to conduct the test on-site (in the facility) and functioning equipment and reagents needed to conduct the test are observed on-site on the day of the survey. These may be in a laboratory or in the service area where the test is conducted.								
Hydralazine							Hydralazine							
Infection control measures in delivery room														
Infection control: Disinfectant							Disinfectant				Disinfectant	Disinfectant		
Infection control: hand rub/disinfectant or delivery room has water and soap						Soap and running water or alcohol based hand rub. Observed in service area.					Soap and running water OR alcohol based hand rub observed in service area			
Infection control: Skin disinfectant					Observed availability of skin disinfectant in service area or where routinely stocked	Skin disinfectant. Observed in service area OR where routinely stored; in stock with at least one valid.					skin disinfectant. Observed in service area OR where routinely stored; in stock with at least one valid			

Infusion set and intravenous fluids					Observed availability of infusion set and intravenous fluids (normal saline or Ringers Lactate or Dextrose 5%)	Normal saline or Ringers Lactate, and Dextrose 5%. Observed in service area OR where routinely stored; in stock with at least one valid.	Intravenous fluids				Normal saline or Ringers Lactate, and Dextrose 5%. Observed in service area OR where routinely stored; in stock with at least one valid.			
Injectable antibiotics					Observed availability of at least one valid unit of broad-spectrum injectable antibiotic (gentamicin, penicillin, or ampicillin or ceftriaxone) in service area or where routinely stocked	Broad-spectrum injectable antibiotic treatment of sepsis in mother and newborn- Specific combination- Ampicillin + gentamicin OR penicillin + gentamicin OR ceftriaxone OR as per country specific formulation. Observed in service area OR where routinely stored; in stock with at least one valid.	Injectable antibiotic for mother				Broad-spectrum injectable antibiotic treatment of sepsis in mother and newborn. Specific combination Ampicillin + gentamicin OR penicillin + gentamicin OR ceftriaxone OR as per country specific formulation. Observed in service area OR where routinely stored; in stock with at least one valid.		Procaine benzylpenicillin (PBP) or gentamicin and ceftriaxone. Observed available in pharmacy or where they are routinely stored, at least one with valid expiration date	
Insulin regular injection						Observed in pharmacy or in area where they are routinely stored, at least one with valid expiration date.								
Malaria diagnostic capacity						RDT kit or smear with microscope, slides, and Wright Giemsa stain. Able to conduct the test on-site (in the facility) and functioning equipment and reagents needed to conduct the test are								

						observed on-site on the day of the survey. These may be in a laboratory or in the service area where the test is conducted.								
Manual vacuum extractor					Observed availability and reported functionality of a manual vacuum extractor	Manual vacuum extractor. Observed availability, reported functionality, and in service area or adjacent area.					Observed manual vacuum extractor in service area or adjacent area			
Measuring cup														
Metformin tablet						Gastroesophageal reflux. Observed in pharmacy or in area where they are routinely stored, at least one with valid expiration date.								
Neonatal antibiotic							Neonatal antibiotic							
Neonatal bag & mask					Observed availability and reported functionality of a newborn bag and mask	Newborn bag and mask (size 1 for term babies AND size 0 for preterm babies). Observed availability, reported functionality, and in service area or adjacent area.	Neonatal ambu-bag and mask				Observed newborn bag and mask (size 1 for term babies AND size 0 for preterm babies) in service area or adjacent area			
Nevirapine for baby							Nevirapine for baby							
Nevirapine for mother							Nevirapine for mother							
Omeprazole tablet or alternative such as pantoprazole, rabeprazole						Observed in pharmacy or in area where they are routinely stored, at least one with valid expiration date.								
Oral rehydration solution						Observed in pharmacy or in area where								

						they are routinely stored, at least one with valid expiration date.								
Partograph	Facility with blank partographs in stock				Observed availability of blank partographs	Blank partograph. Observed in service area.	Partographs				Blank partographs in service area			
Safe final disposal of infectious wastes						Safe final disposal of infectious wastes includes incineration, open burning in protected area, dump without burning in protected area, or remove offsite with protected storage. If method is incineration, incinerator functioning and fuel available. Observed final disposal/holding site for infectious wastes and verify no unprotected waste is observed.								
Safe final disposal of sharps						Safe final disposal of sharps includes incineration, open burning in protected area, dump without burning in protected area, or remove offsite with protected storage. If method is incineration, incinerator functioning and fuel available. Observed final disposal/holdi								

						ng site for sharps and verify no unprotected sharps are observed.								
Salbutamol inhaler						Chronic asthma attacks. Observed in pharmacy or in area where they are routinely stored, at least one with valid expiration date.								
Scale						Items observed and functioning in the main service area: Adult scale & child scale with weight gradation minimum 250 grams. A digital standing scale where adult holds child and gradations go to 250 grams is acceptable	Infant and/or child scale				Infant weighing scale observed in service area			
Sharps box/Appropriate storage of sharps waste						A puncture-resistant, rigid, leak resistant container designed to hold used sharps safely during collection, disposal and destruction. Sharps containers should be made of plastic, metal, or cardboard and have a lid that can be closed. Sharps containers should be fitted with a sharps aperture, capable of receiving								

						syringes and needle assemblies of all standard sizes, together with other sharps. Boxes must be clearly marked with the international biohazard warning not less than 50mm diameter, printed in black or red on each of the front and back faces of the box. Observed availability in all three main service areas: general OPD, HIV testing area, and surgery area.								
Simvastatin tablet or other statin e.g. atorvastatin, pravastatin, fluvastatin						High cholesterol. Observed in pharmacy or in area where they are routinely stored, at least one with valid expiration date.								
Stainless steel bowl							Stainless steel bowls							
Sterilisation equipment					Observed availability and reported functionality of either a dry heat sterilizer or an autoclave	This is usually either a dry heat sterilizer or an autoclave. If the machine is not electric, then make sure that the heat source is available and (If relevant) functioning (e.g., wood or gas is present for the autoclave). Observed availability anywhere in the facility	Sterilization equipment							

						reported functionality.								
Stethoscope						Items observed and functioning in the main service area: stethoscope	Stethoscope							
Suction apparatus					Observed availability and reported functionality of suction bulb or electric suction pump or suction catheter	Suction bulb (single use or sterilisable multi-use) or electric suction pump AND suction catheter for suctioning newborn. Observed availability, reported functionality, and in service area or adjacent area.	Mucus suction				Observed to have suction bulb (single use or sterilisable multi-use) or electric suction pump AND suction catheter for suctioning newborn in service area or adjacent area			
Surface disinfectant						Chlorine-based or other country specific used for environmental disinfection								
Syphilis rapid test						RDT kit. Able to conduct the test on-site (in the facility) and functioning equipment and reagents needed to conduct the test are observed on-site on the day of the survey. These may be in a laboratory or in the service area where the test is conducted.								
Thermometer						Items observed and functioning in the main service area: thermometer	Thermometer							
Thiazide (e.g. hydrochlorothiazide)						Observed in pharmacy or in area where they are routinely								

						stored, at least one with valid expiration date.								
Urine glucose dipstick						Dipsticks for urine glucose (with valid expiration date). Able to conduct the test on-site (in the facility) and functioning equipment and reagents needed to conduct the test are observed on-site on the day of the survey. These may be in a laboratory or in the service area where the test is conducted.								
Urine protein dipstick						Dipsticks for urine protein (with valid expiration date). Able to conduct the test on-site (in the facility) and functioning equipment and reagents needed to conduct the test are observed on-site on the day of the survey. These may be in a laboratory or in the service area where the test is conducted.								
Urine test for pregnancy						RDT kit. Able to conduct the test on-site (in the facility) and functioning equipment and reagents needed to conduct the test are observed on-								

						site on the day of the survey. These may be in a laboratory or in the service area where the test is conducted.								
Uterotonic		Facility with oxytocin or ergometrine available			Observed availability of at least one valid unit of injectable uterotonic (oxytocin or other) in service area or where routinely stocked	Oxytocin. Observed in service area OR where routinely stored; in stock with at least one valid.	Uterotonic				Oxytocin. Observed in service area OR where routinely stored; in stock with at least one valid.	Oxytocin, Ergometrine, Misoprostol, Syntometrine		
Vacuum aspirator or D&C kit					Observed availability and reported functionality of a vacuum aspirator or D&C kit	Vacuum aspirator or D&C kit (with speculum). Observed availability, reported functionality, and in service area or adjacent area.					Observed vacuum aspirator or D&C kit (with speculum) in service area or adjacent area			
Zinc sulphate tablets, dispersible tablets or syrup						Observed in pharmacy or in area where they are routinely stored, at least one with valid expiration date.								
**Inputs: service availability**														
CEmOC: blood transfusion				Reported performance of blood transfusion in the three months before the health facility survey										
CEmOC: c-section				Reported performance of caesarean section in the three months before the health facility survey										
EmOC: Administers antibiotic				Reported performance of parenteral antibiotics in the three months		Parenteral administration of antibiotics for mothers	Parenteral antibiotics provided in last 3 months				Facility offering parenteral administration of antibiotics for mothers			

				before the health facility survey										
EmOC: Assisted/instrumental vaginal delivery				Reported performance of assisted vaginal delivery in the three months before the health facility survey		Assisted vaginal delivery					Facility offering assisted vaginal delivery			
EmOC: Corticosteroids						Facility offers: Corticosteroids in preterm labour								
EmOC: Manual removal of placenta				Reported performance of manual removal of placenta in the three months before the health facility survey		Facility offers: Manual removal of placenta	Manual removal of placenta provided in last 3 months				Facility offering manual removal of placenta			
EmOC: Manual removal of retained products				Reported performance of manual removal of retained products in the three months before the health facility survey		Facility offers: Manual removal of retained products	Removal of retained products of conception provided in the last 3 months				Facility offering manual removal of retained products			
EmOC: Parenteral administration of anticonvulsants for hypertensive disorders of pregnancy				Reported performance of parenteral anticonvulsants in the three months before the health facility survey		Parenteral administration of anticonvulsants	Parenteral anticonvulsants provided in the last 3 months							
EmOC: Parenteral uterotonic for haemorrhage				Reported performance of parenteral uterotonics in the three months before the health facility survey		Facility offers: Routine administration of oxytocin injection immediately after birth to all women for the prevention of post-partum haemorrhage	Uterotonic provided in the last 3 months				Facility offers routine administration of oxytocin injection immediately after birth to all women for the prevention of postpartum haemorrhage			
Newborn signal function: baby weigh														
Newborn signal function: Breastfeeding						Facility offers: Immediate and exclusive breastfeeding							Facility offering immediate and routine breastfeeding	

Newborn signal function: cord care						Facility offers hygienic cord care: Cut with sterile item and apply disinfectant to tip and stump, and no application of other substances							Service availability: facility offering hygienic cord care. Cut with sterile item and apply disinfectant to tip and stump, and no application of other substances	
Newborn signal function: Drying and wrapping						Facility offers: Thermal protection (drying baby immediately after birth and wrapping)							Facility offers Thermal protection (drying baby immediately after birth and wrapping)	
Newborn signal function: KMC for LBW babies						Facility offers: KMC (Kangaroo mother care) for premature/very small babies							Facility offering KMC for LBW babies	
Newborn signal function: Neonatal resuscitation				Reported performance of neonatal resuscitation in the three months before the health facility survey		Facility offers: Neonatal resuscitation with bag and mask	Newborn resuscitation with bag and mask provided in the last 3 months						Facility offering neonatal resuscitation with bag and mask	
Newborn signal function: PROM						Facility offers: Antibiotics for preterm or prolonged PROM to prevent infection								
Newborn signal function: sepsis management						Facility offers: Injectable antibiotics for neonatal sepsis								
Newborn signal function: skin-to-skin														
Newborn: BCG vaccine														
Newborn: polio vaccine														
Newborn: postnatal check														
Partograph						Facility offers: Monitoring and management of labour					Facility offers monitoring and management of labour			

						using partograph					using partograph			
Vitamin A														
**Intervention coverage**														
Administer prophylaxis for eyes/apply eye ointment														HCW report at last delivery: administer prophylaxis for the eyes
AMSTL: Active management of 3rd stage labour										SBA reported undertaking active management of third stage of labour at last birth attended				
AMSTL: Administers uterotonic/Parenteral uterotonic		HCW reported giving an oxytocic agent during the last delivery attended								SBA reported undertaking administration of prophylactic uterotonics to prevent post-partum haemorrhage during last birth attended				
AMSTL: Controlled cord traction														
AMSTL: Prepares uterotonic														
AMSTL: Uterine massage														
Apgar score							Average of all deliveries recorded in the facility delivery Apgar score							
Baby weighed							Average of all deliveries recorded in the facility delivery baby weighed							HCW report at last delivery: weigh the baby
Breastfeeding			Observation of breastfeeding initiation within 1 hour of delivery				Average of all deliveries recorded in the facility delivery baby							HCW report at last delivery: initiate breastfeeding

							breastfed within 1h							within the first hour
Check breathing														HCW report at last delivery: ensure the baby is breathing
Check cord care														
Clean baby's mouth before shoulder comes out														HCW report at last delivery cleans baby's mouth before shoulder comes out
Clean baby's mouth, face and nose														HCW report at last delivery: clean baby's mouth, face and nose
Cord care														HCW report at last delivery: care for the umbilical cord
Cord care: alcohol														HCW report at last delivery: cord care alcohol
Cord care: chlorhexidine														HCW report at last delivery: apply chlorhexidine
Cord care: cord wrapped in dry cloth														HCW report at last delivery: cord wrapped in dry cloth
Cord care: tie or clamp cord after 2/3 mins														
Disposable delivery kit used														
Breastfeeding														
EmNC: Dexamethasone to mother for premature labour														
EmNC: Injectable antibiotics for newborn sepsis														
EmNC: Intravenous fluids for newborns														
EmNC: Kangaroo mother care														

EmNC: Neonatal resuscitation														HCW report at last delivery: resuscitation open the airways, clean the mouth/use suction device, stimulating/drying/wrapping the baby, use the ambu bag, heart massage
EmOC: Assisted/instrumental vaginal delivery														
EmOC: Blood transfusions														
EmOC: Caesarean sections														
EmOC: Manual removal of placenta														
EmOC: Manual removal of retained products														
EmOC: Parenteral antibiotics or antibiotics for maternal infection														
EmOC: Parenteral anticonvulsants														
EmOC: Parenteral oxytocin for haemorrhage														
Examine perineal and vaginal lacerations														
Examine placenta/assesses completeness of placenta and membranes														
HIV test							Average of all deliveries recorded in the facility delivery HIV test							
Infection prevention/wash hands before examination														
Iron folate tablets														
Palpitates uterus 15 min after delivery														
Thermal care: Baby dried/wrapped														HCW report at last delivery: Ensure the baby is dry

Thermal care: Baby kept dry/warm														HCW report at last delivery: ensure baby is kept warm
Thermal care: Bathing delayed														
Thermal care: Skin-to-skin			Observation of newborn placed skin-to-skin											
Wear sterile gloves for vaginal examination														
**Process quality-adjusted coverage**														
**Quality: process**														
Asks about headaches, bleeding														
Delivered on a clean floor/bed														
Maternal blood pressure							Average of all deliveries recorded in the facility delivery maternal blood pressure					HCW report at last delivery: monitor maternal blood pressure		
Maternal pulse												HCW report at last delivery: monitor maternal pulse		
Maternal temperature												HCW report at last delivery: monitor maternal temperature		
Monitor colour of amniotic fluid												HCW report at last delivery: Monitor colour of amniotic fluid		
Monitor degree of molding												HCW report at last delivery: Monitor degree of molding		
Monitor descent of head												HCW report at last delivery: Monitor descent of the head		
Monitor dilation of cervix												HCW report at last delivery: Monitor dilation of the cervix		
Monitor foetal heartbeat												HCW report at last		

												delivery: Monitor foetal heartbeat		
Monitor labour progress												HCW report at last delivery: Monitor labour progress		
Monitor uterine contractions												HCW report at last delivery: monitor uterine contraction		
Observe baby colour														HCW report at last delivery: Observe for colour
Partograph	HCW reported using a partograph during the last delivery attended						Average of all deliveries recorded in the facility delivery partographs							
Postnatal check/Initial assessment child's health														HCW report at last delivery: evaluate/examine the newborn within the first hour
Postpartum check														
Rooming in			Observation keeping the mother and newborn in the same room											
**Quality: interpersonal**														
Explain what will happen in labour														

Support person at birth															
**User-adherence coverage**															
**Outcomes-adjusted coverage**															
Death								Proportion of deliveries without complications or death	Proportion of live births reaching 28 days without death due to respiratory infection, noso comial infection or sepsis						

TABLE NOTE: * extraction based on SARA general service readiness indicators & specific availability & readiness indicators for basic obstetric and newborn care. Authors state used all 70 binary general and delivery-related SARA indicators; we identified 86 in extraction and not possible to determine which were used by the authors.

Table S4 continued….

Study (data sources)	Nesbitt et al. 2013 (54) (Surveillance data; HF assessment)	Nguhiu et al. 2017 (40) (DHS; SPA)	Nguyen et al. 2021 (44) (DHS, SPA)	Okawa et al. 2019a (49) (HH survey)	Okawa et al. 2019b (46) (HH survey)	Sharma et al. 2017 (41) (SPA, DHS, the Oxford Poverty and Human Development Initiative, observations)		Shibanuma et al. 2018 (58) (HH survey)	Wang et al. 2019 (36) (DHS; SPA)	Willey et al. 2018 (56) (HH survey; HF assessment)
**Health service/intervention**	**Intrapartum & immediate newborn care**	**Skilled delivery & perinatal care**	**Birth care**	**Peripartum care**	**Peripartum care**	**(1) Delivery care: inputs**	**(2) Delivery care: processes of care**	**Facility delivery**	**Facility delivery**	**Basic emergency obstetric care**
**Target population**										
Women who have given birth	Live births in study area	Women 15–49 years old with at least one child under 5 years	Women 15–49 years old with a live birth in the 3 years preceding the survey	Women between 6 wks -12 mnths postpartum	Women aged 15-49 yrs delivered in 2 yrs prior to survey	Total population at county level	Total population at county level	Women aged 15-49 yrs delivered live or stillbirth in 2 yrs prior to survey	Number of live births in 2yrs prior to survey	Women aged 15-49 years who reported a live birth in the 2yrs prior to survey
**Service contact coverage**										
Facility based childbirth	Facility delivery	Most recent birth, reported attendance by a skilled health provider (doctor, nurse or midwife)	Most recent birth, reported delivery in a health facility	Delivery at a healthcare facility with the assistance of skilled care providers	Delivery with assistance of skilled healthcare providers at a healthcare facility	Population living with 5km of facility	Population living with 5km of facility	Delivered at a health facility and assisted by a skilled birth attendant	Facility-based live births in 2 yrs preceding survey	Women reported place of birth & attended by SBA
**Input-adjusted coverage**										
**Inputs: service infrastructure**										
Ambulance/Emergency transport						Ambulance			The facility had a functioning ambulance or other vehicle for emergency transport that was stationed at the facility and had fuel available on the day of the assessment, or the facility has access to an ambulance or other vehicle for emergency transport that is stationed at another facility or that operates from another facility.	
Communication equipment						Communication				
Computer with email/internet access										
Delivery beds						Delivery beds			At least one delivery bed available and observed in delivery area.	
Examination light						Exam light			Examination light (flashlight okay) available, observed, and functioning in delivery area.	
Facility register		HCW report and observed register for delivery clients present								

Patient toilet clean & water & soap for handwashing	Observed patient toilet is clean & has water and soap for handwashing									
Power/Electricity						Electricity			Facility is connected to a central power grid and there has not been an interruption in power supply lasting for more than two hours at a time during normal working hours in the seven days before the assessment, or the facility had a functioning generator with	Source of electricity 24 hr/day available on the day of the survey
Private delivery room						Private delivery room				
Rooming in		HCW report rooming in mother/newborn								
Sanitation	Patient toilet exists								Facility has a functioning flush or pour-flush toilet, a ventilated improved pit latrine, or composting toilet.	
Water supply	Reported clean water source					Water			Facility has an improved water source available. For most countries, this means that water is piped into the facility or onto facility grounds, or else water comes from a public tap or standpipe, a tube well or borehole, a protected dug well, protected spring, rain water, or bottled water, and the outlet from this source is within 500 meters of the facility	Source of running water 24/hr available on the day of the survey
**Inputs: Staffing, training & guidelines**										
Checklist/job aid										
Guidelines: BEmONC			Guidelines on basic birth care (BEmONC)							
Guidelines: CEmONC			Guidelines on comprehensive birth care (CEmONC)						CEmOC guidelines available in delivery area	
Guidelines: essential childbirth care										

Guidelines: essential newborn care										
Guidelines: Integrated Management of pregnancy and childbirth (IMPAC)									Integrated Management of Pregnancy and Childbirth (IMPAC) guidelines available in delivery area	
Guidelines: management of preterm labour									Guidelines for management of preterm labour available in delivery area	
Guidelines: standard precaution									Guidelines for standard precautions available in delivery area	
Provider knowledge/skills										
Supervision									At least half of interviewed providers reported being personally supervised at least once during the 6 months preceding the survey	
Training: AMTSL									At least one provider of delivery/newborn care in facility received training in AMTSL in the past 24 months	
Training: CEmOC									At least one provider of delivery/newborn care in facility received training in IMPAC (presented as reported potentially author meant CEmOC) in the past 24 months	
Training: surgery	≥ 1 doctor conducting caesarean section									
Training: clean cord care									At least one provider of delivery/newborn care in facility received training in cord care in the past 24 months	
Training: early and exclusive breastfeeding									At least one provider of delivery/newborn care in facility received training in early and exclusive breastfeeding in the past 24 months	
Training: Integrated Management of			Staff with any training on IMPACT						At least one provider of delivery/newborn	

pregnancy and childbirth (IMPAC)									care in facility received training in IMPAC in the past 24 months	
Training: KMC									At least one provider of delivery/newborn care in facility received training in KMC in the past 24 months	
Training: neonatal resuscitation	≥ 1 health professional trained in neonatal resuscitation								At least one provider of delivery/newborn care in facility received training in neonatal resuscitation in the past 24 months	
Training: newborn infection and management (inlc. Injectable antibiotics)									At least one provider of delivery/newborn care in facility received training in newborn infection management (including injectable antibiotics) in the past 24 months	
Training: routine labour and delivery care						HCW trained in delivery care			At least one provider of delivery/newborn care in facility received training in routine care during labour and normal vaginal delivery in the past 24 months	
Training: thermal care									At least one provider of delivery/newborn care in facility received training in thermal care in the past 24 months	
Skilled birth attendant	Human resource capacity for 24 hour service availability: ≥3 skilled health professionals employed					24-hour delivery care			Provider of delivery care available on-site or on-call 24 hours/day, with observed duty schedule	
**Inputs: Supplies & commodities**										
Amlodipine tablet or alternative calcium channel blocker										
Amoxicillin syrup/suspension or dispersible tablet										
Amoxicillin tablet										
Ampicillin powder for injection										
Antibiotic eye ointment									Tetracycline eye ointment for	

									newborn available in delivery area and at least one dose valid.	
Antibiotics for preterm										
Anticonvulsants	Observed Diazepam or Magnesium Sulfate					Magnesium sulfate			Magnesium sulphate available in delivery area with at least one dose valid.	Essential drugs for management of complications in mothers and babies available: parenteral anticonvulsants
Storage of infectious waste										
Aspirin cap/tab										
ATC for mother										
Beclometasone inhaler										
Beta blocker (e.g.bisoprolol, metoprolol, carvedilol, atenolol)										
Blood glucose										
Blood pressure apparatus	Reported sphygmomanometer available		Manual or digital BP apparatus			Blood pressure cuff			Manual or digital blood pressure apparatus observed and functioning in delivery area.	Commodities to monitor and manage labour available on day of survey: blood pressure cuff
Carbamazepine tablet										
Ceftriaxone injection										
Chlorhexidine									Chlorhexidine solution (4%) for umbilical cord cleaning available in delivery area, with at least one dose valid.	
Clock/timer/watch	Observed clock									Commodities to monitor and manage labour available on day of survey: timer
Cloth to dry/wrap baby						Towels				
Corticosteroids	Observed Dexamethasone								Hydrocortisone observed at the facility and at least one dose valid.	
Delivery pack OR all the following individual equipment: cord clamp, episiotomy scissors, scissors or blade to cut cord, suture material with needle, and needle holder									Delivery pack OR cord clamp, episiotomy scissors, scissors/blade to cut cord, suture material with need, AND needle holder all available in delivery area.	
Delivery pack component: Cord clamps						Umbilical cord clamps				Commodities for hygienic core care available on day of survey: cord tie

Delivery pack component: Sterile scissors or new razor to cut cord						blade				Commodities for hygienic core care available on day of survey: Sterile cord cutter
Delivery pack component: Sterile syringes and needles	Observed small syringes/needs for babies									
Delivery pack component: Sutures										
Diazepam injection										
Enalapril tablet or alternative ACE inhibitor e.g. lisinopril, ramipril, perindopril										
Foetal Stethoscope	Observed fetoscope									Commodities to monitor and manage labour available on day of survey: foetal stethoscope
Fluoxetine tablet										
Gauze										
Gentamicin injection										
Glibenclamide tablet										
Gloves									Disposable latex gloves observed in delivery area.	Commodities for infection prevention available on day of survey: disposable gloves
Haemoglobin test kit										
Haloperidol tablet										
Heat source move to infrastructure?						Heat source				
HIV diagnostic capacity										
Hydralazine										
Infection control measures in delivery room						Infection control measures in delivery room				
Infection control: Disinfectant										Commodities for infection prevention available on day of survey: disinfectant
Infection control: hand rub/disinfectant or delivery room has water and soap	Observed sink with soap for hand washing								Hand-washing soap and running water or hand disinfectant available and observed in delivery area.	Commodities for infection prevention available on day of survey: soap

Infection control: Skin disinfectant									Skin disinfectant available for newborns in delivery area.	
Infusion set and intravenous fluids	Reported availability of intravenous fluids with infusion sets								IV solution with infusion set available in delivery area with at least one set valid.	
Injectable antibiotics	Reported availability of ampicillin or gentamicin								Injectable antibiotics observed in delivery area (i.e., at “service site”) and at least one dose valid.	Essential drugs for management of complications in mothers and babies available: parenteral antibiotics for maternal infection and newborn sepsis
Insulin regular injection										
Malaria diagnostic capacity										
Manual vacuum extractor									Manual vacuum extractor available, observed, and functioning in the delivery area.	
Measuring cup	Observed graduated measuring cup									
Metformin tablet										
Neonatal antibiotic										
Neonatal bag & mask	Observed bag + mask for baby					Newborn bag & mask			Newborn bag and mask (AMBU bag and mask) available, observed, and functioning in the delivery area.	Bag & mask available on day of survey
Nevirapine for baby										
Nevirapine for mother										
Omeprazole tablet or alternative such as pantoprazole, rabeprazole										
Oral rehydration solution										
Partograph	Observed correctly filled partograph					Partographs			Partograph available, observed, and functioning in delivery area.	
Safe final disposal of infectious wastes										
Safe final disposal of sharps										
Salbutamol inhaler										
Scale	Reported weighing scale		Infant scale						Infant scale observed and functioning in delivery area.	

Sharps box/Appropriate storage of sharps waste										Commodities for infection prevention available on day of survey: sharps box
Simvastatin tablet or other statin e.g. atorvastatin, pravastatin, fluvastatin										
Stainless steel bowl										
Sterilisation equipment									Facility reports that some instruments are processed in the facility and the facility has a functioning electric dry heat sterilizer, a functioning electric autoclave, or a non-electric autoclave with a functioning heat source available somewhere in the facility.	Commodities for infection prevention available on day of survey: sterilizer
Stethoscope						Stethoscope				
Suction apparatus									Suction apparatus (mucus abstractor) available, observed, and functioning in the delivery area.	
Surface disinfectant										
Syphilis rapid test										
Thermometer										Commodities to monitor and manage labour available on day of survey: thermometer
Thiazide (e.g. hydrochlorothiazide)										
Urine glucose dipstick										
Urine protein dipstick										Commodities to monitor and manage labour available on day of survey: urine protein dipstick
Urine test for pregnancy										

Uterotonic	Observed oxytocin					Injectable oxytocic			Oxytocin observed in delivery area with at least one dose valid.	Essential drugs for management of complications in mothers and babies available on day of survey: parenteral oxytocics for haemorrhage and uterotonics for active management of the third stage of labour
Vacuum aspirator or D&C kit									Vacuum aspirator or D&C kit available, observed, and functioning, in the delivery area.	
Zinc sulphate tablets, dispersible tablets or syrup										
**Inputs: service availability**										
CEmOC: blood transfusion									Facility performed blood transfusion at least once during the three months before the assessment (incorporate the availability of equipment and materials for performing the service)	
CEmOC: c-section									Facility performed caesarean section at least once during the three months before the assessment (incorporate the availability of equipment and materials for performing the service)	
EmOC: Administers antibiotic									Facility performed parenteral administration of antibiotics at least once during the three months before the assessment	
EmOC: Assisted/instrumental vaginal delivery									Facility performed assisted vaginal delivery at least once during the three months before the assessment	
EmOC: Corticosteroids										
EmOC: Manual removal of placenta									Facility performed manual removal of placenta at least once during the three months before the assessment	

EmOC: Manual removal of retained products									Facility performed removal of retained products at least once during the three months before the assessment	
EmOC: Parenteral administration of anticonvulsants for hypertensive disorders of pregnancy									Facility performed parenteral administration of anticonvulsants for hypertensive disorders of pregnancy at least once during the three months before the assessment	
EmOC: Parenteral uterotonic for haemorrhage									Facility performed parenteral administration of uterotonic drugs/oxytocin at least once during the three months before the assessment	
Newborn signal function: baby weigh		HCW report facility routinely weigh the newborn immediately								
Newborn signal function: Breastfeeding									Facility reported breast feeding in 1st hour is routinely practiced	
Newborn signal function: cord care										
Newborn signal function: Drying and wrapping		HCW report facility routinely dries and wraps newborn to keep them warm							Facility reported drying and wrapping newborns is routinely practiced	
Newborn signal function: KMC for LBW babies		HCW report facility practice kangaroo mother care								
Newborn signal function: Neonatal resuscitation									Facility performed neonatal resuscitation at least once during the three months before the assessment	
Newborn signal function: PROM										
Newborn signal function: sepsis management										
Newborn signal function: skin-to-skin									Facility reported skin-to-skin is routinely practiced	
Newborn: BCG vaccine		HCW report facility routinely gives newborn BCG prior to discharge								

Newborn: polio vaccine		HCW report facility routinely give newborn oral polio vaccine prior to discharge								
Newborn: postnatal check		HCW reports facility routinely completes exam of newborn performed before discharge								
Partograph										
Vitamin A		HCW report facility routinely vitamin A given to mother								
**Intervention coverage**										
Administer prophylaxis for eyes/apply eye ointment	Report always apply eye ointment to the baby's eyes after delivery									
AMSTL: Active management of 3rd stage labour										
AMSTL: Administers uterotonic/Parenteral uterotonic	Reports always administering injection of oxytocin within 1 minute of delivery						Administer uterotonic correctly			
AMSTL: Controlled cord traction	Reports always performing controlled cord traction									
AMSTL: Prepares uterotonic							Prepare uterotonic drug			
AMSTL: Uterine massage	Reports always undertaking uterine massage									
Apgar score										
Baby weighed	Reports always weigh baby after delivery			Women reported birth weight measured						
Breastfeeding	Reports always initiating breastfeeding within 1 hr of delivery			Women reported breast feeding initiated <30 min	Women reported breast feeding initiated <30 min		Mother initiates breastfeeding within 1 hour	Women reported breast feeding initiated within 1 hr of delivery		
Check breathing										
Check cord care										
Clean baby's mouth before shoulder comes out										

Clean baby's mouth, face and nose										
Cord care										
Cord care: alcohol										
Cord care: chlorhexidine										
Cord care: cord wrapped in dry cloth										
Cord care: tie or clamp cord after 2/3 mins							Tie or clamp cord after 2/3 minutes			
Disposable delivery kit used				Women reported disposable delivery kit used						
Breastfeeding	Report teaching mother to express milk and feed with spoon and cup if baby unable to breastfeed									
EmNC: Dexamethasone to mother for premature labour	Report performing dexamethasone to mother for premature labour									
EmNC: Injectable antibiotics for newborn sepsis	Report performing injectable antibiotics for newborn sepsis									
EmNC: Intravenous fluids for newborns	Report performing intravenous fluids for newborns									
EmNC: Kangaroo mother care	Report teaching mother skin-to-skin or KMC for LBW babies									
EmNC: Neonatal resuscitation	Report performing newborn resuscitation with bag & mask						Prepare newborn bag & mask			
EmOC: Assisted/instrumental vaginal delivery	Report performing instrumental delivery									
EmOC: Blood transfusions	Report performing blood transfusion									
EmOC: Caesarean sections	Report performing caesarean section									
EmOC: Manual removal of placenta	Report performing manual removal of placenta									
EmOC: Manual removal of retained products	Report performing manual removal of retained products of conception									
EmOC: Parenteral antibiotics or antibiotics for maternal infection	Report performing parenteral antibiotic									

EmOC: Parenteral anticonvulsants	Report performing: parenteral anticonvulsants									
EmOC: Parenteral oxytocin for haemorrhage	Report performing parenteral oxytocin									
Examine perineal and vaginal lacerations							Examine for perineal & vaginal lacerations			
Examine placenta/assesses completeness of placenta and membranes							Examine placenta			
HIV test										
Infection prevention/wash hands before examination	Reports always using measures of infection prevention during delivery						Wash hands before any examination			
Iron folate tablets										
Palpitates uterus 15 min after delivery							Palpate uterus 15 min after delivery			
Thermal care: Baby dried/wrapped	Reports always dry baby immediately after delivery			Women reported newborn body dried	Women reported newborn body dried		Dry newborn immediately			
Thermal care: Baby kept dry/warm										
Thermal care: Bathing delayed	Reports always delaying bathing for at least 6 hours after delivery			Women reported first bathing of newborn after 6 hours						
Thermal care: Skin-to-skin	Reports always place baby on mother's abdomen after delivery			Women reported skin-to-skin contact	Women reported skin-to-skin contact		Place newborn skin-to-skin if baby breathing	Women reported skin-to-skin contact		
Wear sterile gloves for vaginal examination							Wear sterile gloves for vaginal examination			
**Process quality-adjusted coverage**										
**Quality: process**										
Asks about headaches, bleeding							Asks re headaches. Bleeding			
Delivered on a clean floor/bed				Women reported delivered on a clean floor/bed						
Maternal blood pressure	Reports always measuring blood pressure						Take blood pressure			
Maternal pulse							Take mother's pulse			
Maternal temperature										
Monitor colour of amniotic fluid										
Monitor degree of molding										
Monitor descent of head										
Monitor dilation of cervix										

Monitor foetal heartbeat										
Monitor labour progress										
Monitor uterine contractions										
Observe baby colour										
Partograph	Facility reports always monitoring labour with partograph						Initiate use of partograph			
Postnatal check/Initial assessment child's health										
Postpartum check							Take mother's vital signs 15 min after delivery			
Rooming in										
**Quality: interpersonal**										
Explain what will happen in labour							Explain what will happen in labour			
Support person at birth	Report that woman can choose to have delivery companion									
**User-adherence coverage**										
**Outcomes-adjusted coverage**										
Death										

Table S5 Care of sick newborns

Study (data sources)	Murphy et al. 2018 (38) (HF assessment; medical records)
**Health service**	**Inpatient neonatal care**
**Target population**	
	Number of newborns requiring care; estimated by applying the rate of live births requiring inpatient services (183 per 1000 live births) to the total number of live births in the study region between mid-2014 to mid-2015
**Service contact**	
	Total number of neonatal admissions to INC facilities
**Input-adjusted coverage**	
**Inputs: service infrastructure**	
Power/Electricity	Consistent power (outages <monthly) or generator serving NBU
Heat source	Heating in NBU
Water supply	Running water
**Inputs: Supplies & commodities**	
Laboratory services	1. Minimum package of care:<br>i. Open 24/7<br>ii. Test for haemoglobin<br>iii. Test for bilirubin (blood test)<br>iv. Glucose tests<br>v. Blood grouping and cross match<br>vi. Electrolytes (sodium / potassium)<br>2. Blood bank<br>3. Blood slide microscopy for malaria parasites<br>4. Test for direct Coombs test<br>5. Urea or creatinine<br>6. Liver function tests (enzymes e.g. AST/ALT)<br>7. Microscopy & culture: Pus swab and urine culture<br>8. CSF microscopy<br>9. Coagulation profile<br>10. Blood culture ability
Hygiene	1. Cleaning/disinfectant supplies<br>2. Sharps disposed in a special container<br>3. Clean gloves available<br>4. Separate clinical and non-clinical waste<br>5. Sinks with soap and water for hand-washing<br>6. Mother has access to running water<br>7. Alcohol hand rub

Safe delivery equipment and drugs for mother	Equipment available on the ward and working on the day of the visit<br>1. Thermometer<br>2. Sterile syringes<br>3. Sterile needles<br>4. Sterile vaginal examination packs<br>5. Sterile delivery set (complete)<br>6. A stethoscope<br>7. Amnicots/sterile Kocker's forceps for artificial rupture of membrane<br>8. Urine dipstick kits/strips<br>9. Urinary catheters<br>10. Vacuum (such as Kiwi) for assisted vaginal delivery<br>11. Manual vacuum aspirator (MVA)<br>12. Long gloves for manual removal of placenta<br>13. Guedel airways – these should be a full range of sizes<br>14. Bag Valve Mask (BVM) device: adult size bag and mask<br>15. Oxygen source (any and working)<br>16. Nasal catheters/prongs<br>17. Oxygen face –masks (with and without reservoir bags)<br>18. Oxygen flow regulators<br>19. Laryngoscope<br>20. Laryngoscope blades (straight, curved, and different sizes)<br>21. Endotracheal tubes (of different sizes)<br>22. IV fluid giving sets<br>23. Blood giving set<br>24. Adult IV cannula<br>25. Blood pressure monitor (any and working)<br>Drugs available on the ward or accessible within five minutes without administrative barriers<br>1. Adrenaline<br>2. Magnesium sulphate<br>3. Lasix<br>4. Digoxin<br>5. Morphine<br>6. Oxytocin<br>7. Dexamethasone<br>8. Prostaglandin F2 alpha<br>9. Calcium gluconate<br>10. Penicillin<br>11. Gentamicin<br>12. Ceftriaxone/Cefuroxime
Neonatal resuscitation equipment	Available on the ward and working on the day of the visit<br>1. Thermometer<br>2. Weighing scales<br>3. Sterile syringes<br>4. Sterile needles<br>5. Warm dry towels for dying and wrapping the newborn<br>6. Sterile cord clamp<br>7. Sterile scissors<br>8. A firm stable surface for placing the newborn for resuscitation (where warmth can be maintained)<br>9. An overhead light source above the surface for resuscitation<br>10. A clock in view or reach of surface for resuscitation<br>11. A stethoscope<br>12. Suction tubes/catheters<br>13. Suction Machine<br>14. Guedel airways – these should be a full range of sizes<br>15. Bag Valve Mask (BVM) devices: bag size 500 ml or 750 ml, that are in working order with newborn face masks (sizes 0 and 1)<br>16. Oxygen source (any and working)<br>17. Nasal catheters/prongs<br>18. Oxygen face –masks (with and without reservoir bags)<br>19. Oxygen flow regulators<br>20. Warming equipment-working radiant heaters

Essential ward equipment in the NBU for treatment and diagnostic procedures	Available on the ward and working on the day of the visit<br>1. Thermometer<br>2. Weighing scales<br>3. Sterile syringes<br>4. Sterile needles<br>5. A stethoscope<br>6. Suction tubes/catheters<br>7. Suction Machine<br>8. Guedel airways – these should be a full range of sizes<br>9. Bag Valve Mask (BVM) devices: bag size 500 ml or 750 ml, that are in working order with newborn face masks (sizes 0 and 1)<br>10. Oxygen source (any and working)<br>11. Nasal catheters/prongs<br>12. Oxygen face –masks (with and without reservoir bags)<br>13. Oxygen flow regulators<br>14. Warming equipment-working radiant heaters<br>15. Kangaroo mother care wraps<br>16. Phototherapy equipment<br>17. Eye protection for phototherapy<br>18. Blood transfusion giving set
IV fluid and feeds in the NBU	1. Feeding cups for giving expressed breast milk<br>2. IV fluid burette<br>3. Infusion set / adult IV fluid set<br>4. Paediatric cannula<br>5. Nasogastric tube (FG6 or 8 or other)<br>6. glucose 10%<br>7. normal saline IV or ringers lactate<br>8. term formula
NBU drugs	Available if they were on the ward or accessible within five minutes without administrative barriers. * considered available if on the ward or available within the facility and within 2 hours of request<br>1. Vitamin K<br>2. Nevirapine solution<br>3. Prophylactic tetracycline eye ointment*<br>4. Phenobarbitone (injection)<br>5. Phenytoin (injection) *<br>6. Aminophylline*<br>7. Penicillin (injection)<br>8. Gentamicin or Amikacin<br>9. Ampicillin / Cloxacillin (injection) *<br>10. Oral Cloxacillin *<br>11. Oral erythromycin*<br>12. Metronidazole (injection) *<br>13. Ceftriaxone or cefotaxime*<br>14. Ferrous Fumarate suspension*<br>15. Folate drops*<br>16. Multivitamin syrup/drops*<br>17. Intravenous (Anti-D) immunoglobulin (for rhesus disease) *
**Intervention coverage**	
Appropriate antibiotic prescription	Dose of gentamicin and/or penicillin as per national guidelines, allowing for ±20% margin of error
Correct oxygen prescription	Correct route and prescribed to patients requiring oxygen treatment as per recorded signs and symptoms
Correct fluids and feeds volume	As per national guidelines, allowing for ±20% margin of error
**Process quality-adjusted coverage**	
**Quality: process of care**	
Documentation of newborn characteristics	Document 9 characteristics: age, sex, mode of delivery, weight, gestational age, Apgar score at 5 min, HIV status, diagnosis, outcome

Documentation of signs and symptoms	Signs (evaluation on admission): Temperature, bulging fontanelle, can suck or breastfeed, reduced mobility or floppy, respiratory rate, in drawing, grunting, central cyanosis<br>Symptoms (history): Prolonged rupture of membranes (ROM) (>18 h), fever, difficulty breathing, severe vomiting, difficulty feeding or breastfeeding, convulsions, partial or focal fits, apnoea
Evidence of monitoring	Treatment sheet available and filled, vital signs chart available and filled, evidence of weight monitoring
**Quality: interpersonal**	
**User-adherence adjusted coverage**	
**Outcomes-adjusted coverage**	

Table S6 Exclusive breastfeeding

Study (data sources)	Nguhiu et al. 2017 (40) (DHS)
**Intervention**	**Exclusive Breastfeeding**
**Target population**	
Children	Children 0-5 mnths
**Service contact**	
Breastfed	Breastfed in last 24 hours
**Input-adjusted coverage**	
**Intervention coverage**	
**Process quality-adjusted coverage**	
**User-adherence adjusted coverage**	
Adherence to guidelines	Respondent reported exclusively breastfeeding in preceding 24 h AND no other complementary feed offered
**Outcome-adjusted coverage**	

Table S7 Postnatal care

Study (data sources)	Baker et al. 2015 (55) (HH survey; HF assessment; HCW interview)	Carvajal-Aguirre et al 2017 (31) (DHS)	Hategeka et al. 2020 (52) (DHS)	Marchant et al. 2015 (34) (HH survey; HF assessment; HCW interview)		Munos et al. 2018 (43) (MICS; HF assessment; HCW interview)		Okawa et al. 2019a (49) (HH survey)	Okawa et al. 2019b (46) (HH survey)	Shibanuma et al. 2018 (58) (HH survey)
**Intervention**	**PPC for mother in a health facility within 48hrs of delivery**	**Postnatal health check within 48 hours**	**Postpartum check-up before discharge**	**(1) PPC for mother within 48 hours of birth**	**(2) PNC for newborn within 48 hours**	**(1) postnatal care within 48 hours: structural quality**	**(2) postnatal care within 48 hours: process quality**	**PNC for mother & newborn**	**PNC for mother & newborn**	**PNC within 48 hrs & around 2 & 6 wks post-delivery**
**Target population**										
	Women with live birth in 12mnths prior to survey	Surviving children under 2 years of age at time of survey	Women aged 15-49 yrs with at least 1 live birth in 5 yrs preceding the survey	Women aged 13-49 who had a live birth in 12 mnths prior to survey	Women aged 13-49 who had a live birth in 12 mnths prior to survey	Women report a live birth in the 2 yrs preceding the survey	Women report a live birth in the 2 yrs preceding the survey	Women between 6 wks -12 mnths postpartum	Women aged 15-49 yrs delivered in 2 yrs prior to survey	Women aged 15-49 yrs delivered in 2 yrs prior to survey
**Service contact coverage**										
Facility based care	Women reported giving birth in a facility	Women reported skilled birth attendant at birth	Delivered in a health facility during most recent pregnancy leading to a live birth	Women reported at least 1 postpartum contact within 48 hours of birth	Women reported newborn had at least 1 postnatal check within 48 hours of birth	Sought facility based care within two days of birth	Sought facility based care within two days of birth	At least 3 contacts for PNC with healthcare providers, including first contact within 24 hours postpartum	Three contacts with healthcare providers within 48hours, at 1 week (3–10 days) and at 6 weeks (36–48 days) postpartum	Received PNC within 48 hours and around 2 and 6 weeks post-delivery
**Input-adjusted coverage**										
**Inputs: service infrastructure**										
Emergency transport						Emergency transportation				
**Inputs: Staffing, training & guidelines**										
Supervision						Supervised with observation				
Staff availability						24-hour staff coverage				
Trained: counselling PNC						Trained on counselling for PNC				
Trained: management of complications in pregnancy						Trained on management of complications of pregnancy				
Trained: PMTCT						Trained on PMTCT				
Trained: nutrition						Trained on nutrition counselling for newborn of mother with AIDS				
Trained: feeding						Trained on infant and young child feeding for HIV+ mothers				
Trained: PMTCT prophylaxis						Trained on PMTCT prophylactic treatment				
**Inputs: Supplies & commodities**										
Antibiotics						Injectable antibiotics				
Blood pressure apparatus						Digital BP machine or manual sphygmomanometer with stethoscope observed in service area				
Iron supplements/tablets	Facility with iron supplements available									

Scale						Infant weighing scale observed in service area				
Stethoscope						Stethoscope				
Thermometer						Thermometer				
**Inputs: service availability**										
ARVs						ARV to mother<br>ARV to infant				
Counselling: Breastfeeding						Breastfeeding counselling				
Counselling: HIV prevention						HIV preventive counselling				
Counselling: HIV test						HIV test counselling				
Counselling: ITNs						Counselling on ITNs				
Counselling: newborn care						Newborn cares counselling				
HIV test						HIV test				
Inpatient						Inpatient				
Counselling: family planning						FP counselling				
**Intervention coverage**										
Anaemia								Women report anaemia checked	Haemoglobin assessment	
Baby weighed		Women reported newborn weighed at birth			Women reported weight checked					
Family planning							Family planning method			
Immunisation: BCG vaccination		Women reported BCG vaccination						Women reported BCG immunisation given	BCG immunisation	Received necessary immunisations
Immunisation: Hepatitis B								Women reported Hepatitis B immunisation given		
Immunisation: Oral polio vaccine		Women reported polio vaccination at birth							Oral polio vaccine	
Iron folate tablets							Iron tablets	Women reported iron folate tablets prescribed		
Vitamin A given to mother							Vitamin A capsules	Women report Vitamin A tablets prescribed	Vitamin A supplement	
Vitamin B given to mother								Women report Vitamin B tablets prescribed		
**Process quality-adjusted coverage**										
**Quality: process**										
Breastfeeding checked							Other assessments (breastfeeding, child's weight/growth, etc.)	Women report breastfeeding checked	Breastfeeding problem check<br>Breastfeeding difficulties check	
Counselled on breastfeeding, thermal care and danger signs				Women reported being counselled on breastfeeding, thermal care and danger signs			Advice on preventive PNC (keeping baby warm, cord, breastfeeding, FP, postpartum hygiene, ITN)			Learnt about post-delivery complications among women and children
Counselled on danger signs, nutrition and family planning				Women reported being counselled on danger signs, nutrition, and family planning			Advice to seek care if child has any danger sign (fever, cold, difficulty to breastfeed, rapid/difficult	Women reported family planning counselling		Learnt about nutrition, anaemia and breast feeding

							breathing, etc.) Advice to seek care if mother has any danger sign (sudden and profuse bleeding, vomiting, faintness, fever, etc.)			
Maternal assessment							Initial assessment of mother's health (signs and symptoms since delivery)		Fundal height assessment Perineum/Lochia assessment	
Maternal blood pressure								Women reported blood pressure measured	Women reported blood pressure assessed	
Maternal temperature								Women reported temperature measured	Women reported temperature measured	
Newborn assessment					Women reported cord checked		Initial assessment of child's health (signs and symptoms since childbirth)		Umbilical cord/bleeding check	
Newborn examined					Women reported newborns body examined for danger signs		Physical examination of the child	Women report physical examination of newborn	General physical examination	
Newborn temperature measured								Women report newborn temperature measured	Newborn temperature measurement	
Postpartum check			Women report examined or asked questions about their health before discharge	Women reported breasts and bleeding checked			Physical examination of the mother	1. Women report breast and nipple checked 2. Women report vaginal healing checked 3. Women report uterus checked 4. Women report lochia checked 5. Women report emotional status checked	Bleeding check	
Timely postpartum check	Women report being checked within 48 hours of delivery	Women reported postnatal care for newborn AND mother within 2 d of birth	Women report examined or asked questions about their health within one hour of delivery							
**Quality: interpersonal**										
**User-adherence adjusted coverage**										
Adherence to guidelines		Women reported early initiation of breastfeeding AND no prelacteal feed during first three days of life								
**Outcomes-adjusted coverage**										

Table S8 Sick child care

Study (data sources)	Carter et al. 2018 (45) (HH survey; HF survey; HCW knowledge assessment)	Hategeka et al. 2020 (52) (DHS)			Koulidiati et al. 2018 (42) (HH Survey, HF assessment, Observation)	Leslie et al. 2017 (33) (DHS/MICS, SPA)	Leslie et al. 2019 (37) (ENSANUT [national survey]; HMIS)		Millar et al. 2014 (50) (HH survey)	Munos et al. 2018 (43) (MICS; HF assessment; Observations)		Nguhiu et al. 2017 (40) (DHS; SPA)	Nguyen et al. 2021 (44) (DHS, SPA)	Smith et al. 2010 (51) (HH survey)
**Health service/intervention**	**Treatment of diarrhoea, fever and/or ARI**	**(1) Treatment of pneumonia**	**(2) Treatment of diarrhoea**	**(3) Treatment of malaria**	**Treatment of illness**	**Treatment of diarrhoea, fever or ARI**	**(1) Treatment of diarrhoea**	**(2) Treatment of respiratory conditions**	**Care seeking and treatment for malaria**	**(1) sick child care (fever, cough or diarrhoea): structural quality**	**(2) sick child care (fever, cough or diarrhoea): process quality**	**Quality of primary care for children: treatment of ARI and/or fever**	**Sick child care (diarrhoea or ARI)**	**Treatment for malaria**
**Target population**														
	Mothers of children <5 years who reported at least one DHS illness (diarrhoea, fever, ARI or a combination)	Parental report children <5 who, in the past 2 weeks, have suffered from symptoms consistent with pneumonia (a cough accompanied by short, rapid breathing and difficulty breathing as a result of a problem in the chest)	Parental report children <5 who had diarrhoea in the past 2 weeks	Parental report children <5 who had fever in the past 2 weeks	Children under 5 years of age that experienced an illness episode during the 4 weeks prior to the survey date.	Children under 5 who had experienced diarrhoea, fever or acute respiratory illness in the prior 2 weeks	Parental report of child under 5 experiencing at least 3 days of diarrhoea or diarrhoea plus fever	Parental report of symptom of flu, cough, bronchitis, sore throat or pain in his/her ears past 2 weeks in child under 5	Mothers of children aged 0-59 mnths who reported a fever in the last 2 wks	Mothers of children under-5 yrs who reported child had fever, cough or diarrhoea in 2 wks before survey	Mothers of children under-5 yrs who reported child had fever, cough or diarrhoea in 2 wks before survey	All children under 5 yrs reported to have had acute respiratory illness and/or fever in the preceding 2 wks	All children alive between 0-59 mnths who had diarrhoea or ARI in the last 2 weeks	All children under 5 yrs with fever in the last two weeks
**Service contact**														
Seek care	Mother reported seeking care from any provider	Taken to medical facility for treatment (including public sector and medical private sector facilities, except for pharmacies and traditional practitioners)	Taken to medical facility for treatment (including public sector and medical private sector facilities, except for pharmacies and traditional practitioners)	Taken to medical facility for treatment (including public sector and medical private sector facilities, except for pharmacies and traditional practitioners)	Sought care at the nearest facility	An interaction with a health facility or formal provider	Visits to IMSS family medicine clinic due to diarrhoea	Visits to IMSS family medicine clinic due to non-chronic respiratory condition	Sought treatment at formal and informal treatment locations	Source of care where advice or treatment was sought.	Source of care where advice or treatment was sought.	Advice on treatment was sought from a medical provider	Sought care from a medical provider	Child taken for treatment or advice to community delivery point, public health facility or retail delivery point
**Inputs**														
**Inputs: service infrastructure**														
Ambulance/Emergency transport					Functional emergency vehicle available									
Patient waiting room					Patient waiting room available.									
Power/Electricity					Functional electricity source available									
Sanitation					Functional toilet facilities available									

Water supply					Functional water source and soap available in the consultation room									
**Inputs: Staffing, training & guidelines**														
Qualified HCW					Observed cases attended by a qualified HCW									
IMCI guidelines	Guidelines (IMCI guidelines or relevant guidelines or job aid available)									IMCI guidelines observed in service area			IMCI guideline: national guidelines for IMCI, IMCI chart booklet, IMCI card, other visual aids	
HCW Knowledge: breathing difficulties	Average performance on case scenarios				Vignette-based scenario: Breathing difficulties in a 1-year-old with simple pneumonia									
HCW knowledge: severe dehydration					Vignette-based scenario: Viral illness with severe dehydration in a 2-year-old									
HCW knowledge: lethargy					Vignette-based scenario: Lethargic 1-month-old									
Supervision	Supervision (received supervision visit with case management observation in past 3 months)									Supervised with observation				
Trained ARI										Trained on ARI diagnosis and management				
Trained diarrhoea management										Trained on diarrhoea management				
Trained iCCM										Trained on iCCM (CHWs)				
Trained IMCI	Training (at least one staff member with IMCI or relevant training)				Observed cases attended by a HCW trained in IMCI					At least one staff member providing the service trained in some aspect of IMCI in the last two years			Staff with any training on IMCI Guidelines	

Trained malaria diagnosis & management										Trained on malaria diagnosis and management				
Trained nutrition										Trained on nutritional assessment				
Trained paediatric HIV diagnosis & management										Trained on paediatric HIV diagnosis and management				
**Inputs: Supplies & commodities**														
ACT	Basic medicine: Artemisinin combination therapy (ACT)									Artemisinin combination therapy (ACT) any child dosage or formulation. Observed in service area OR where routinely stored; in stock with at least one valid.				
Anthelmintic													Albendazole/ mebendazole	
Antibiotic	Basic medicine: Oral antibiotic				Antibiotics in stock; ceftriaxone in stock					Cotrimoxazole or amoxicillin any child dosage or formulation. Observed in service area OR where routinely stored; in stock with at least one valid.				
Clock/timer										Timer				
Haemoglobin													Diagnostic capacity: Haemoglobin	
Injectable antibiotics	Severe/complicated illness medicines: Injectable antibiotics									Injectable antibiotics				
Injectable antimalarials	Severe/complicated illness medicines: Injectable quinine or artesunate									Injectable antimalarials				
Iron													Iron tablet	
IV fluids	Severe/complicated illness medicines: IV fluids				Dextrose solutions or dextrose containing intravenous fluids in stock									
Microscopy supplies	Diagnostics: General microscopy									Microscopy supplies				

	(functioning microscope and slides)													
ORS	Basic medicine: Oral rehydration solution				Isotonic fluid or Oral rehydration solution and nasogastric tube					Oral Rehydration Salts (ORS) sachets any child dosage or formulation. Observed in service area OR where routinely stored; in stock with at least one valid.			ORS	
Paracetamol					Paracetamol suppository in stock									
RDT	Diagnostics: Malaria Diagnostic (RDTs or microscopy)				Diagnostic: Malaria testing supplies in stock					Malaria rapid test or smear (microscope, slides, and stain). Able to conduct the test on-site (in the facility) and functioning equipment and reagents needed to conduct the test are observed on-site on the day of the survey. In area where tests for child health are carried out or anywhere in the facility where laboratory testing is routinely conducted				
Scale	Diagnostics: Malnutrition Diagnostic (MUAC or Scale + Height board + Growth chart)				Functional scale available					Child and infant scale. Weight gradations at minimum 250 grams and 100 grams. Observed availability, reported functionality, and in service area or adjacent area.		Observed availability of at least one working weighing scale	Scale (observed)	
Stethoscope	Diagnostics: ARI Diagnostic (stethoscope									Stethoscope. Observed availability, reported				

	or respiratory timer)									functionality, and in service area or adjacent area.				
Timer										Timer				
Thermometer					Functional thermometer available					Thermometer. Observed availability, reported functionality, and in service area or adjacent area.		Observed availability of thermometer		
Vitamin A													Vitamin A	
Zinc	Basic medicine: Zinc									Zinc sulphate tablets, dispersible tablets or syrup any child dosage or formulation. Observed in service area OR where routinely stored; in stock with at least one valid.			Zinc tablet/zinc sulphate syrup	
**Inputs: service availability**														
ARI	Diagnosis and treat ARI (by pathology)													
Care for children under-5										Facility offering preventive and curative care for children under 5				
Diarrhoea	Diagnosis and treat diarrhoea (by pathology)													
IMCI										Facility offering IMCI services				
Malaria	Diagnosis and treat malaria (by pathology)													
Malnutrition	Diagnosis and treat malnutrition (by pathology)									Facility offering malnutrition diagnosis and treatment				
Referral capacity	Facilitated referral capacity													
**Intervention**														
ACT														Child received an ACT
Antibiotics		Mother reported child												

		received antibiotic pills, syrup or injections												
Antimalarial									Mother reported child took Artemisinin-based combination therapy (ACT) if the blood test is positive					Child received an anti-malarial
Deworming medication						Children 2-59mnths: deworming medication								
Malaria test				Mother reported blood taken from child's finger or heel for testing					Mother reported child received diagnostic blood test (either microscopy or RDT)					
ORT			Mother reported child received oral rehydration therapy (from oral rehydration salts (ORS), pre-packaged ORS liquid or other homemade fluids)											
Vitamin A dosage						All children: Vitamin A dosage								
**Process quality-adjusted coverage**														
**Quality: process of care**														
Check Convulsions						All children: history taking convulsions								
Check danger signs					Provider observed to ask for at least two general danger signs per IMCI guidelines						Observed checked for 3 danger signs			
Check diarrhoea					Provider observed to ask for presence of diarrhoea									
Check edema of feet						Children 2-59mnths: edema of feet								
Check Fever, cough/difficulty breathing, diarrhoea					Provider observed to ask for presence of fever Provider	Children 2-59mnths: history taking cough or difficult breathing					Observed checked for fever, cough/difficulty breathing, and diarrhoea			

					observed to ask for presence of cough	All children: history taking diarrhoea and blood in stool (dysentery) Children 2-59mnths: history taking fever								
Check health card											Observed checked health card			
Check immunisations					Provider observed to check child's current vaccination status	All children: checked immunisation card or immunised						Observed or HCW reported routine assessment of immunisation status		
Check mouth (thrush in IMCI)						Children <2mnths: mouth (thrush IMCI)								
Check pallor					Provider observed to check for signs of anaemia (conjunctivae, palms)	Children 2-59mnths: pallor					Observed checked for palmar or conjunctival pallor			
Check Vomiting						Children 2-59mnths: history taking vomiting								
Check: Ear problems					Provider observed to ask for presence of ear problems	Children 2-59mnths: history taking ear problems								
Check: Inability to drink						All children: history taking inability to drink anything								
Check: Maternal HIV status						All children: history taking Maternal HIV status								
Check: Normal feeding						Children <2mntths: history taking normal feeding pattern								
Check: Sick feeding						Children <2mntths: history taking sick feeding pattern								
Described danger signs requiring return to facility						All children: Described danger signs requiring return to facility								
Directions for feeding						All children: Directions for feeding								

Explained how to administer prescribed medication						All children: Explained how to administer prescribed medication								
Gave diagnosis						Children 2-59mths: Gave diagnosis								
Plotted weight on chart						All children: plotted weight on chart								
Records												Observed or HCW reported keeping of individual patient records		
Scheduled/discussed return visit						All children: Scheduled/discussed return visit								
Take temperature					Provider observed to ask child's temperature	All children: temperature						Observed or HCW reported routine temperature taking and recording		
Timely treatment									Mother reported prompt care-seeking within the first 24 hours of symptom onset					Sought care within 48 hours
Treated according to guidelines											Observed child correctly treated per provider diagnosis	HCW reported providers follow IMCI guidelines to assess and treat sick children		
Weighed					Provider observed to checks child's weight	All children: weight					Observed weighed and weight plotted on growth chart	Observed or HCW reported child weight taken		
**Quality: experience of care**														
**User adherence**														
**Outcomes-adjusted coverage**														
							Visits to IMSS family medicine clinics that did not result in hospitalization due to diarrhoea for children under age 5	Visits to IMSS family medicine clinics that did not result in hospitalization due to non-chronic respiratory condition for							

								children under age 5						

Table S9 Complementary feeding

Study (data sources)	Aaron et al. 2016 (63) (HH)	Leyvraz et al. 2016a (61) (HH)	Leyvraz et al. 2016b (62) (HH)	Leyvraz et al. 2018 (60) (HH)	Nguyen et al. 2016 (57) (HH)
**Intervention**	**Complementary feeding supplement**	**Fortified Complementary Food**	**Fortified Complementary Food**	**Home fortification with micronutrient powders**	**Fortification of complementary Foods with Micronutrient Powders**
**Target population**					
Children	Children aged 6-24 months<br>(1) all children<br>(2) children at-risk based on poverty, poor maternal dietary diversity and suboptimal feeding practices	Children aged 0-23 months<br>(1) all children<br>(2) children at-risk based on poverty and poor feeding practices	Children aged 0-35 months<br>(1) all children<br>(2) children at-risk based on poverty and poor feeding practices	Children aged 6-23 months<br>(1) all children<br>(2) children at-risk based on poverty and poor feeding practices	Children aged 6-59 mnths
**Service contact**					
Heard product	Caregiver ever heard of the product	Caregiver ever heard of Farinor or Nutribon	Caregiver has ever heard of Bal Amrutham	Caregiver has ever heard MNP	Caregiver has ever heard of or seen of the National Institute of Nutrition-specific micronutrient powder (Bibomix)
Received product			Caregiver ever received product		
**Input-adjusted coverage**					
**Intervention coverage**					
Consumed fortified food	Child ever been fed the product	Caregiver ever fed child fortified complementary food (Farinor or Nutribon)	Caregiver ever fed child fortified complementary food	Child ever given micronutrient powder	Child ever fed product
**Process quality-adjusted coverage**					
**User-adherence adjusted coverage**					
Partial		Child fed Farinor or Nutribon at least once in past mnth	Target child consumes Bal Amrutham sometimes or always	Child consumed at least 1 sachet of MNP in the past week	Consumed at least 1 sachet over the past week.
Effective	Child fed product at least one in the previous seven days	Child fed Farinor or Nutribon at least once in past 7 days	Child always consumes Bal Amrutham	Child consumed at least 3 sachets of MNP in past week	Consumed 3 or more sachets over the past week.
**Outcome-adjusted coverage**					

Table S10 Growth monitoring

Study (data sources)	Nguyen et al. 2021 (44) (DHS, SPA)
**Intervention**	**Growth monitoring**
**Target population**	
Children	All children alive between 0-59 mnths
**Service contact**	
Attend health service	All children who had diarrhoea or ARI symptoms for whom care was sought from a medical provider
**Input-adjusted coverage**	
**Inputs: Staffing, training & guidelines**	
Guidelines	Guidelines for growth monitoring (observed)
Training	Staff with any training on growth monitoring
**Inputs: Supplies & commodities**	
Growth chart	Growth chart (observed)
Length or height board	Length or height board (observed)
Scale	Child scale (observed)
Tape for measuring head	Tape for measuring head (observed)
**Intervention coverage**	
**Process quality-adjusted coverage**	
**User-adherence adjusted coverage**	
**Outcome-adjusted coverage**	

Table S11 Insecticide treated bed net (ITN)

Study (data sources)	Nguhiu et al. 2017 (40) (DHS)
**Intervention**	**Malaria prevention**
**Target population**	
	Children and pregnant women
**Service contact**	
	Live in household that own an ITN
**Input-adjusted coverage**	
**Intervention coverage**	
	Self-report slept under ITN the night before
**Process quality-adjusted coverage**	
**User adherence-adjusted coverage**	
**Outcomes-adjusted coverage**	

Table S12 Vaccination

Study (data sources)	Mmanga et al. 2021 (59) (DHS)	Mokdad et al. 2015 (35) (HH survey)				Nguhiu et al. 2017 (40) (DHS; SPA)	Sheff et al. 2020 (48) (HH survey)
**Intervention**	**Complete immunisation**	**(1) Timely MMR vaccine**	**(2) MMR vaccine: facility readiness (MMR in stock)**	**(3) MMR vaccine: facility readiness (MMR stock-out in last 3 mnths)**	**(4) MMR vaccine: facility readiness (ORS in stock)**	**Quality of primary care for children: complete set of basic vaccines**	**Complete set of basic vaccines: quality coverage**
**Target population**							
	Children aged 12–23 months	Children 13.5–59 months with a vaccination card	Children 13.5–59 months with a vaccination card	Children 13.5–59 months with a vaccination card	Children 13.5–59 months with a vaccination card	All children alive between 12-23 mnths	Children aged 12-23 mnths
**Service contact**							
Vaccination	Received either BCG or Penta1 vaccine during the past year	At least one caregiver-reported or card-documented MMR dose.	At least one caregiver-reported or card-documented MMR dose.	At least one caregiver-reported or card-documented MMR dose.	At least one caregiver-reported or card-documented MMR dose.	Received the complete set of vaccines as outlined in the Kenya Ministry of Health National Vaccination Schedule i.e. BCG, three doses of oral or intravenous Polio, three doses of Diphtheria, Pertussis, Tetanus, Hepatitis B and Haemophilus Influenza type B pentavalent vaccine, three doses of pneumococcal vaccine (from Jan 2011 onwards), and Measles vaccines	Received BCG vaccination
**Inputs-adjusted coverage**							
**Inputs: Supplies & commodities**							
MMR			MMR in stock on day of survey	MMR in stock on day of survey & stock out in three months prior to survey			
ORS					ORS in stock on day of health facility survey		
Scale						Observed at least one working infant weighing scale or child scale present	
Thermometer						Observed at least one thermometer	
**Intervention coverage**							
Additional vaccines	Received Penta 3 vaccine during past year						Received all three doses of the DPT vaccine
	Received with MR1 during the past year						
**Process quality-adjusted coverage**							
**Quality: process of care**							
Check immunisations						Observed or HCW reported routine assessment of immunisation status	
Records						Observed or HCW reported keeping of individual patient records	
Temperature						Observed or HCW reported child's temperature taken	
Weighed						Observed or HCW reported child weight taken	
Guidelines						HCW reported providers follow IMCI guidelines to assess and treat sick children	
Recommended according to schedule	Received BCG, OPV3, Penta3, PCV3, Rota2 and MCV1 vaccines	MMR vaccine given within recommended interval: administered between 11.5 and 13.5 months	MMR vaccine given within recommended interval: administered between 11.5 and 13.5 months	MMR vaccine given within recommended interval: administered between 11.5 and 13.5 months	MMR vaccine given within recommended interval: administered between 11.5 and 13.5 months		Received all of the basic vaccinations by 24 months: one dose of BCG at birth, three doses of the oral polio vaccine (excluding the dose given at birth), three doses of a DPT containing vaccine and hepatitis B vaccine at 6, 10,

							and 14 weeks, and one dose of the measles vaccine.
**User-adherence adjusted coverage**							
**Outcomes-adjusted coverage**							
